# EPAS1 Attenuates Atherosclerosis Initiation at Disturbed Flow Sites Through Endothelial Fatty Acid Uptake

**DOI:** 10.1161/CIRCRESAHA.123.324054

**Published:** 2024-09-05

**Authors:** Daniela Pirri, Siyu Tian, Blanca Tardajos-Ayllon, Sophie E. Irving, Francesco Donati, Scott P. Allen, Tadanori Mammoto, Gemma Vilahur, Lida Kabir, Jane Bennett, Yasmin Rasool, Charis Pericleous, Guianfranco Mazzei, Liam McAllan, William R. Scott, Thomas Koestler, Urs Zingg, Graeme M. Birdsey, Clint L. Miller, Torsten Schenkel, Emily V. Chambers, Mark J. Dunning, Jovana Serbanovic-Canic, Francesco Botrè, Akiko Mammoto, Suowen Xu, Elena Osto, Weiping Han, Maria Fragiadaki, Paul C. Evans

**Affiliations:** 1 School of Medicine and Population Health, INSIGNEO Institute, and the Bateson Centre (D.P., S.T., S.E.I., J.S.-C.), University of Sheffield, United Kingdom.; 2 School of Medicine and Population Health, Sheffield Institute for Translational Neuroscience (S.P.A.), University of Sheffield, United Kingdom.; 3 Sheffield Bioinformatics Core, School of Medicine and Population Health (E.V.C., M.J.D.), University of Sheffield, United Kingdom.; 4 Centre for Biochemical Pharmacology (S.T., B.T.-A., P.C.E.), William Harvey Research Institute, Barts and the London School of Medicine and Dentistry, Queen Mary University of London, United Kingdom.; 5 Centre for Translational Medicine and Therapeutics (M.F.), William Harvey Research Institute, Barts and the London School of Medicine and Dentistry, Queen Mary University of London, United Kingdom.; 6 Laboratorio Antidoping, Federazione Medico Sportiva Italiana, Rome, Italy (F.D., F.B.).; 7 Department of Pediatrics, Department of Pharmacology and Toxicology (T.M.), Medical College of Wisconsin, Milwaukee.; 8 Department of Pediatrics and Department of Cell Biology, Neurobiology and Anatomy (A.M.), Medical College of Wisconsin, Milwaukee.; 9 Institut de Recerca Hospital de la Santa Creu i Sant Pau, IIB-Sant Pau, and CIBERCV (Centro de Investigación en Red de Enfermedades Cardiovasculares)-Instituto de Salud Carlos III, Barcelona, Spain (G.V.).; 10 Medical Research Council (MRC) Laboratory of Medical Sciences, London, United Kingdom (L.K., J.B., Y.R., G.M., L.M., W.R.S.).; 11 Institute of Clinical Sciences, Faculty of Medicine (Y.R., G.M., L.M., W.R.S.), Imperial College London, United Kingdom.; 12 National Heart and Lung Institute (D.P., G.M.B.), Imperial College London, United Kingdom.; 13 Department of Surgery, Bariatric Center, Limmattal Hospital, Schlieren, Switzerland (C.P., T.K., U.Z.).; 14 Center for Public Health Genomics, Department of Public Health Sciences, University of Virginia, Charlottesville (C.L.M.).; 15 Department of Engineering and Mathematics, Sheffield Hallam University, United Kingdom (T.S.).; 16 Department of Endocrinology, Institute of Endocrine and Metabolic Disease, The First Affiliated Hospital of University of Science and Technology of China (USTC), Division of Life Sciences and Medicine, Clinical Research Hospital of Chinese Academy of Sciences (Hefei), University of Science and Technology of China, Hefei, China (S.X.).; 17 Institute of Clinical Chemistry University Hospital and University of Zurich, Switzerland (E.O.).; 18 Division of Physiology and Pathophysiology, Otto Loewi Research Center, Medical University of Graz, Austria (E.O.).; 19 Institute of Molecular and Cell Biology, Agency for Science, Technology and Research (A*STAR), Singapore (W.H.).

**Keywords:** atherosclerosis, diet, high-fat, endothelial cells, obesity, plaque, atherosclerotic

## Abstract

**BACKGROUND:**

Atherosclerotic plaques form unevenly due to disturbed blood flow, causing localized endothelial cell (EC) dysfunction. Obesity exacerbates this process, but the underlying molecular mechanisms are unclear. The transcription factor EPAS1 (HIF2A) has regulatory roles in endothelium, but its involvement in atherosclerosis remains unexplored. This study investigates the potential interplay between EPAS1, obesity, and atherosclerosis.

**METHODS:**

Responses to shear stress were analyzed using cultured porcine aortic EC exposed to flow in vitro coupled with metabolic and molecular analyses and by en face immunostaining of murine aortic EC exposed to disturbed flow in vivo. Obesity and dyslipidemia were induced in mice via exposure to a high-fat diet or through Leptin gene deletion. The role of *Epas1* in atherosclerosis was evaluated by inducible endothelial *Epas1* deletion, followed by hypercholesterolemia induction (adeno-associated virus-PCSK9 [proprotein convertase subtilisin/kexin type 9]; high-fat diet).

**RESULTS:**

En face staining revealed EPAS1 enrichment at sites of disturbed blood flow that are prone to atherosclerosis initiation. Obese mice exhibited substantial reduction in endothelial EPAS1 expression. Sulforaphane, a compound with known atheroprotective effects, restored EPAS1 expression and concurrently reduced plasma triglyceride levels in obese mice. Consistently, triglyceride derivatives (free fatty acids) suppressed EPAS1 in cultured EC by upregulating the negative regulator PHD2. Clinical observations revealed that reduced serum EPAS1 correlated with increased endothelial PHD2 and PHD3 in obese individuals. Functionally, endothelial EPAS1 deletion increased lesion formation in hypercholesterolemic mice, indicating an atheroprotective function. Mechanistic insights revealed that EPAS1 protects arteries by maintaining endothelial proliferation by positively regulating the expression of the fatty acid-handling molecules CD36 (cluster of differentiation 36) and LIPG (endothelial type lipase G) to increase fatty acid beta-oxidation.

**CONCLUSIONS:**

Endothelial EPAS1 attenuates atherosclerosis at sites of disturbed flow by maintaining EC proliferation via fatty acid uptake and metabolism. This endothelial repair pathway is inhibited in obesity, suggesting a novel triglyceride-PHD2 modulation pathway suppressing EPAS1 expression. These findings have implications for therapeutic strategies addressing vascular dysfunction in obesity.

**Novelty and Significance**

**What Is Known?**

- Atherosclerotic plaques form in arteries at regions of disturbed blood flow, which alters endothelial cell (EC) activation and proliferation.
- EPAS1 (HIF2A) regulates endothelial cells in developing vessels, but its involvement in atherosclerosis has remained unexplored.

**What New Information Does This Article Contribute?**

- EPAS1 is activated by disturbed flow leading to enrichment at disease-prone regions of arteries.
- EPAS1 protects against atherosclerosis by promoting fatty acid beta-oxidation, which maintains endothelial proliferation.
- Endothelial EPAS1 expression is reduced in murine and human obesity.

Atherosclerotic plaques preferentially develop in regions of arteries experiencing disturbed blood flow, leading to endothelial cell dysfunction and injury. The transcription factor EPAS1 (HIF2A) is known to regulate endothelial cells during development, but its specific role in atherosclerosis has not been previously elucidated. Our study demonstrates that EPAS1 is activated by disturbed flow and accumulates in disease-prone areas of arteries. Through a combination of in vitro and in vivo experiments, we show that EPAS1 safeguards against atherosclerosis by promoting endothelial cell proliferation, a critical process for maintaining vascular homeostasis. Mechanistically, we show that EPAS1 facilitates endothelial proliferation by upregulating LIPG (endothelial type lipase G) and CD36 (cluster of differentiation 36), which enhance fatty acid uptake and subsequent β-oxidation. Interestingly, we found that EPAS1 expression was significantly diminished in murine models of obesity, potentially due to elevated triglycerides activating the negative regulator PHD3. Clinical data supported these findings, revealing lower plasma EPAS1 levels and increased endothelial PHD3 expression in obese individuals. In conclusion, our study highlights the protective function of endothelial EPAS1 against atherosclerosis in disturbed flow regions, which is diminished in the context of obesity. The identification of a novel triglyceride-PHD3-EPAS1 axis opens new avenues for therapeutic approaches targeting vascular dysfunction associated with obesity.

**In This Issue, see p 803**

**Meet the First Author, see p 804**

**Editorial, see p 838**

Atherosclerotic plaques exhibit uneven development, accumulating at arterial regions exposed to disturbed blood flow. These hemodynamic conditions generate mechanical wall shear stress with low magnitude and oscillations in direction that induce localized endothelial cell (EC) dysfunction, which drives plaque initiation.^1^ The global obesity epidemic is associated with metabolic abnormalities including dyslipidemia and hyperglycemia that are drivers of atherosclerosis. Disturbed flow enhances the sensitivity of EC to dyslipidemia and hyperglycemia^2,3^; however, the molecular mechanisms underlying this link remain poorly understood.

The preservation of endothelial homeostasis relies on an intricate interplay of transcription factors regulating metabolic equilibrium.^3,4^ Notably, the hypoxia-inducible factor (HIF) transcription factors, HIF1A and EPAS1 (HIF2A), play a central role in this regulatory network.^5^ Under normal oxygen conditions, these proteins undergo prolyl hydroxylation coordinated by prolyl hydroxylase domain (PHD) proteins, which triggers ubiquitination and proteasomal degradation.^6^ However, they accumulate in response to ischemia-induced hypoxia and inflammation, which disrupt the degradation pathway.^7^ HIF1A has a well-established role in angiogenesis by coordinating the expression of growth factors and glycolytic regulators in sprouting EC.^8^ It also accumulates in arteries at sites of disturbed flow, where it promotes atherosclerosis through glycolysis and inflammation.^9–11^ The function of EPAS1 in vascular biology is less explored. However, EPAS1 is known to promote vascularization by stabilizing newly formed microvascular networks and by driving arteriogenesis.^12–15^ EPAS1 regulates several other physiological processes, including embryogenesis, erythropoiesis, tumorigenesis, and liver metabolism.^16–19^ However, the potential role of EPAS1 in arterial homeostasis, EC mechanical responses, and atherosclerosis remains unclear.

Here, we investigate the role of EPAS1 in atherosclerosis development, particularly at disturbed flow sites. We demonstrate that EPAS1 is enriched in these regions, where it limits atherosclerosis by promoting fatty acid metabolism to enhance endothelial homeostasis. Importantly, our findings reveal that obesity downregulates EPAS1 expression in atheroprone areas, establishing EPAS1 as a crucial intermediate link between obesity and endothelial responses to shear stress. Our research sheds light on the intricate molecular mechanisms connecting obesity, disturbed flow, and atherosclerosis, potentially opening new avenues for further exploration and potential therapeutic interventions.

## METHODS

### Data Availability

Annotated data have been deposited in the Gene Expression Omnibus database (accession GSE274418).

Detailed methods are available in the Supplemental Material.

### Mice

Mice with inducible deletion of *Epas1* in EC were generated by crossing *Epas1*^*fl/fl*20^ mice with *CDH5*^*Cre-ERT2*^ mice^21^ and genotyped by polymerase chain reaction (PCR; Table S1). Cre-dependent gene deletion was induced using tamoxifen. Hypercholesterolemia was induced by IP injection of an adeno-associated virus (AAV) containing a gain-of-function mutated version of proprotein convertase subtilisin/kexin type 9 (rAAV8-D377Y-mPCSK9) gene followed by a high fat diet (HFD).^22^ Obesity was analyzed by exposing *Lep*^*ob/ob*^ mice and littermate control mice (*Lep*^*WT*^) to a standard rodent chow diet. Obesity was induced in C57BL/6N wild-type (WT) mice by exposure to a western diet containing 60% fat,^23^ and in C57BL/6J by exposure to HFD containing 45% fat. Blood pressure measurements were made by plethysmography. Hyperglycemia was induced in C57BL/6J mice using streptozotocin. Male and female mice were analyzed. Animal care and experimental procedures were performed under licenses issued by the UK Home Office, and local ethical committee approval was obtained. All animal procedures conformed to the guidelines from Directive 2010/63/EU of the European Parliament on the protection of animals used for scientific purposes and to Institutional Animal Care and Use Committee guidelines. All experiments involving animals in Singapore were reviewed and approved by the Institutional Animal Care and Use Committee of A*STAR Biomedical Sciences Institutes. All mice were on a C57BL/6 background. Serum and plasma levels of EPAS1 were quantified by ELISA.

### Shear Stress Maps

The lumen geometry of the mouse aorta was derived previously.^24^ The unsteady Navier-Stokes equations were solved numerically using the finite volume method using the specialized in-house hemodynamics solver haemoFOAM (https://github.com/TS-CUBED/haemoFoam).

### Antibodies

Primary and secondary antibodies and the concentrations used are documented in Table S2 and in the Major Resources Table.

### Endothelial RNA Extraction

RNA was extracted from aortic EC using the Qiazol flushing method as described.^25^

### Atherosclerosis Plaque Analysis

Aortic root sections were stained with Mayer’s hematoxylin and eosin (plaque area), Picrosirius Red (collagen), elastin van Gieson (elastin), and anti-MAC3 (macrophages), followed by microscopy and quantitation using ImageJ software.

### Immunofluorescent Staining of Murine Endothelium

The expression levels of specific proteins were assessed in EC at regions of the inner curvature exposed to low oscillatory shear stress (LOSS) and outer curvature exposed to physiological high shear stress (HSS) of murine aortae by en face staining and confocal microscopy. BODIPY 493/503 was used to delineate lipid droplets. EC were identified by costaining using anti-CD31 or VE-cadherin (CDH5) antibodies. Nuclei were identified using TO-PRO-3. Details of all primary and secondary antibodies used in this article are provided in Table S2 and in the supplementary Major Resource Table.

### Single-Cell RNA Sequencing

Single-cell RNA sequencing (RNAseq) libraries were generated from age- and sex-matched *Epas1*^*EC-KO*^ and *Epas1*^*EC-WT*^ mice using the sorting and robot-assisted transcriptome sequencing protocol as described previously.^26^

### Culture of EC and Exposure to WSS

Porcine aortic EC (PAEC) from multiple donors were seeded onto gelatin-coated Ibidi µ-Slides I^0.4^ before exposure to HSS (13 dyn/cm^2^), high shear stress (4 dyn/cm^2^), or low oscillatory wall shear stress (WSS).^27^ Alternatively, PAECs were cultured in a 6-well plate and exposed to shear stress using the orbital system as described.^9^

### Gene Silencing

PAEC cultures were transfected with a pool of small hairpin RNAseq targeting porcine *EPAS1* or using the Dharmacon SMARTvector Lentiviral system. A nontargeting lentiviral-small hairpin RNAseq was used as a control (V16060303, Dharmacon).

### Real-Time PCR

Quantitative RNA PCR was performed using gene-specific primers (Table S1). Expression values were normalized against the housekeeping genes *B2M* (pig) and Hprt (human, mouse).

### XF24 Metabolic Fuel Flex Assay

Metabolism was analyzed in cultured PAEC by performing mitofuel and mitostress assays. Extracellular acidification rate was measured basally after glucose starvation and after addition of oligomycin to assess glycolytic capacity.

### Immunoblotting

Total cell lysates were isolated using lysis buffer (containing 2% sodium dodecyl sulfate, 10% Glycerol, and 5% β-mercaptoethanol) and immunoblotted using primary antibodies. HRP-conjugated secondary antibodies and chemiluminescent detection were performed using ECL Prime.

### Clinical Adipose and Serum Samples

Human subcutaneous adipose tissues (n=29) were collected from obese (body mass index >30) and nonobese (body mass index <30) individuals undergoing abdominal surgery before EC isolation as described.^28^ Deidentified demographic data of the patients were retrieved using the Generic Clinical Research Database at the Medical College of Wisconsin. All procedures were approved by the Institutional Review Board of Medical College of Wisconsin and Froedtert Hospital. Peripheral blood samples were taken from healthy nonobese subjects (n=14) and from severe obese subjects with body mass index >38 (n=15) with approval from the local Research and Ethic Committees in Zurich, Switzerland (Ethic Nr.KEK-ZH_Nr.2013-0389). All patients gave written informed consent. Studies were performed according to the principles of the Declaration of Helsinki.

### Statistical Analysis

Statistical analysis was performed using GraphPad Prism software. Data are presented as mean±SEM. Representative images were chosen to represent the average results achieved within the experiment. Data were tested for normality of distribution using the Shapiro-Wilk normality test. Data that followed a parametric distribution were analyzed using ANOVA or *t* tests. In case of deviation from normality or in presence of a small sample size (N<6), a nonparametric Mann-Whitney *U* test, Kruskal-Wallis, or aligned ranked transformed ANOVA was used. Multiple comparisons in ANOVA were corrected using the Sìdak multiple comparison test. *P* values <0.05 were considered significant. The *P* value and the test performed are indicated in the figure and figure legend, respectively.

## RESULTS

### EPAS1 Is Enriched at an Atheroprone Site Exposed to LOSS

**Figure 1.**
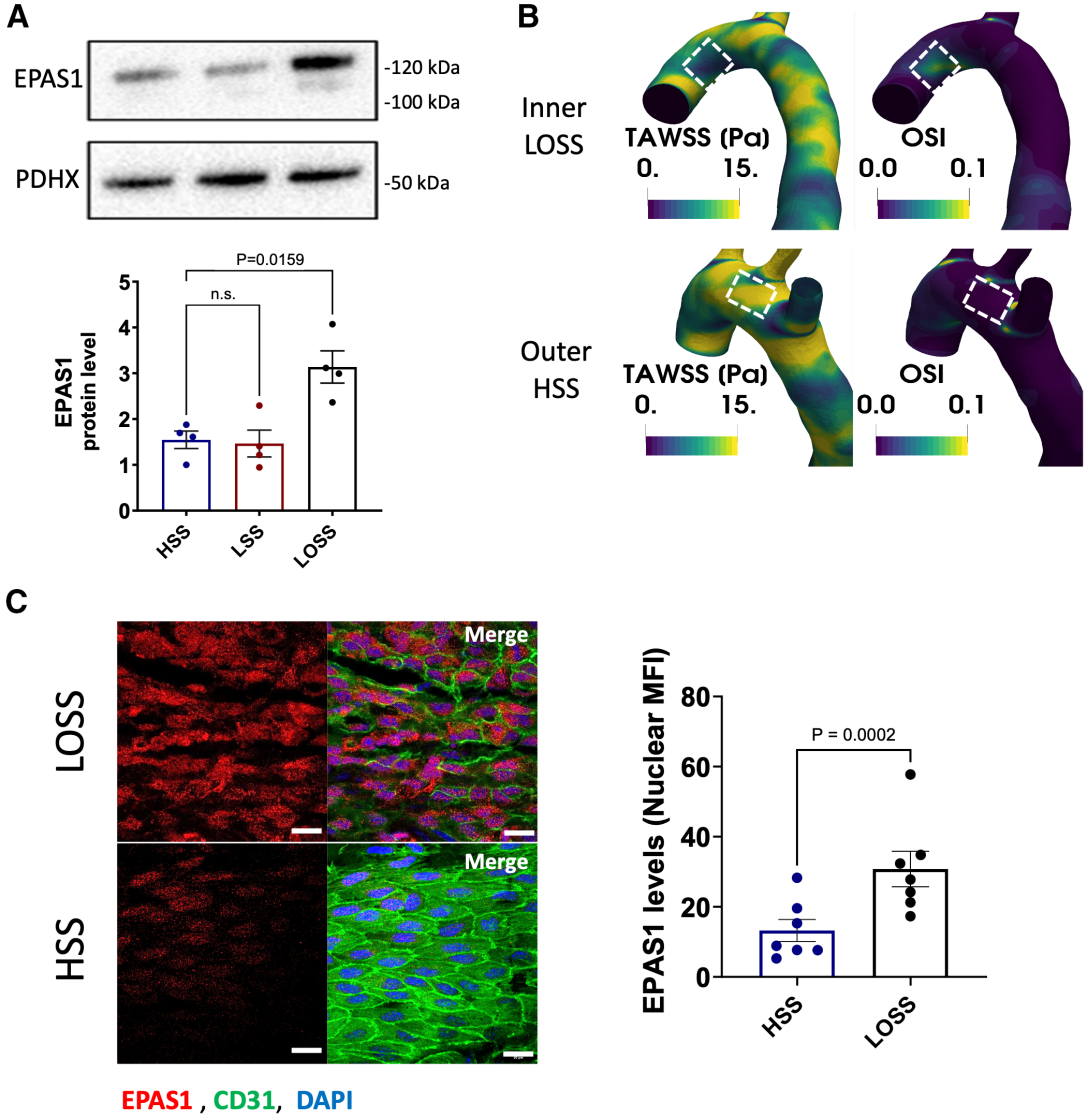
**EPAS1 is enriched at an atheroprone site exposed to low and oscillatory shear stress. A**, PAEC were seeded on μ-slides and cultured under high shear stress (HSS), low shear stress (LSS), or low oscillatory shear stress (LOSS) for 72 hours using the Ibidi system. Protein levels of EPAS1 were quantified by immunoblotting. Representative images and mean values normalized to the level of PDHX (pyruvate dehydrogenase complex component X) (n=4) are shown. Differences between means were analyzed using a Kruskal-Wallis test. **B**, Time-averaged wall shear stress (WSS) (TAWSS) and oscillatory shear index (OSI) were mapped onto the geometry of the murine aortic arch. Representative images are shown and regions exposed to LOSS (inner) or HSS (outer) are marked. **C**, Aortic arches were isolated from C57BL/6J mice aged 6 to 8 weeks, and en face immunostaining was performed using anti-EPAS1 antibodies (red). Endothelium was costained (EC; green) and nuclei detected using DAPI (4′,6-diamidino-2-phenylindole; blue). Scale bar: 20 µm. EPAS1 levels were quantified at LOSS and HSS regions (n=7). Each data point represents an animal. Differences between means were analyzed using a paired *t* test.

To analyze whether EPAS1 is responsive to flow, we exposed PAEC to varying shear stress conditions using a parallel plate system. Immunoblotting revealed that EPAS1 expression was increased in PAEC exposed to LOSS (4 dynes/cm^2^; bidirectional with 1 Hz frequency) compared with high shear stress (4 dynes/cm^2^) or unidirectional HSS (13 dynes/cm^2^; Figure 1A). We then measured EPAS1 levels in EC of the mouse aortic arch, which is known to generate disturbed flow associated with atherosclerosis initiation. CT-angiography coupled to computational fluid dynamics showed that the inner curvature was exposed to relatively low time-averaged WSS magnitude and more oscillations in direction (ie, LOSS) compared with the outer curvature (Figure 1B), consistent with prior analyses.^24,29^ In agreement with our in vitro findings, en face immunofluorescent staining of the murine aorta revealed that EPAS1 was significantly enriched at the LOSS region (inner curvature) compared with HSS regions (Figure 1C). A proportion of EPAS1 at the LOSS site localized to the nucleus, suggesting that it is active (Figure 1C). Thus, EPAS1 is enhanced by LOSS in vitro, and it is enriched at an atheroprone region of the murine aorta exposed to LOSS.

Since HIF1A is also enriched in atheroprone endothelium, we examined the interrelationship of HIF1A and EPAS1. Costaining demonstrated that 7% of EC at the LOSS region express EPAS1 in the absence of HIF1A (EPAS1+ HIF1A−), whereas the majority of EC (92%) are positive for EPAS1 and HIF1A (EPAS1+ HIF1A+), whereas 0% cells express HIF1A in the absence of EPAS1 (EPAS1− HIF1A+). Only 0.66% of cells were negative for EPAS1 and HIF1A (EPAS1− HIF1A−; Figure S1).

### EPAS1 Is Regulated by Metabolic Status in Obese Mice

**Figure 2.**
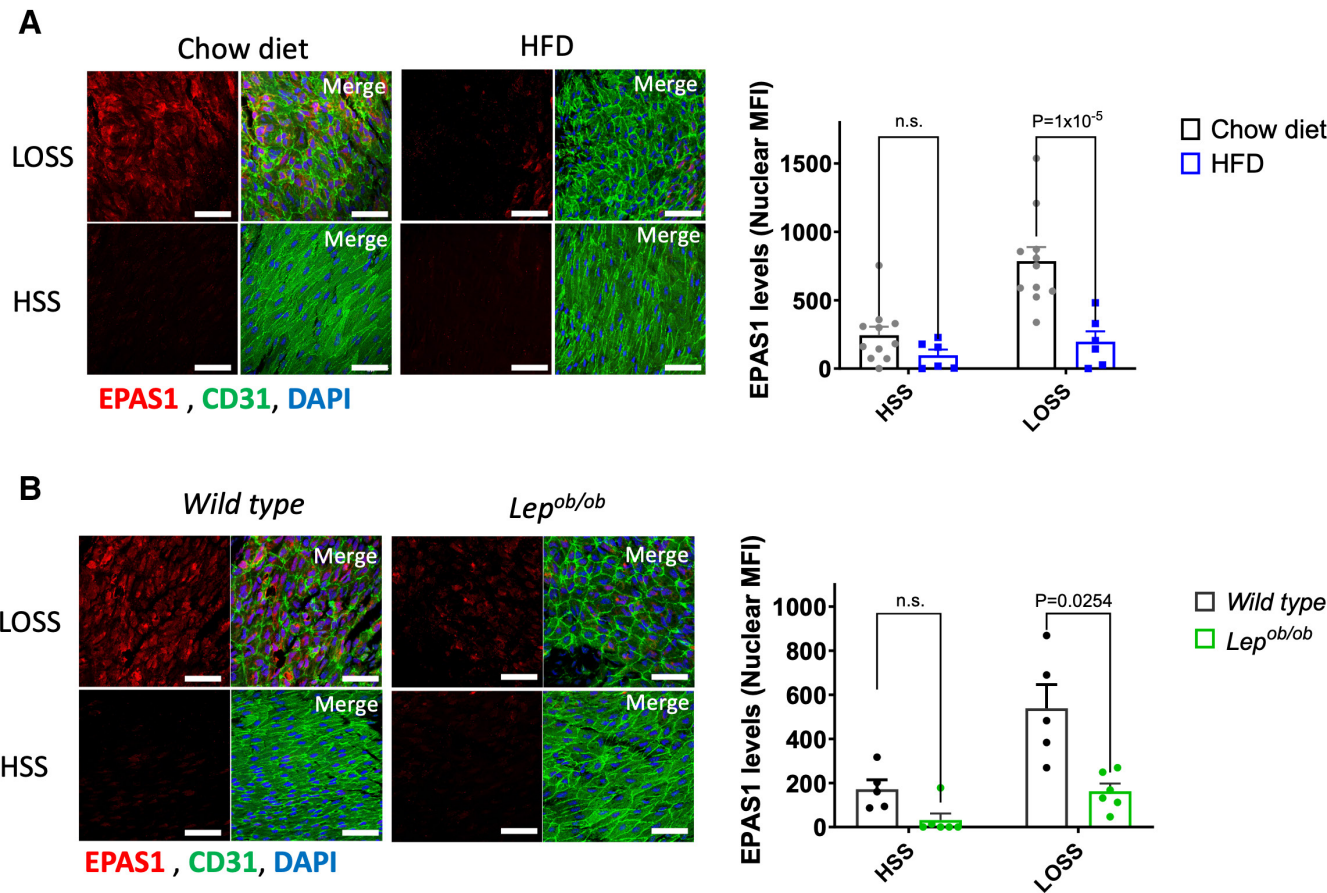
**High-fat feeding and obesity reduced EPAS1 levels at atheroprone aortic endothelium. A**, C57BL/6N mice aged 5 weeks were exposed to high fat diet (HFD) (n=6) or to standard chow (n=11) for 25 weeks. **B**, *Lep*^*ob/ob*^ mice (n=6) and littermate controls (wild-type, n=5) aged 22 weeks were analyzed. **A** and **B**), Aortic endothelial cells were stained en face using anti-EPAS1 antibodies (red). Endothelium was costained (CD31; green) and nuclei detected using DAPI (4′,6-diamidino-2-phenylindole; blue). Scale bar: 50 µm. EPAS1 fluorescence levels were quantified at low oscillatory shear stress (LOSS) and high shear stress (HSS) regions, and mean±SDs are presented. Each data point represents an animal. Differences between means were analyzed using a 2-way ANOVA with Sìdak’s multiple comparisons test in A, while B was analyzed using an aligned ranked transform ANOVA test. MFI indicates mean fluorescence intensity.

Dysregulation of the HIF pathway has been associated with the development of several cardiovascular risk factors, including obesity and type 2 diabetes. Nevertheless, the possible effect of these risk factors on endothelial HIF expression has not been explored. To assess the effect of obesity on aortic endothelial EPAS1 levels, we exposed WT mice to HFD or a normal chow diet for 22 weeks. HFD mice were significantly heavier than chow diet mice (Figure S2A). Weight gain was associated with hyperglycemia (Figure S2B), increased triglyceride levels (Figure S2C), and raised total cholesterol levels (Figure S2D). We measured plasma HDL (high-density lipoprotein) cholesterol, which showed no statistically significant differences between chow and HFD mice, while the LDL/VLDL (low-density lipoprotein/very-low-density lipoprotein) ratio was significantly increased in HFD mice (Figure S1E). In addition, HFD did not elicit significant changes in the mouse systolic blood pressure despite elevated diastolic pressure (Figure S1F). En face staining of the aortic arch revealed a striking reduction in EPAS1 levels at the LOSS region in mice exposed to HFD compared with chow-fed control mice (Figure 2A). Thus, HFD feeding associated with obesity reduces EPAS1 expression at a LOSS atheroprone region.

To confirm the influence of obesity on endothelial EPAS1 levels, we analyzed an alternative model of obesity using mice with homozygous deficiency in leptin (*Lep*^*ob/ob*^), which gained weight more rapidly than littermate controls (Figure S3A). *Lep*^*ob/ob*^ mice were characterized by several metabolic changes, including transient hyperglycemia, raised within 14 weeks, which normalized at 22 weeks (Figure S3B). *Lep*^*ob/ob*^ mice exhibited increased levels of plasma TG (Figure S3C), increased total cholesterol (Figure S3D), and increased LDL/VLDL ratio with no statistically significant changes in HDL cholesterol levels (Figure S3E). It was observed that EPAS1 levels at LOSS regions were strikingly reduced in *Lep*^*ob/ob*^ mice compared with WT controls (Figure 2B). Therefore, obesity induced through either exposure to a HFD or genetic loss of leptin led to a substantial reduction in EPAS1 levels in aortic endothelium.

**Figure 3.**
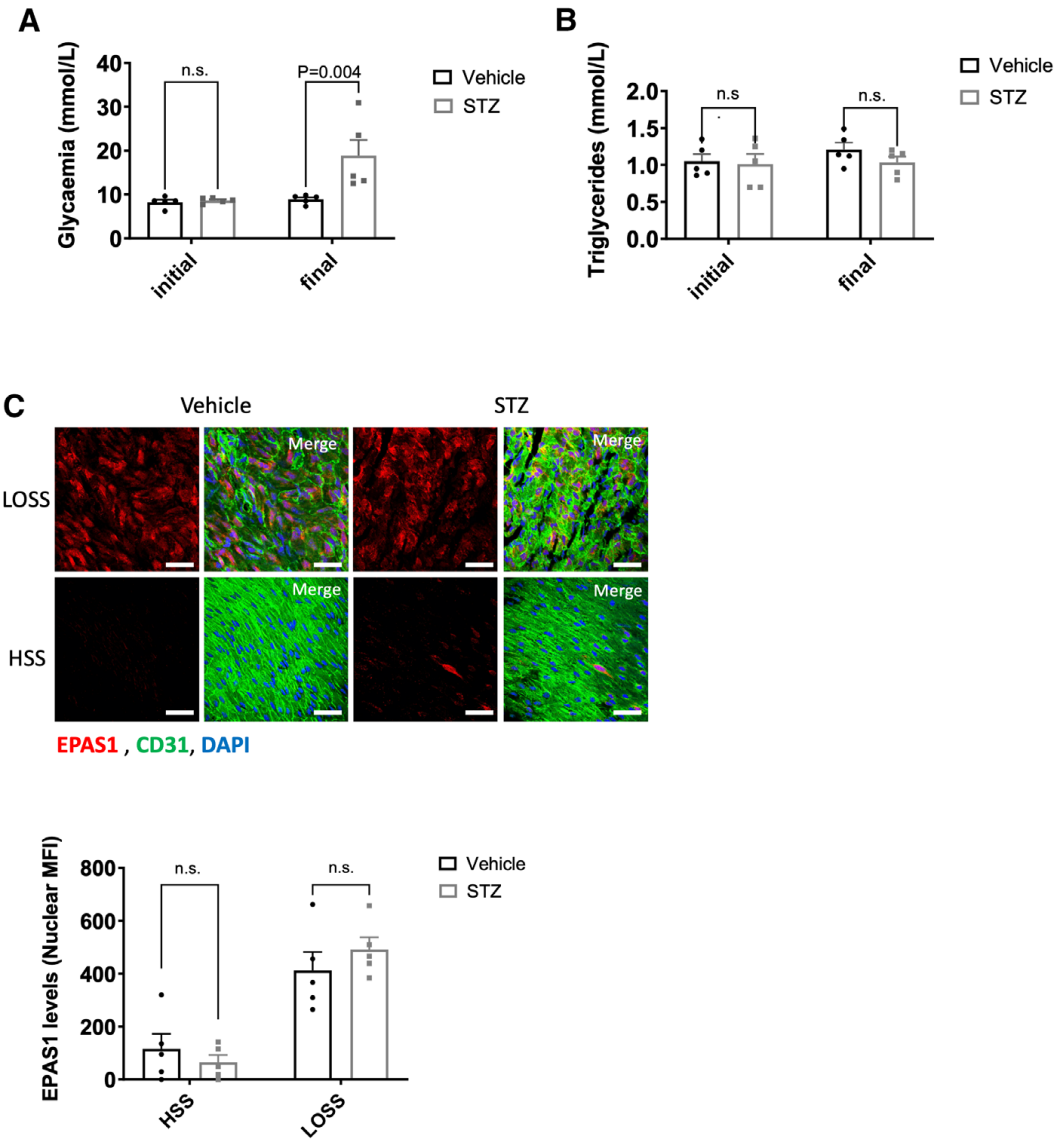
**Hyperglycemia had no statistically significant effect on EPAS1.** Mice (C57BL/6J) aged 20 weeks were treated with streptozotocin (STZ) or vehicle control (initial) and analyzed 2 weeks later (final), n=5 animals per group. Glycemia (**A**) and plasma triglycerides (**B**) were measured. **C**, Endothelial cells were stained en face using anti-EPAS1 antibodies (red). Endothelium was costained (CD31; green) and nuclei detected using DAPI (4′,6-diamidino-2-phenylindole; blue). Scale bar: 50 µm. EPAS1 fluorescence levels were quantified at low oscillatory shear stress (LOSS) and high shear stress (HSS) regions, and mean±SDs are presented. Each data point represents an animal. Differences between means were analyzed using an aligned ranked transform ANOVA test (**B** and **C**). MFI indicates mean fluorescence intensity.

We next sought to define the mechanism of EPAS1 reduction in obesity. The potential contribution of hypertension was eliminated because *Lep*^*ob/ob*^ mice exhibited a significant decrease in both systolic and diastolic blood pressure compared with controls (Figure S3F). Despite the severe obesity status, this phenomenon was predictable and previously associated with the *Lep*^*ob/ob*^ phenotype.^30^ We, therefore, focused on the effects of metabolic alterations in obesity on EPAS1 expression. The potential contribution of hyperglycemia was analyzed by treating mice with streptozotocin, which induces diabetes associated with endothelial dysfunction and uncoupling of endothelial nitric oxide synthase.^31^ Streptozotocin induced sustained hyperglycemia in chow-fed mice (Figure 3A) and had no statistically significant effect on TG levels (Figure 3B). En face staining revealed that EPAS1 expression at the LOSS region of the aorta was unaltered in streptozotocin-treated mice (Figure 3C), indicating that hyperglycemia is insufficient to suppress EPAS1. To corroborate this finding, we investigated the levels of EPAS1 in the aorta of *Lep*^*ob/ob*^ mice during their hyperglycemic phase at 14 weeks. Hyperglycemic *Lep*^*ob/ob*^ mice exhibited a relatively modest reduction in EPAS1 that did not reach statistical significance (Figure S4), confirming that EPAS1 suppression in obesity can be uncoupled from hyperglycemia.

### Sulforaphane Rescued EPAS1 in Obese Mice Coupled to TG Lowering

**Figure 4.**
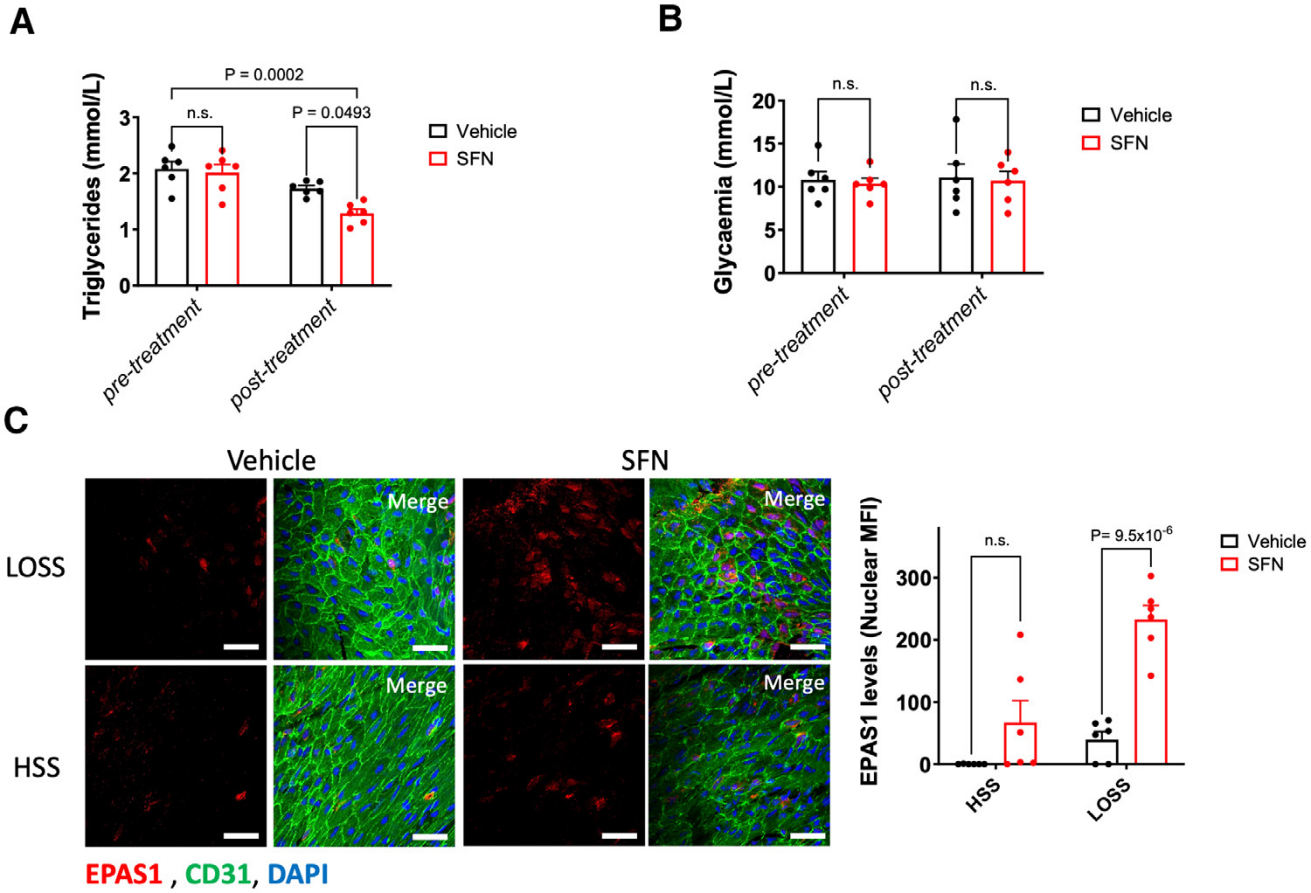
**Sulforaphane rescues EPAS1 in obese mice.** C57BL/6N mice aged 5 weeks were exposed to high fat diet (HFD) for 25 weeks (pretreatment). They were then treated with sulforaphane (SFN; daily IP injections 5 mg/kg for 3 days) or with vehicle for 3 days, with both groups receiving a HFD for that period, n=6 animals per group. Plasma triglycerides (**A**) and glycemia (**B**) were measured pretreatment and in SFN-treated and vehicle-treated groups. **C**, Aortic endothelial cells (EC) were stained en face using anti-EPAS1 antibodies (red), and fluorescence was quantified at low oscillatory shear stress (LOSS) and high shear stress (HSS) regions in SFN-treated and control groups. Endothelium was costained (EC; green) and nuclei detected using DAPI (4′,6-diamidino-2-phenylindole; blue). Each data point represents an animal. Differences between means were analyzed using a 2-way ANOVA using a Sìdak’s multiple comparison test (**A** and **B**) or an aligned ranked transform ANOVA test (**C**).

The potential role of hyperlipidemia in EPAS1 suppression was analyzed by treatment of obese mice with sulforaphane, a compound from broccoli and other cruciferous vegetables that is known to modify lipid profiles^32^ and protect endothelium.^33^ Sulforaphane treatment significantly reduced plasma levels of TGs in obese mice exposed to the HFD (Figure 4A), whereas glycemia (Figure 4B), body weight (Figure S5A), and plasma levels of total cholesterol (Figure S5B), HDL, and LDL/VLDL (Figure S5C) were not significantly altered. Sulforaphane treatment of obese mice restored the expression of EPAS1 at the LOSS region and modestly raised EPAS1 at the HSS region (Figure 4C).

**Figure 5.**
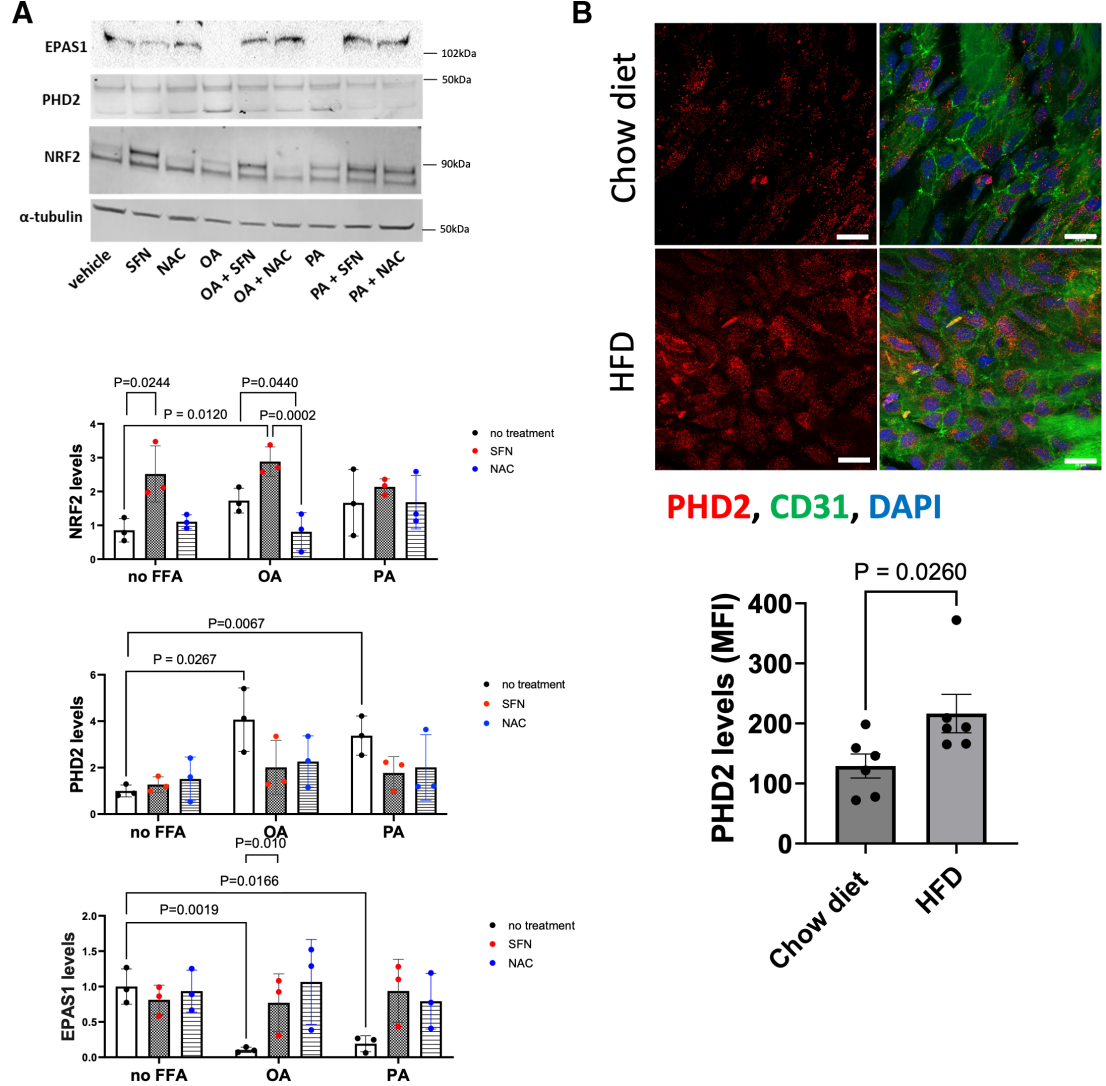
**Free fatty acids suppress EPAS1 via the destabilizing enzyme PHD2. A**, Porcine aortic endothelial cells were exposed to low oscillatory shear stress (LOSS) for 72 hours using the orbital system in the presence or absence of OA (0.25 mmol/L) or PA (0.25 mmol/L). Some cultures were treated with sulforaphane (SFN; 10 μM) or NAC (n-acetyl cysteine) (1 mM) either alone or together with OA or PA for 24 hours. Protein levels of EPAS1, PHD2, and NRF2 were quantified by immunoblotting. Representative images and mean values normalized to the level of α-tubulin (n=3) are shown. Differences between means were analyzed using an aligned ranked transform ANOVA test. **B**, C57BL/6J mice aged 10 weeks were exposed to high fat diet (HFD) or control chow diet for 10 weeks, n=6 per group. Aortic arch endothelial cells (EC) were stained en face using anti-PHD2 antibodies (red), and fluorescence was quantified at the LOSS region. Endothelium was costained (EC; green) and nuclei detected using DAPI (4′,6-diamidino-2-phenylindole; blue). Scale bar: 20 µm. Each data point represents an animal. Differences between means were analyzed using a nonparametric *t* test. MFI indicates mean fluorescence intensity.

The observation that EPAS1 rescue correlated with reduced plasma TG in SFN-treated obese mice led us to hypothesize that TGs may be involved in EPAS1 suppression in obesity. This was tested by exposing PAEC cultured under LOSS to free fatty acids (FFAs), which are produced from the local hydrolysis of TG. Immunoblotting revealed that EPAS1 levels were significantly reduced by exposure to PA or OA (Figure 5A; compare lanes 1 with 4 and lanes 1 with 7). To investigate the underlying mechanism, we performed immunoblotting of PHD2 and PHD3 proteins that target EPAS1 for proteasomal degradation.^20^ PHD2 levels were significantly increased by PA or OA (Figure 5A), suggesting that FFAs may reduce EPAS1 via PHD2 induction. The observation was validated by en face staining of aortas, which demonstrated that PHD2 expression at the LOSS region was enhanced in obese mice fed HFD compared with nonobese mice (Figure 5B). Immunoblotting for PHD3 was inconclusive since commercial antibodies did not generate a specific signal (data not shown). Sulforaphane can exert direct effects on EC via activation of NRF2 and suppression of reactive oxygen species).^33,34^ The potential involvement of this pathway in EPAS1 rescue was investigated by treating PAEC with palmitic acid (PA) or palmitic acid in the presence or absence of sulforaphane or the antioxidant NAC (n-acetyl cysteine). To validate the effects of these compounds on PAECs, it was demonstrated that sulforaphane and NAC can suppress reactive oxygen species (Figure S6), whereas SFN alone or in the presence of OA had the capacity to enhance NRF2 (Figure 5A). Sulforaphane and NAC increased mean EPAS1 levels in PAEC co-treated with PA or OA, albeit not reaching statistical significance (Figure 5A; compare lanes 4, 5, 6 and lanes 7, 8, 9); therefore, we cannot exclude the possibility that SFN rescues FFA-mediated EPAS1 suppression by reducing reactive oxygen species. Moreover, the mechanism may involve suppression of PHD2, which was reduced by SFN and NAC, albeit not reaching statistical significance (Figure 5A). Thus, EPAS1 rescue in sulforaphane-treated obese mice may potentially involve direct effects of SFN on endothelium. Collectively, these observations suggest that EPAS1 reduction in obesity may result from hypertriglyceridemia-FFA metabolism coupled to localized alterations in cellular oxidative stress leading to the induction of endothelial PHD2.

### Microvascular EPAS1 and Circulating Serum EPAS1 Are Reduced in Obese Mice

We assessed whether EPAS1 suppression in obesity could be observed in blood serum and peripheral microvasculature. Immunofluorescence staining revealed that EPAS1 was expressed in the dermal microvasculature of nonobese mice (Figure S7A), and ELISA revealed the presence of EPAS1 in the blood serum of nonobese mice (Figure S7B). Notably, both microvascular EPAS1 (Figure S7A) and serum EPAS1 (Figure S7B) were reduced in obesity induced by HFD. PHD2 exhibited the opposite pattern of expression since it was enhanced in the microvascular EC of obese mice compared with controls (Figure S7A). To analyze whether endothelium contributes to circulating EPAS1, we deleted endothelial *Epas1* by crossing *Cdh5*^*CreERT2/+*^ and *Epas1*^*fl/fl*^ mice to generate *Epas1*^*EC-KO*^ and control *Epas1*^*EC-WT*^ mice. Tamoxifen treatment of *Epas1*^*EC-KO*^ and control *Epas1*^*EC-WT*^ mice induced deletion of *Epas1*, which was validated by quantitative real-time PCR of endothelial RNA (Figure S8A) and by en face staining (Figure S8B). *Epas1*^*EC-KO*^ and *Epas1*^*EC-WT*^ mice were exposed to an HFD for 8 weeks. ELISA revealed that serum EPAS1 levels were significantly reduced in *Epas1*^*EC-KO*^ compared with *Epas1*^*EC-WT*^ mice (Figure S7C), indicating that endothelium contributes to the pool of circulating EPAS1. Overall, obesity in mice led to reduced EPAS1 in the microvascular endothelium coupled to reduced serum EPAS1.

### Reduced Endothelial EPAS1 in Clinical Obesity

The clinical relevance of our findings was assessed by comparing the PHD-EPAS1 pathway in obese versus nonobese individuals. Since sampling of arterial EC was not feasible, we investigated the EPAS1 pathway in blood serum and microvasculature as a surrogate. Males and females were analyzed in the obese (53.33% female) and nonobese (50% female) cohorts. There were no significant differences in average age between obese and nonobese cohorts (34.67 versus 31.21; *P*=0.30). The obese cohort was associated with increased rates of hypertension (33.3%) and diabetes/prediabetes (26.7%) compared with previously analyzed age-matched nonobese populations^35^ (Table S3). Dyslipidemia (60%) was elevated in the obese cohort with increased levels of TG, total cholesterol, and LDL compared with nonobese populations (Table S3).

**Figure 6.**
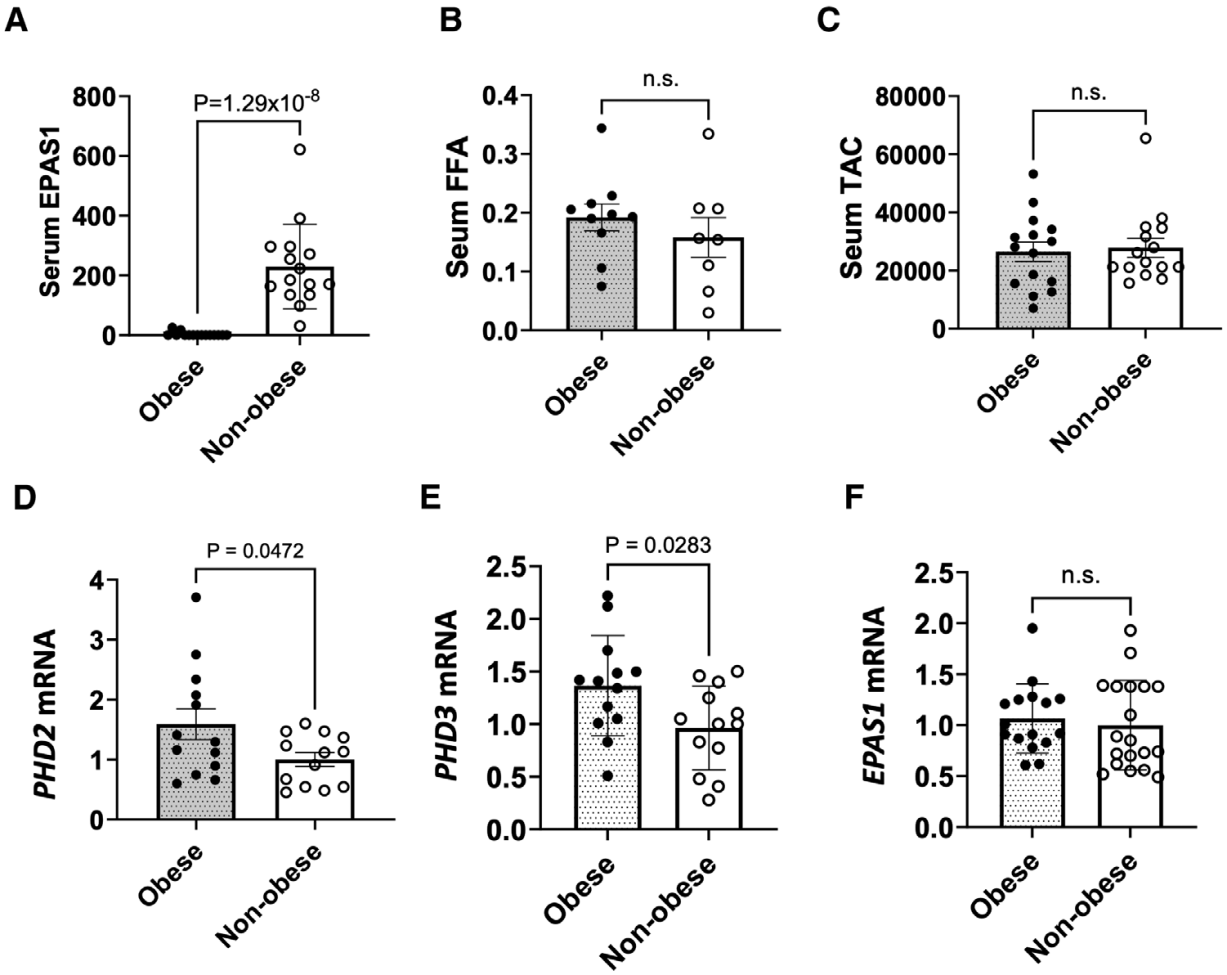
**Alteration of the prolyl hydroxylase domain (PHD)-EPAS1 pathway in clinical obesity. A** through **C**, Obese (body mass index >38; n=15) and nonobese subjects (n=15) were analyzed. Serum levels of EPAS1 (**A**), free fatty acids (FFAs) were measured (n=10) obese and (n=8) nonobese controls (**B**), and total antioxidant capacity (TAC) was measured in obese and nonobese subjects, n=15 each group. Mean values are shown with SEs. **D** through **F**, Endothelial cells (EC) were isolated from adipose samples from obese (n=12) and nonobese (n=13) subjects. Levels of *PHD2* (**D**), *PHD3* (**E**), and *EPAS1* (**F**) transcripts were quantified by quantitative real-time polymerase chain reaction. Each data point represents a human subject. Differences between means were analyzed using a Mann-Whitney *U* test (**A**, **C**, and **F**) or a parametric *t* test (**B**, **D**, and **E**).

ELISA revealed that EPAS1 was detectable in serum samples from all nonobese controls studied but was either absent or significantly reduced in obese individuals (Figure 6A). We investigated whether reduced serum EPAS1 in clinical obesity was associated with altered circulating lipids and ROS. Levels of FFAs were modestly increased in obese compared with nonobese individuals; however, this difference did not reach statistical significance (Figure 6B). Total antioxidant capacity of serum was similar between obese and nonobese groups (Figure 6C). This pathway was further interrogated by quantifying *EPAS1*, *PHD2*, and *PHD3* transcripts in microvascular EC isolated from subcutaneous adipose of obese patients and nonobese controls. Quantitative real-time PCR revealed that *PHD2* (Figure 6D) and *PHD3* (Figure 6E) levels were significantly increased in obese individuals compared with controls, whereas *EPAS1* mRNA expression was unaltered (Figure 6F). Overall, these data indicate that EPAS1 is suppressed in obese individuals associated with dyslipidemia and elevated *PHD2* and *PHD3* expression in EC, an observation that is consistent with our observations in murine models of obesity.

### *Epas1* Protects the Endothelium Against Atherosclerosis by Regulating Metabolic and Proliferative Pathways

**Figure 7.**
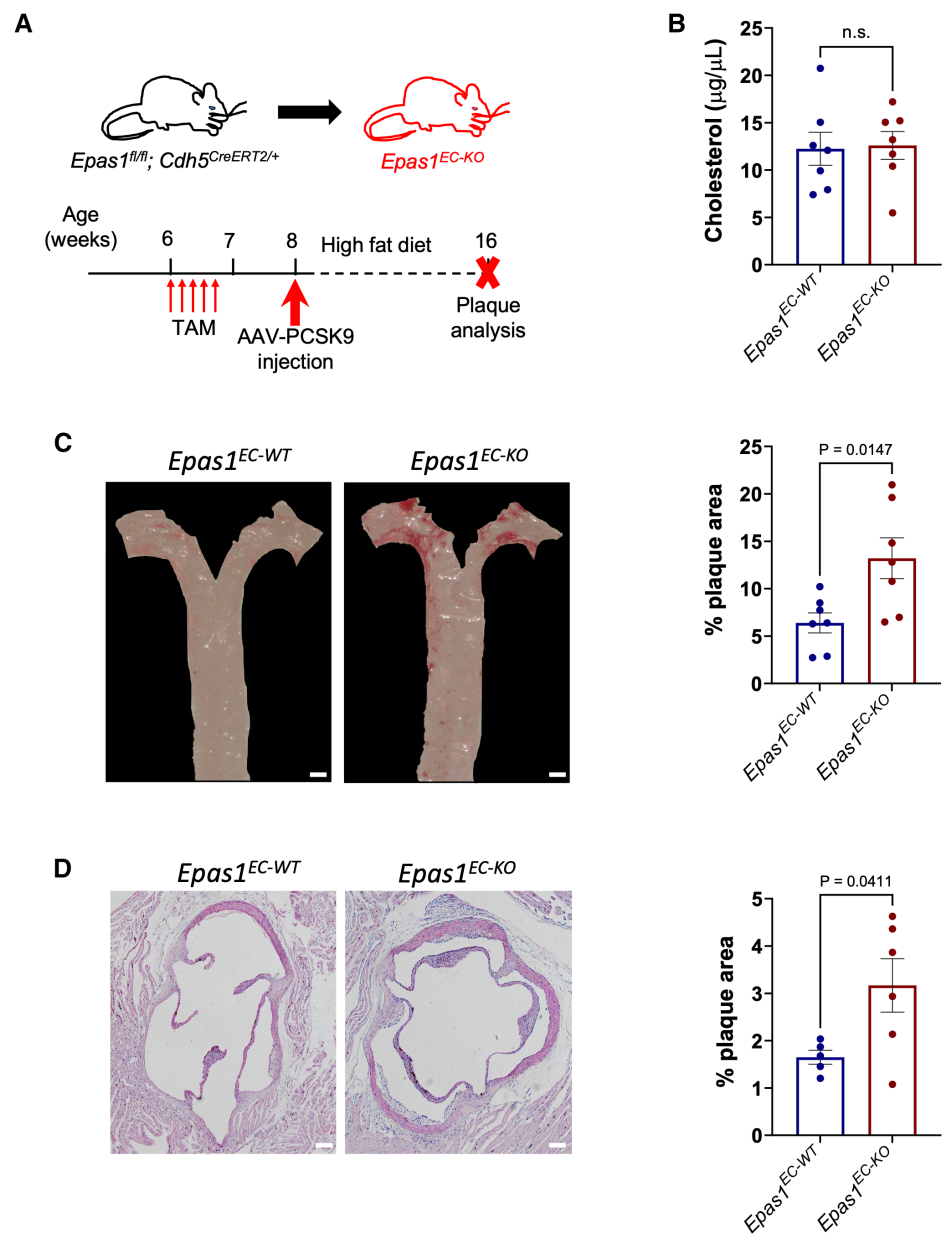
**Endothelial *Epas1* protects against atherosclerosis. A**, Timeline of *Epas1* deletion in a model of hypercholesterolemia. *Epas1*^*EC-KO*^ mice aged 6 weeks and *Epas1*^*EC-WT*^ mice received 5 intraperitoneal injections of tamoxifen and 1 injection of PCSK9-adeno-associated virus at specified time points. After 8 weeks fed with a high-fat diet, the mice were culled and plaque area quantified. **B**, Plasma cholesterol levels were measured in *Epas1*^*EC-KO*^ mice and *Epas1*^*EC-WT*^ controls, n=7 per group. **C**, Quantification of plaque burden in the aorta was determined by calculating the percentage of aortic surface area covered by plaque for *Epas1*^*EC-KO*^ mice and *Epas1*^*EC-WT*^ controls, n=7 per group. **D**, Quantification of plaque burden in the aortic roots of *Epas1*^*EC-KO*^ mice (n=6) and *Epas1*^*EC-WT*^ controls (n=5). Scale bar: 200 µm. Each data point represents 1 mouse, and mean±SEMs are shown. Differences between means were analyzed using an unpaired *t test* (**A** through **C**) or a Mann-Whitney *U* test (**D**).

To analyze the role of *Epas1* in atherosclerosis, we generated *Epas1*^*EC-KO*^ and control *Epas1*^*EC-WT*^ mice, which were treated with AAV-PCSK9 and exposed to a HFD for 8 weeks to generate hypercholesterolemia (Figure 7A). Endothelial deletion of *Epas1* had no effect on plasma cholesterol levels (Figure 7B). However, atherosclerotic plaques in the aorta were significantly larger in *Epas1*^*EC-KO*^ mice compared with controls (Figure 7C). Plaque formation was also measured at the aortic root, confirming the presence of larger plaques in *Epas1*^*EC-KO*^ mice compared with *Epas1*^*EC-WT*^ controls (Figure 7D). The potential influence of *Epas1* on plaque composition was next assessed. Endothelial deletion did not influence macrophage accumulation (Figure S9A), elastin breaks (Figure S9B), or intraplaque necrosis (Figure S9C). Collagen was not detected in plaques of either group (Figure S9D). These data show that endothelial *EPAS1* protects against atherosclerosis but had negligible effects on plaque composition in this model.

To gain initial insight into the function of endothelial EPAS1, we performed single-cell RNAseq from the aortas of *Epas1*^*EC-KO*^ and control (*Epas1*^*EC-WT*^) mice. ECs were isolated by enzymatic digestion and CD31^+^ CD45^−^ TO-PRO-3^−^ EC and purified by fluorescence-activated cell sorter and processed for single-cell RNAseq (Figure S10A). Our sort-sequencing analysis identified 6 distinct clusters (Figure S10B). Of these, clusters 0 to 4 were significantly enriched for multiple EC markers, whereas cluster 5 was significantly enriched for VSMC-like features and was subsequently removed from the analysis of EC heterogeneity (Figure S10C).

Considering the potential influence of *Epas1* on EC heterogeneity, cluster 0 was enriched with cells from *Epas1*^*EC-WT*^ mice (34% of the *Epas1*^*EC-WT*^ cell population versus 19% of the *Epas1*^*EC-KO*^ population; Figure S10D and S10E). By contrast, cluster 1 was enriched with cells from *Epas1*^*EC-KO*^ mice (23% of the *Epas1*^*EC-WT*^ cell population versus 34% of the *Epas1*^*EC-KO*^ population), and cluster 4 was enriched with cells from *Epas1*^*EC-KO*^ mice (3% of the *Epas1*^*EC-WT*^ cell population versus 8% of the *Epas1*^*EC-KO*^ population; Figure S10E). These data suggest that *Epas1* influences EC heterogeneity.

Functional annotation revealed that *Epas1*-regulated clusters were enriched for multiple diverse gene ontology terms, including intracellular signaling pathways, transcription/RNA processing, cell proliferation, and metabolism (eg, mitochondrial function and lipid transport; Figure S10F). Focusing on proliferation and metabolism, we observed that enrichment of the terms “DNA replication,” “positive regulator of endothelial proliferation,” and “positive regulation glycolytic process” varied among clusters 0, 1, and 4 (Figure S11A). Further functional annotation of cells from *Epas1*^*EC-KO*^ versus *Epas1*^*EC-WT*^ mice revealed multiple enriched gene ontology terms, including those linked to actin, Golgi apparatus and membrane (cluster 0), translation, transport, oxidative phosphorylation, mitochondrion (cluster 1) and development, MAP kinase signaling, and glycoprotein (cluster 4; Figure S11B). Overall, these data lead us to hypothesize that EPAS1 may be involved in metabolic regulation among other functions in arterial endothelium.

### EPAS1 Promotes Fatty Acid β-Oxidation and Proliferation Under Atheroprone Shear Stress

**Figure 8.**
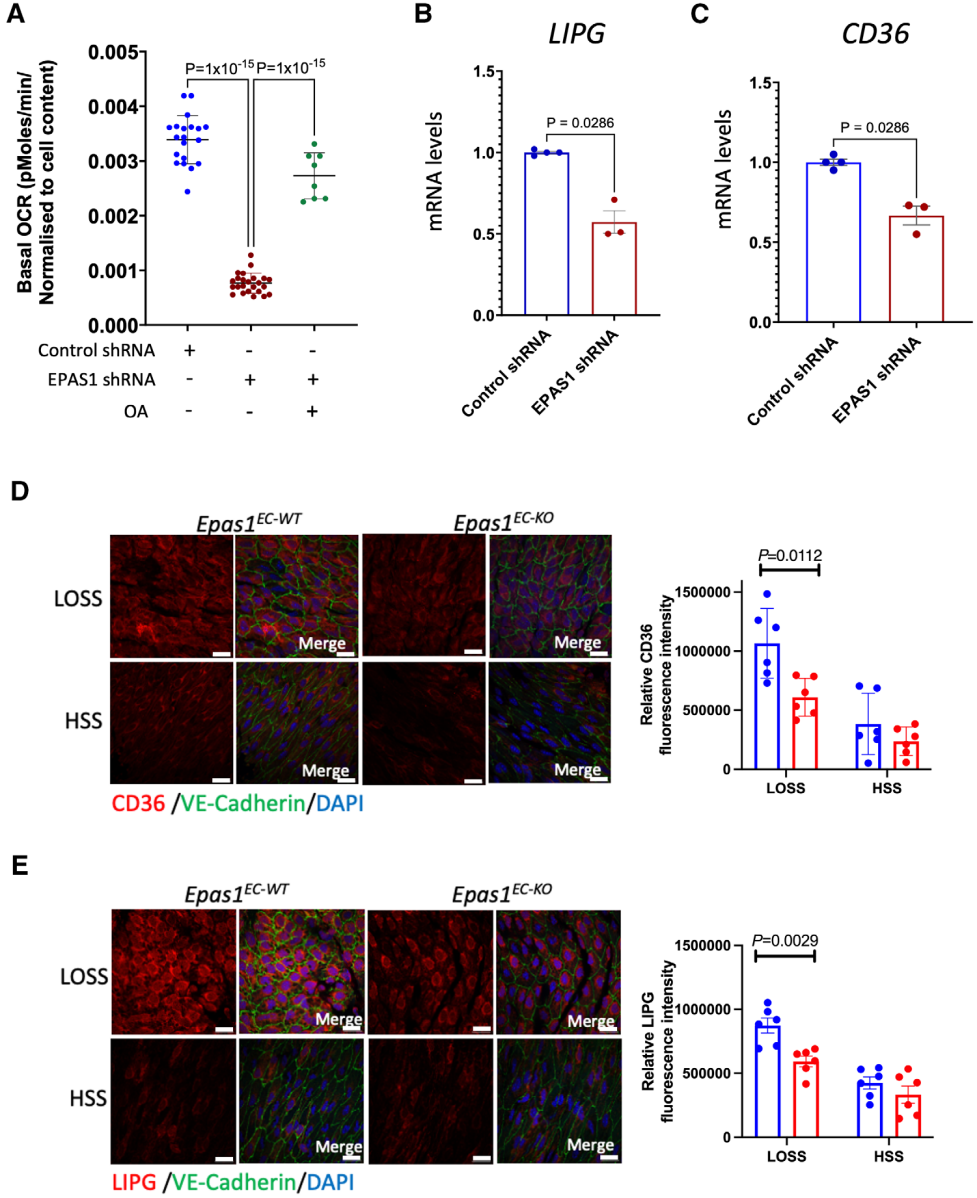
**Endothelial EPAS1 controls fatty acid metabolism via LIPG (endothelial type lipase G) and CD36 (cluster of differentiation 36) expression. A** through **C**, PAEC were treated with small hairpin RNA (shRNA) targeting *EPAS1* (n=3) or with scrambled control (n=4) and exposed to low oscillatory shear stress (LOSS) for 72 hours using the orbital system. **A**, Some cultures were exposed to exogenous OA (0.25 mmol/L). Basal oxygen consumption rates (OCR) were measured. Each dot represents a technical replicate from 3 PAEC donors: control shRNA (n=20), EPAS1 shRNA KD (n=24), and EPAS1 KD treated with OA (n=8). Average values are shown ±SEs. Expression of *CD36* (**B**) or *LIPG* (**C**) was quantified by quantitative real-time polymerase chain reaction, and mean±SEs are shown. **D** and **E**, *Epas1*^*EC-KO*^ mice and littermate controls lacking Cre (*Epas1*^*EC-WT*^) were injected with tamoxifen aged 6 weeks and analyzed 2 weeks later, n=6 each group. En face staining of LOSS and high shear stress (HSS) regions of the aortic arch using anti-CD36 (**D**) or anti-LIPG (**E**) antibodies. Endothelium was costained (endothelial cell; green) and nuclei detected (DAPI [4′,6-diamidino-2-phenylindole]; blue). Scale bar: 20 µm. Each data point represents an animal. Differences between means were analyzed using a 1-way ANOVA (**A**), a Mann-Whitney *U* test (**B** and **C**), or 2-way ANOVA (**D** and **E**).

The potential role of EPAS1 in regulating fatty acid metabolism was investigated by gene silencing in cultured PAEC. A lentiviral-small hairpin RNA effectively reduced EPAS1 at both the mRNA (Figure S12A) and protein (Figure S12B) levels in PAEC cultured under LOSS conditions. Next, the effects of *EPAS1* silencing on oxygen consumption rates were assessed by performing a fuel flux assay using a Seahorse metabolic bioanalyzer. Here, oxygen consumption rate was measured in PAEC exposed to LOSS that were sequentially treated with etomoxir (an inhibitor of long-chain fatty acid oxidation), followed by a combination of UK5099 (an inhibitor of glucose oxidation pathway) and BPTES (an inhibitor of glutamine oxidation; Figure S12C). It was observed that *EPAS1* silencing caused an ≈80% reduction in basal oxygen consumption rate of EC exposed to LOSS (Figure 8A; Figure S12C) and that oxygen consumption rate was strongly dependent on fatty acid β-oxidation (Figure S12D). These data indicate that EPAS1 is a positive regulator of fatty acid β-oxidation–dependent respiration. To further investigate the role of EPAS1 in respiration, we performed a mitostress assay. This assay revealed that EPAS1 small hairpin RNA silencing had a modest but significant impact in reducing maximal respiratory rate (Figure S12E) and ATP production (Figure S12F). Thus, EPAS1 regulates respiration by controlling fatty acid β-oxidation and has relatively modest effects on mitochondrial activity per se.

It is notable that the reduction in oxygen consumption in *EPAS1*-silenced cells could be rescued by supplementing cultures with exogenous OA (Figure 8A). This suggests that EPAS1 may regulate the initiation of the fatty acid β-oxidation pathway by controlling the ability of EC to produce or handle FFAs. This was tested by quantifying levels of 2 molecules that coordinate endothelial handling of FFAs: LIPG (endothelial type lipase G) which hydrolyzes TGs into FFAs at the endothelial surface, and CD36 (cluster of differentiation 36) which transports extracellular FFAs into EC.^36^ Real-time PCR analysis showed that *EPAS1* silencing significantly reduced the expression of *LIPG* and *CD36* at the mRNA level (Figure 8B and 8C). To confirm the influence of *Epas1* on CD36 and LIPG, we performed en face staining of the murine aorta. In WT mice, en face staining of the aortic arch revealed that CD36 and LIPG were significantly enriched at the LOSS region compared with the HSS region (Figure 8D and 8E). The expression of CD36 and LIPG was significantly reduced at LOSS regions in *Epas1*^*EC-KO*^ compared with controls (Figure 8D and 8E), indicating that *Epas1* is required for the enrichment of both these molecules at the LOSS region. To assess whether localized enrichment of CD36 and LIPG at the LOSS region is associated with elevated lipid metabolism, we exposed mice to acute hyperlipidemia using oral gavage of olive oil. Plasma levels of TG were significantly increased at 90-minute postgavage and declined in some animals at 180-minute postgavage (Figure S13A). Notably, en face staining with a lipophilic dye (BODIPY493/503) revealed enhanced accumulation of lipid droplets at the LOSS region of the aortic arch compared with the HSS region at 180 minutes (Figure S13B), suggesting localized enhancement of lipid uptake. Collectively, these data suggest that EPAS1 is required for endothelial fatty acid β-oxidation at atheroprone LOSS regions by positively regulating the expression of CD36 and LIPG, which coordinate FFA handling.

Since HIF1A regulates glycolysis at regions of LOSS,^9^ we assessed the potential contribution of EPAS1 to this pathway using a Glycostress assay. In this assay, PAEC exposed to LOSS were first starved for glucose to establish a basal extracellular acidification rate in a nutrient-deprived setting, followed by the injection of glucose, oligomycin, and 2-DG. Silencing of *EPAS1* reduced both glycolysis (Figure S14A) and glycolytic capacity (Figure S14B; a readout of metabolic plasticity) by ≈15%. We, therefore, investigated the levels of HK2 or PFKFB3, which are key glycolytic modulators that are regulated by HIF1A.^9^ Immunoblotting revealed that silencing of *EPAS1* did not influence the expression of HK2 or PFKFB3 (Figure S14C). Thus, EPAS1 has relatively modest effects on glycolysis through a mechanism that is distinct to HIF1A.

Endothelial fatty acid β-oxidation is primarily used to drive dNTP (deoxyribonucleotide triphosphates) production for DNA repair and proliferation,^37^ and we, therefore, hypothesized that EPAS1 may be required for supporting endothelial proliferation via fatty acid metabolism. This was tested by analysis of the murine aorta by en face staining of Ki67 (a marker of mitotic cells). EC proliferation was significantly reduced in *Epas1*^*EC-KO*^ mice compared with controls (Figure S15A), indicating that endothelial *Epas1* is required for EC proliferation. Consistently, analysis of cultured PAEC exposed to LOSS revealed that proliferation was significantly reduced by silencing of *EPAS1* (Figure S15B). To reconcile the effects of EPAS1 on fatty acid β-oxidation and proliferation, we rescued fatty acid metabolism in EPAS1-silenced PAEC by treating them with exogenous OA. Metabolic rescue significantly enhanced the frequency of EC proliferation in EPAS1-silenced PAEC (Figure S15B), suggesting that EPAS1 drives proliferation by enhancing fatty acid metabolism. Overall, our data reveal that EPAS1 plays an atheroprotective role in regions exposed to disturbed flow by preserving EC metabolic homeostasis and proliferative capacity.

## DISCUSSION

### Metabolic Status Regulates EPAS1 in Obesity

Our observations indicate a preferential expression of EPAS1 at atheroprone sites exposed to disturbed flow, which generates altered mechanical conditions, including LOSS. Here, we demonstrate that LOSS can induce enhanced EPAS1 expression in cultured arterial EC. Under these conditions, oxygen levels are physiological or exceed physiological levels, and therefore we conclude that LOSS can induce EPAS1 independently from hypoxia in arterial EC. Interestingly, empiric measurements of oxygen tensions in instrumented dogs indicate that disturbed flow regions in carotid arteries experience reduced oxygen levels.^38^ Thus, enhanced EPAS1 expression at atheroprone disturbed flow regions may be regulated by LOSS in conjunction with subphysiological oxygen levels.

A pivotal finding from our research highlights the loss of EPAS1-regulated homeostasis during obesity, a global epidemic and major contributor to cardiovascular disease. Our study concludes that obesity leads to the suppression of EPAS1 in arterial EC, unveiling a novel molecular mechanism contributing to obesity-driven atherosclerosis. Our subsequent experiments delineated the mechanism for EPAS1 regulation in obesity, focusing on the potential effects of hyperlipidemic and hyperglycemic metabolic disturbances. Although streptozotocin can cause EC dysfunction,^31^ we did not observe a reduced expression of EPAS1 in streptozocin-treated mice that subsequently developed hyperglycemia. This observation suggested that suppression of EPAS1 in obesity was not due to hyperglycemia. Important insight was gained by treating obese mice with sulforaphane, a natural compound that protects against atherosclerosis via effects on metabolic status and effects of EC physiology.^32,33^ Sulforaphane treatment rescued EPAS1 expression in obese mice. EPAS1 rescue was coupled to lowering of plasma TG levels but was not associated with alteration in plasma cholesterol or glycemia. We, therefore, hypothesized that suppression of EPAS1 in obesity may be related to enhanced levels of circulating TG. Further evidence was obtained via a series of in vitro experiments showing that FFAs can suppress EPAS1 expression in cultured EC, potentially via induction of PHD2. Sulforaphane has direct effects on endothelium by altering oxidant status,^33^ and we observed in cultured cells that sulforaphane partially rescued EPAS1 in FFA-treated PAEC. Thus, sulforaphane rescues EPAS1 by suppressing the TG-FFA pathway and via modulation of cellular redox.

EPAS1 expression was enriched at LOSS regions of the aorta and was also detected in microvascular EC, which experience unidirectional laminar flow. Since microvascular EC experience intermittent blood flow, it is plausible that EPAS1 expression in the microvasculature is regulated by oxygen tension; however, this needs to be tested experimentally. Since microvascular EPAS1 is reduced in obesity, it will be interesting in future studies to discern whether this is linked to increased prevalence of hypertension in obese individuals.

### EPAS1 Protects From Atherosclerosis by Promoting Fatty Acid Metabolism

Arterial regions of uniform flow are characterized by physiological HSS, which activates multiple protective proteins, including endothelial nitric oxide synthase, transcription regulators KLF2, and NRF2 KLK10.^39,40^ Collectively, these molecules coordinate the upregulation of a suite of antiapoptotic and anti-inflammatory molecules to reduce vascular injury and inflammation at HSS sites. In contrast, regions of disturbed flow are characterized by LOSS-dependent activation of multiple proatherogenic pathways,^41^ including BMP4-HOXB9,^42^ GATA4-TWIST1-SNAIL,^43^ and NOTCH4-JAG1,^26^ which collectively increase inflammation and impede vascular repair. Surprisingly, our murine model demonstrates that endothelial EPAS1 exhibits a protective effect against atherosclerosis at an atheroprone region exposed to LOSS. This is analogous to a previous observation that LOSS induces stress response genes, which serve to maintain homeostasis in regions of disturbed flow.^44^ Thus, EPAS1 and the stress response pathway are homeostatic mechanisms that reduce the atherogenic process at predilection sites for atherosclerosis. *Epas1* deletion did not influence plaque composition in the AAV-PCSK9 model. However, it should be noted that this model mimics features of early atherosclerosis and that further research using other models (eg, *ApoE−/−*) is required to fully appreciate the role of *Epas1* in disease progression.

Further investigation into the mechanism of atheroprotection revealed the requirement of EPAS1 for fatty acid β-oxidation in atheroprone endothelium exposed to LOSS. FFAs are generated at the endothelial plasma membrane by hydrolysis of triglycerides via a complex containing LIPG.^36^ They are subsequently transported into the cell via CD36. EPAS1 appears to play a role in fatty acid handling, with genetic deletion of *Epas1* leading to a reduction in LIPG and CD36 protein expression at sites of LOSS. This effect is replicated in PAEC with EPAS1 silencing. Fatty acid β oxidation makes only a minor contribution to ATP production in ECs with glycolysis being the primary driver of ATP production in this context.^45^ Nevertheless, there is growing appreciation of the importance of fatty acid β oxidation as a critical regulator of EC physiology and metabolic homeostasis.^46,47^ Fatty acid β oxidation is required for redox homeostasis via regeneration of NADPH in quiescent endothelium.^48^ It is also needed for dNTP production needed for DNA replication in proliferating endothelium.^37^ Here, we demonstrated using *Epas1*^*EC-KO*^ mice that endothelial expression of *Epas1* positively regulates the expression of CD36 and LIPG, which coordinate FFA entry to the endothelium. Crucially, proliferation was rescued in *EPAS1*-silenced cells by supplementation with exogenous FFAs, suggesting that EPAS1-dependent fatty acid handling is a positive regulator of EC repair under disturbed flow.

Disturbed flow induces multiple injurious stimuli in arterial endothelium, including heightened apoptosis and EndMT,^49^ and therefore we hypothesize that EPAS1-dependent EC proliferation is required for endothelial repair at these regions. This concept is consistent with our observation that endothelial *Epas1* is atheroprotective. However, the relationship between EC turnover and atherosclerosis is intricate, as proliferation is essential for vascular repair, but excessive proliferation may contribute to increased permeability to proatherogenic lipoproteins and endothelial activation. Intriguingly, this balance between the protective and proatherogenic effects of EC proliferation may involve a balance between EPAS1 and HIF1A. Previous research found that endothelial HIF1A promotes atherosclerosis^50^ by driving inflammation and proliferation at atheroprone regions via upregulation of glycolysis.^9–11^ By contrast, we observe that EPAS1-driven proliferation via fatty acid metabolism is coupled to atheroprotection. Given our observation of heterogeneous expression of EPAS1 and HIF1A in atheroprone endothelium, we hypothesize that EPAS1+ HIF1A+ cells are supercharged for excessive proliferation because they are primed for HIF1A-glycolytic energy production and EPAS1-fatty acid β oxidation-driven dNTP production, leading to a proatherogenic phenotype. By contrast, we hypothesize that EPAS1+ HIF1A− cells are programmed for homeostatic proliferation that slows the initiation of atherosclerosis. However, further experimentation is needed to explore these concepts. Identifying mechanisms that shift the balance of HIF transcription factors to suppress HIF1 and enhance EPAS1 may offer therapeutic opportunities to promote vascular repair and reduce atherosclerosis.

In summary, our findings underscore the preferential expression of EPAS1 at atheroprone sites, where it reduces atherosclerosis by promoting fatty acid metabolism to support endothelial repair. This protective pathway is compromised by obesity, revealing a novel molecular link connecting systemic cardiovascular risk to EC dysfunction. These findings hold potential implications for the clinical management of obese individuals with cardiovascular risk.

## ARTICLE INFORMATION

### Acknowledgments

The authors thank the Adipositazentrum at Limmattal-Hospital, Schlieren, for their support in collecting blood samples in patients with obesity.

### Sources of Funding

This study is supported by the British Heart Foundation RG/19/10/34506 (P.C. Evans), British Heart Foundation Intermediate Fellowship (FS/18/2/33221; J. Serbanovic-Canic), United Kingdom Research and Innovation (UKRI) Future Leaders Fellowship MR/T04201X/2 (M. Fragiadaki), A*STAR Intramural Funding (W. Han), Strategic Program Fund (BBI; W. Han), Central Research Fund (W. Han), Swiss National Science Foundation (PRIMA: PR00P3_179861/1; E. Osto), Swiss Life Foundation, Switzerland (E. Osto), Alfred and Annemarie von Sick Grants for Translational and Clinical Research Cardiology and Oncology (E. Osto), Heubergstiftung and the Swiss Heart Foundation, Switzerland (E. Osto), National Institutes of Health grants (R01 HL148239 and R01 HL164577; C. Miller), Leducq Foundation Network of Excellence grant “PlaqOmics” (18CVD02; C. Miller), A*STAR Research Attachment Program (D. Pirri), Academy of Medical Sciences Springboard Award (SBF005/1064; S.P. Allen), PGC 2018-094025-B-I00 funded by MCIN (Ministerio de Ciencia e Innovación)/AEI (Agencia Estatal de Investigación)/10.13039/501100011033 (G. Vilahur), and Fondo Europeo de Desarrollo Regional A way of making Europe (G. Vilahur).

### Disclosures

None.

### Supplemental Material

Tables S1–S3

Figures S1–S15

## Supplementary Material
