# Supplementary material for: EPAS1 Attenuates Atherosclerosis Initiation at Disturbed Flow Sites Through Endothelial Fatty Acid Uptake
Source: Circ Res. 2024 Sep 5;135(8):822–37. doi: 10.1161/CIRCRESAHA.123.324054 (PMC11424061; doi:10.1161/CIRCRESAHA.123.324054)
Supplement: Supplementary file 3 [file res-135-822-s003.pdf]

SUPPLEMENTAL MATERIAL

Expanded Materials and Methods

Online Figures S1-S15

Supplemental Tables

Expanded Materials and Methods

Mice

Mice with inducible deletion of *Epas1* in EC were generated by crossing *Epas1*$^{fl/fl}$ [20] mice with *CDH5*$^{Cre\text{-}ERT2}$ mice [21]. PCR primers used for genotyping are shown in Table S1. To activate Cre, tamoxifen (Sigma) in corn oil was administered intraperitoneally (IP) for 5 consecutive days (2 mg/mouse/d) and mice were used for experimentation two weeks later. Hypercholesterolemia was induced by IP injection of adeno-associated virus (AAV) containing a gain-of-function mutated version of proprotein convertase subtilisin/kexin type 9 (rAAV8-D377Y-mPCSK9) gene (Vector Core, North Carolina). Virus particles were injected at a titre of $6*10^{11}$ in single dose to each mouse followed by a high fat diet (HFD; SDS UK, 829100) for 8 weeks as previously described[22].

Some C57BL/6J mice aged 9 weeks were fasted for 8 hours before exposure to 10 ml/kg body weight olive oil by oral gavage. Blood plasma samples for measurement of plasma triglycerides were collected by tail vein bleeding. Thoracic aortae and blood plasma samples were collected 90 and 180 minutes after gavage to study lipid droplets in vessels and triglycerides levels in plasma samples.

Lep$^{ob/ob}$ mice (C57BL/6J) were obtained from The Jackson laboratory (strain: #00632) and were bred as heterozygotes. Wild-type littermates *(Lep*^{WT}) were used as controls. *Lep*^{WT} and *Lep*$^{ob/ob}$ mice were maintained using a standard rodent chow diet. To confirm the presence of obesity, Lep *Lep*$^{ob/ob}$ and *Lep*^{WT} mice were monitored for body weight every week. In addition, obesity was induced in C57BL/6N wild type mice by

exposing them for 25 weeks *ad libitum* to a western diet containing 60% of fat (D12492i, Research Diets) as described previously[23], in parallel control groups were kept on a chow diet for the duration of the experiment. Alternatively wild-type C57BL/6J mice were fed a chow diet for 10 weeks and then exposed to 10 weeks of HFD containing 45% fat (D12451) with matching sucrose contents in control chow diet (D12450H). Control mice were maintained on a sucrose matched control chow diet for 20 weeks. Blood pressure measurements were made using plethismographer CODA (Kent Scientific). Mice were placed into a plastic restrainer-tube covered by a warm pad (37ºC) and left undisturbed for five minutes. The plethismographer was then placed on the mouse tail and secured with a plastic support. Mice were trained for 5 to 7 consecutive days before performing final measurements. For each mouse, a minimum of 5 readings per session were made. Hyperglycaemia was induced in C57BL/6J mice using Streptozotocin, hereafter referred to as STZ (S1030, Sigma). Mice aged 20 weeks were injected with STZ (150 mg/kg) intraperitoneally and then analysed at 22 weeks. Some C57BL/6N mice exposed to a HFD containing 60% of fat were treated with sulforaphane (SFN; Cayman Chemicals) by I.P injection 5mg/kg for 3 days.

Wild-type C57BL/6N, C57BL/6J and transgenic mice were housed under specific-pathogen free conditions. Both male and female mice were used in this study. Control groups are derived from littermate mice ($Epas1^{EC\text{-}KO}$ and $Lep^{ob/ob}$) or by using age and gender matched mice for HFD diet and injectable treatment controls (SFN and STZ). Mice sources are further listed in the supplementary Major Resource Table. Animal care and experimental procedures were carried out under licenses issued by the UK Home Office and local ethical committee approval was obtained. All animal

procedures conformed to the guidelines from Directive 2010/63/EU of the European Parliament on the protection of animals used for scientific purposes and to IACUC guidelines. All experiments involving animals in Singapore were reviewed and approved by the IACUC of A*STAR Biomedical Sciences Institutes. All mice were on a C57BL/6 background.

Shear stress maps

The lumen geometry of the mouse aorta was derived previously[24]. The unsteady Navier-Stokes equations were solved numerically using the Finite Volume Method (FVM) using the specialized in-house haemodynamics solver haemoFOAM (https://github.com/TS-CUBED/haemoFoam). The vessel geometry was modelled as rigid. Blood was modelled as a non-Newtonian Quemada fluid with an average haematocrit of 45 percent. The maximum Reynolds number based on the inlet diameter is around 500, therefore, the flow was modelled as laminar. The time dependent mean velocity was digitized from US Doppler images. The aortic root cross section was extruded by 10 diameters to allow for development of the flow and prescription of a Dirichlet inlet boundary condition for the normal velocity as a plug profile at the extruded section. At the exit of the aorta descendent and the branches, a Neumann outlet boundary condition was used with a flow split of 70, 16, 8 and 6 percent for descendent aorta, innominate, common carotid and subclavian, respectively. Numerical solutions were obtained using second order spatial and second order implicit temporal discretization schemes. The mesh size was 0.5 million cells and the time step size was chosen as 1/800th of the cycle time. From the time-dependent WSS, the time-averaged WSS and the oscillatory shear stress index (OSI) at the wall were calculated:

$$\overline{\tau} = \frac{1}{N}\sum_{N}\tau_n$$

$$OSI = 0.5\left(1.0 - \frac{1}{N}\frac{|\sum_N \tau_n|}{\sum_N |\tau|}\right)$$

Antibodies

Primary and secondary antibodies and species-specific control antibodies and the concentrations used are documented in Table S2 and in the Major Resources Table.

Endothelial RNA extraction

RNA was extracted from aortic EC using the Qiazol flushing method as described [25].

Atherosclerosis plaque analysis

Mice were killed using pentobarbital and perfusion-fixed with PBS followed by 4% paraformaldehyde. The aorta was dissected, gently cleaned of adventitial tissue, and stained *en face* with Oil Red O (Sigma). The lesion surface area was analysed using NIS elements analysis software (Nikon, NY). For aortic root sections, the upper portion of the hearts were dissected horizontally at the level of the atria and placed in 30% sucrose for 24 h before embedding in paraffin. Serial 7 μm sections were processed for immunohistochemistry and for staining with Mayer's haematoxylin and eosin. ImageJ software was used to calculate the lesion area as a percentage of the total aortic root area, and the area occupied by macrophages and collagen. Quantification of elastin breaks was performed using elastin van Gieson stained sections (3 sections per animal), whilst the number of elastin breaks per section were counted and expressed as average number of elastin breaks per animal. Quantification was performed for three sections per animal.

Murine peripheral blood measurements

Blood samples were collected by terminal cardiac puncture and serum was separated by centrifugation and analysed using a colorimetric assay (ab65390, Abcam) for detection of total plasma cholesterol and non-HDL cholesterol. Serum and plasma levels of EPAS1 were quantified by ELISA using a commercial kit (LSBio, LS-F19020). Blood glucose and triglycerides levels in venous blood were measured using Accucheck glucometer (Roche Diagnostic) and Accutrend (COBAS, Roche diagnostic), Lipid Panel Test Strips (BHR Biosynex) and the CardioChek Professional Analyser test system.

Immunofluorescent staining of murine endothelium

Animals were killed by I.P. injection of pentobarbital and vasculature was perfused *in situ* with PBS and then perfusion-fixed with 4% Paraformaldehyde prior to harvesting. The expression levels of specific proteins were assessed in EC at regions of the inner curvature exposed to low oscillatory shear stress (LOSS) and outer curvature exposed to physiological high shear stress (HSS) of murine aortae by *en face* staining using specific primary antibodies. BODIPY 493/503 diluted in PBS at the final concentration of 0.01mg/ml was applied for 30mins to mouse vessels to delineate lipid droplets. EC were identified by co-staining using anti-CD31 or VE-cadherin (CDH5) antibodies. Nuclei were identified using TO-PRO-3. Stained vessels were mounted prior to visualization of endothelial surfaces *en face* using confocal microscopy (Olympus SZ1000 confocal inverted microscope). Antibody specificity was confirmed by staining using species-specific IgG controls and subtracting background fluorescence signals. The expression of specific proteins at each site was assessed by quantification of mean fluorescence intensities with standard error of the

mean or analysis using ImageJ software (1.49p) to calculate the frequency of positive cells. Microvasculature was analysed by immunofluorescent staining as described[26]. Details of all primary and secondary antibodies used in this manuscript are provided in Table S2 and in the supplementary Major Resource Table.

scRNAseq

scRNAseq libraries were generated from age and sex-matched *Epas1*$^{EC\text{-}KO}$ and *Epas1*$^{EC\text{-}WT}$ mice using the SORT-seq protocol as described previously[27]. 454 cells were analysed after quality control; 262 cells (58%) from *Epas1*$^{EC\text{-}WT}$ mice and 192 cells (42%) from *Epas1*$^{EC\text{-}KO}$ mice. Dimensional reduction and clustering of our scRNA-seq dataset were performed using the R package Seurat (v3.1). Differential expression analysis was performed between clusters. Genes were defined as enriched when their expression in a particular cluster was significantly different from the rest of the clusters using the Wilcoxon rank-sum test with p adjusted value < 0.001. These genes were selected for subsequent gene ontology (GO) analysis using the DAVID Functional Annotation Bioinformatics Analysis software.

Culture of EC and exposure to WSS

Pig aortae from 4-6 months old animals were obtained immediately after slaughter from a local abattoir. Porcine aortic EC (PAEC) were harvested using collagenase (1 mg/ml for 10 minutes at room temperature) and were cultured using M199 (Gibco) media. Experiments were performed using cells from multiple donors that were not pooled. A total of 30000 PAEC at passage 3-5 were seeded onto gelatin-coated Ibidi μ-Slides I$^{0.4}$ (Luer ibiTreat, ibidi™) and used when fully confluent. Flowing medium was then applied using the Ibidi pump system to generate HSS (13 dyn/cm^2), LSS (4

dyn/cm^2) or low oscillatory WSS. For low oscillatory WSS, PAEC were exposed to a repeated cycle of 2 h of oscillatory flow (+/- 4 dyn/cm^2, 0.5 Hz), followed by 10 min of unidirectional flow (+4 dyn/cm^2), to ensure redistribution of nutrients[28]. The slides and pump apparatus were placed in a cell culture incubator at 37°C. Alternatively, PAECs were cultured in a 6 well plate and exposed to shear stress using the orbital system as described[9]. Some sheared cultures were also exposed to 0.25 mM free fatty acids (FFAs) i.e. oleic acid (OA) or palmitic acid (PA).

Gene silencing

PAEC cultures were transfected with a pool of shRNA sequences targeting porcine *EPAS1* (V3SVHSHC, Dharmacon) or using the Dharmacon™SMARTvector Lentiviral system following the manufacturer's instructions. Non-targeting lentiviral shRNA sequence was used as a control (V16060303, Dharmacon). All lentiviral transductions were performed at 30 multiplicity of infection in the presence of 6μg/ml of DEAE-dextran hydrochloride (Sigma).

Real time PCR

RNA was extracted using the RNeasy Mini Kit (74104, Qiagen) and reverse transcribed into cDNA using the iScript cDNA synthesis kit (1708891, Bio-Rad). QRT-PCR was used to assess the levels of transcripts with gene-specific primers (Table S1). Reactions were prepared using SsoAdvanced universal SYBR®Green supermix (172-5271, Bio-rad) and following the manufacturer's instructions, and were performed in triplicate. Expression values were normalized against the house-keeping genes *B2M* (pig), Hprt (human, mouse). Data were pooled from at least three independent donors and mean values were calculated with SEM.

XF24 metabolic fuel flex assay.

PAEC at passage 5 were plated in a Cell Tak-coated XF24 cell culture plate (Agilent) at the density of 45000 cells per well in XF assay media pH 7.4 (Agilent) supplemented with 2 mM glutamine and 25 mM glucose. In the mitofuel assay, OCR was measured for 32 min prior to the addition of Etomoxir (6 μM) to analyse the contribution of fatty acid β -oxidation to OCR, then the addition of BPTES (1μM) and UK5099 (6 μM) to analyse the contribution of glycolysis and glutamine to OCR. The contribution of fatty acid β-oxidation to OCR was expressed as fatty acid dependency and calculated as per manufacturer's instructions. The mitostress assay involved OCR measurements during sequential inhibition of the complex V using oligomycin (25 μM), followed by the injection of FCCP (40 μM) to maximise the respiratory capacity of the electron chain, and Rotenone/Antimycin A (20 μM/2 0μM) mix to inhibit complex I and III. Maximal respiration and ATP production were calculated as per manufacturer's instruction.

Extracellular acidification rate (ECAR) was measured basally after glucose starvation and after addition of oligomycin to assess glycolytic capacity. Glycolysis was suppressed via addition of 2 deoxyglucose (2DG) to allow measurement of non-glycolytic ECAR. Glycolytic ECAR was calculated by subtracting the ECAR value in the presence of 2DG from the ECAR value prior to oligomycin injection. Glycolytic capacity was calculated by subtracting ECAR in the presence of oligomycin from ECAR in the presence of 2DG.

Immunofluorescent staining of cultured EC

PAEC were fixed with paraformaldehyde (4%) and permeabilised with Triton X-100 (0.1%). Following blocking with goat serum for 30 min, monolayers were incubated for 16 h with primary antibody against Ki67 and AlexaFluor488. Nuclei were identified using DAPI (Sigma). Images were taken with a widefield fluorescence microscope (LeicaDMI4000B) and analysed using ImageJ software (1.49p) to calculate the frequency of positive cells. Antibody specificity was confirmed by staining using species-specific IgG controls and subtracting background fluorescence signals.

Immunoblotting

Total cell lysates were isolated using lysis buffer (containing 2% SDS, 10% Glycerol and 5% β-mercaptoethanol) and immunoblotted using primary antibodies. HRP-conjugated secondary antibodies (Dako) and chemiluminescent detection was carried out using ECL Prime® (GE Healthcare). Membranes were imaged using the Gel Doc XR+ system (Biorad). Alternatively immunoblots were incubated with Dylight 680 or 800 secondary antibodies (ThermoFisher Scientific) and imaged using a Odyssey CLx imaging system (LI-COR Biosciences).

Clinical adipose and serum samples.

Human subcutaneous adipose tissues (n = 29) were collected from obese (BMI>30) and non-obese (BMI<30) individuals. These tissues were obtained as surgical discards from patients undergoing abdominal surgeries. Following surgical removal, the samples were promptly placed in ice-cold HEPES buffer prior to EC isolation as described[31]. De-identified demographic data of the patients were retrieved using the Generic Clinical Research Database at the Medical College of Wisconsin. All

procedures were approved by the Institutional Review Board of MCW and Froedtert Hospital. Peripheral blood samples were taken from healthy non-obese subjects (n=14) and from severe obese subjects with BMI>38 (n=15) with approval from the local Research and Ethic Committees in Zurich, Switzerland approved (Ethic Nr.KEK-ZH_Nr.2013-0389). Serum samples were generated immediately and stored at -80°C until analysis. EPAS1 levels in serum were quantified by ELISA using a commercial kit (CUSABIO, CSB-E12113h). ELISA was used to quantify levels of FFAs (Abcam, AB65341) and total antioxidant capacity (Abcam, AB65329). All patients gave written informed consent. Studies were performed according to the principles of the Declaration of Helsinki.

Statistical analysis

Statistical analysis was performed using GraphPad Prism software. Data are presented as mean values ± SEM. Representative images were chosen to represent the average results achieved within the experiment. Data were tested for normality of distribution using the Shapiro-Wilk normality test. Data that followed a parametric distribution were analysed using ANOVA or t-tests. In case of deviation from normality or in presence of a small sample sizes (N<6) a non-parametric Mann-Whitney U test, Kruskal-Wallis or aligned ranked transformed ANOVA was used. Multiple comparisons in ANOVA were corrected using the Sìdak multiple comparison test. p-values smaller than 0.05 were considered significant. The p-value and the test performed are indicated in the figure and figure legend, respectively.

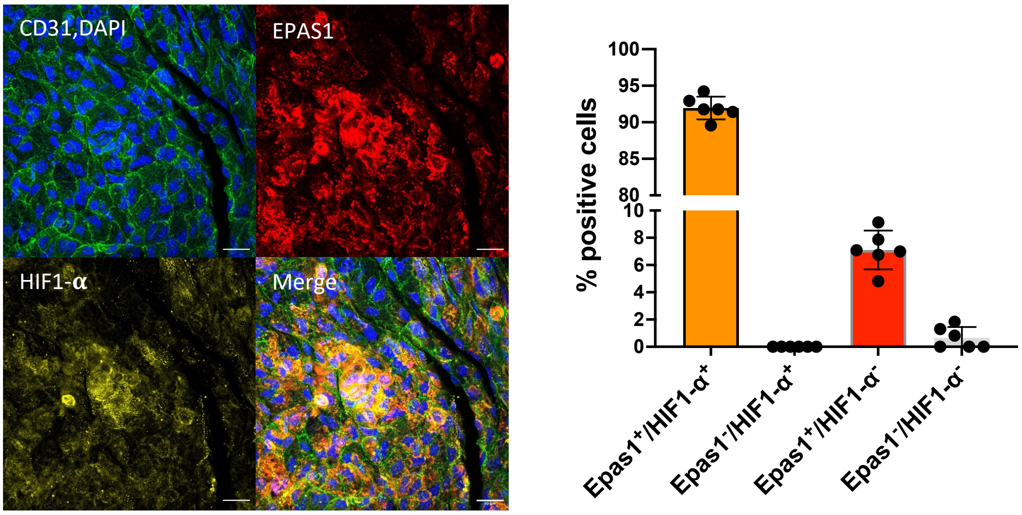

Figure S1. Co-expression of EPAS1 and HIF1A at an atheroprone region.
Aortic arches were isolated from C57BL/6J mice (N=6) aged 6-8 weeks and *en face* immunostaining was performed using anti-EPAS1 (red) and anti-HIF1A (yellow) antibodies. Endothelium was co-stained (CD31; green) and nuclei detected using DAPI (blue). The proportions of EPAS1+/HIF1A+, EPAS1+/HIF1A-, EPAS1-/HIF1A+ and EPAS1-/HIF1A- cells at the LOSS region were calculated. Mean levels +/- standard deviations are shown. Each data point represents an animal.

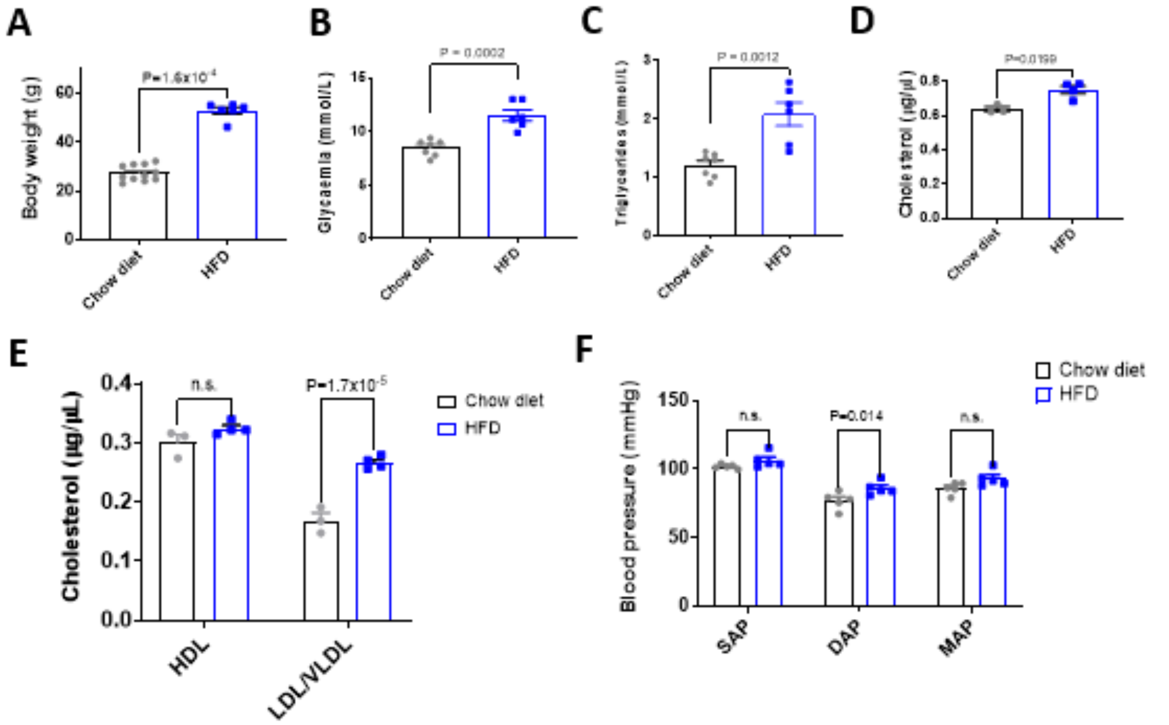

Figure S2. Metabolic changes in obese mice fed a high fat diet.
C57BL/6N mice aged 5 weeks were exposed to HFD (N=6) or to standard chow (N=11) for 25 weeks. Body weight (A), glycaemia (B), plasma triglycerides (C) were measured. Total plasma cholesterol (D) and HDL cholesterol and LDL/VLDL cholesterol (E) were quantified in chow fed mice (N=3) and HFD mice (N=4). (F) Systolic (SAP), diastolic (DAP) and mean (MAP) arterial blood pressures were measured in 5 mice per group. Each data point represents an animal. Differences between means were analysed using a Mann-Whitney U test in A,D, t-test in B,C or using an aligned ranked transform ANOVA in E and F.

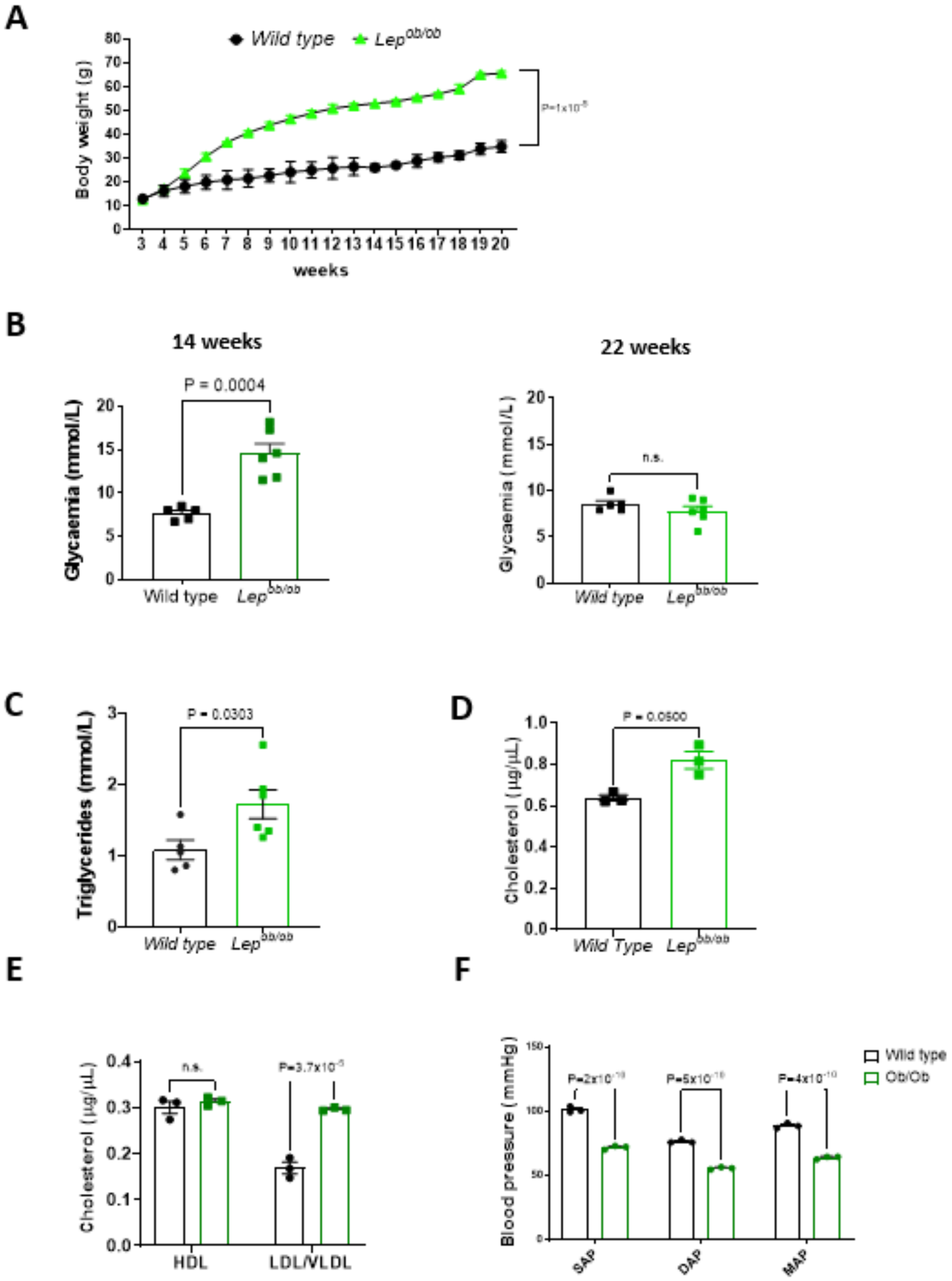

Figure S3. Metabolic changes in *Lep$^{ob/ob}$* mice.
(A) Body weight was measured weekly in *Lep$^{ob/ob}$* mice and littermate controls (wild type) from 3-20 weeks. (B) *Lep$^{ob/ob}$* mice (N=6) and littermate controls (wild type, N=5) aged 14 and 22 weeks were analysed for glycaemia. (C-F) *Lep$^{ob/ob}$* mice and littermate controls (wild type) aged 22 weeks were analysed for plasma triglycerides (C), total plasma cholesterol (D) and HDL cholesterol and LDL/VLDL cholesterol (E). (F) Systolic (SAP), diastolic (DAP) and mean (MAP) arterial blood pressures were measured in *Lep$^{ob/ob}$* mice and littermate controls (wild type) aged 22 weeks, N=3 per group. Each data point represents an animal. Differences between means were analysed using a t-test in A, a Mann-Whitney U test in B, C, D or an aligned ranked transform ANOVA in E and F.

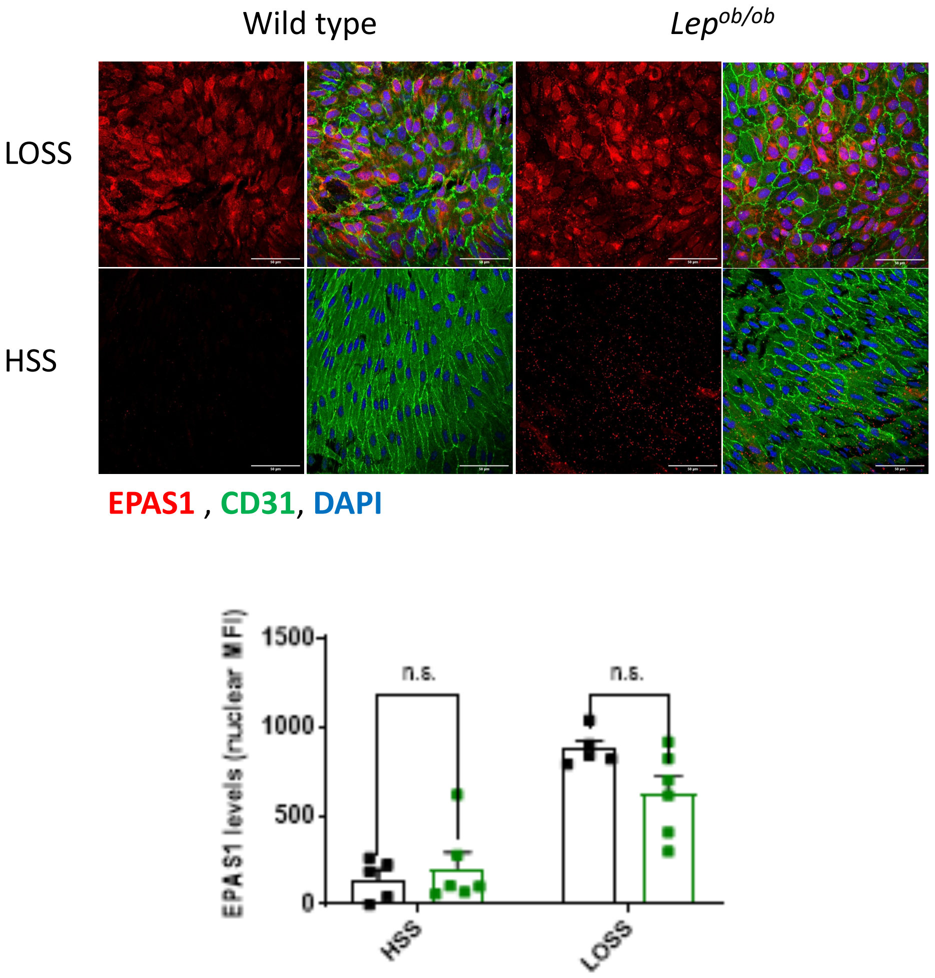

Figure S4. EPAS1 levels are not reduced in presence of hyperglycaemia.
Lep$^{ob/ob}$ mice (N=6) and littermate controls (wild type, N=5) aged 14 weeks were analysed. Aortic EC were stained *en face* using anti-EPAS1 antibodies (red) and fluorescence was quantified at LOSS and HSS regions. Endothelium was co-stained (CD31; green) and nuclei detected using DAPI (blue). Scale bar 50μm. Each data point represents an animal. Differences between means were analysed using an aligned ranked transform ANOVA.

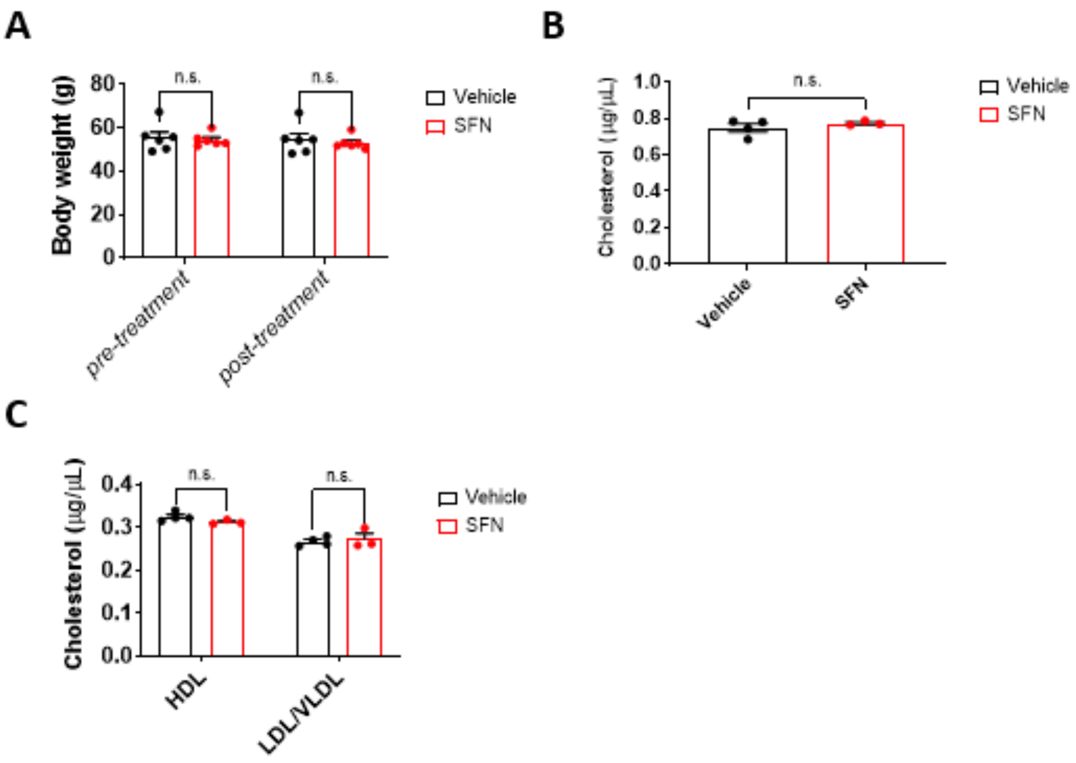

Figure S5. SFN does not regulate cholesterol levels or body weight.
C57BL/6N mice aged 5 weeks were exposed to HFD for 25 weeks (pretreatment group). They were then treated with sulforaphane (SFN; daily I.P. injections 5 mg/kg for 3 days), or vehicle treated for 3 days. N=6 each group with both groups receiving a HFD for that period. Body weight (A) was measured before and after the treatment with SFN and total plasma cholesterol (B), and plasma HDL and LDL/VLDL cholesterol levels (C) were measured in N=3 mice each group. Each data point represents an animal. Differences between means were analysed using two-way ANOVA (A), a Mann-Whitney U test (B) or an aligned ranked transform ANOVA (C).

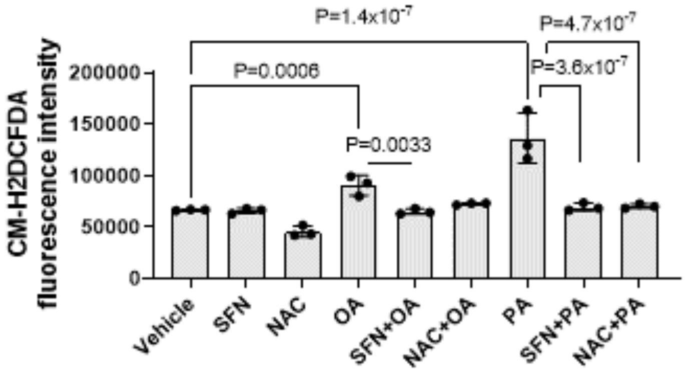

Figure S6. SFN reduces reactive oxygen species in EC exposed to LOSS.
PAEC (N=3) were exposed to LOSS for 72 h using the orbital system in the presence or absence of OA (0.25 mM) or PA (0.25 mM). Some cultures were treated with SFN (10μM) or NAC (1mM) either alone or together with OA or PA. Levels of reactive oxygen species were quantified in live cells using CM-H2DCFDA. Mean fluorescence +/- standard deviations are shown. Each data point represents a biological replicate. Differences between means were analysed using an aligned ranked transform ANOVA.

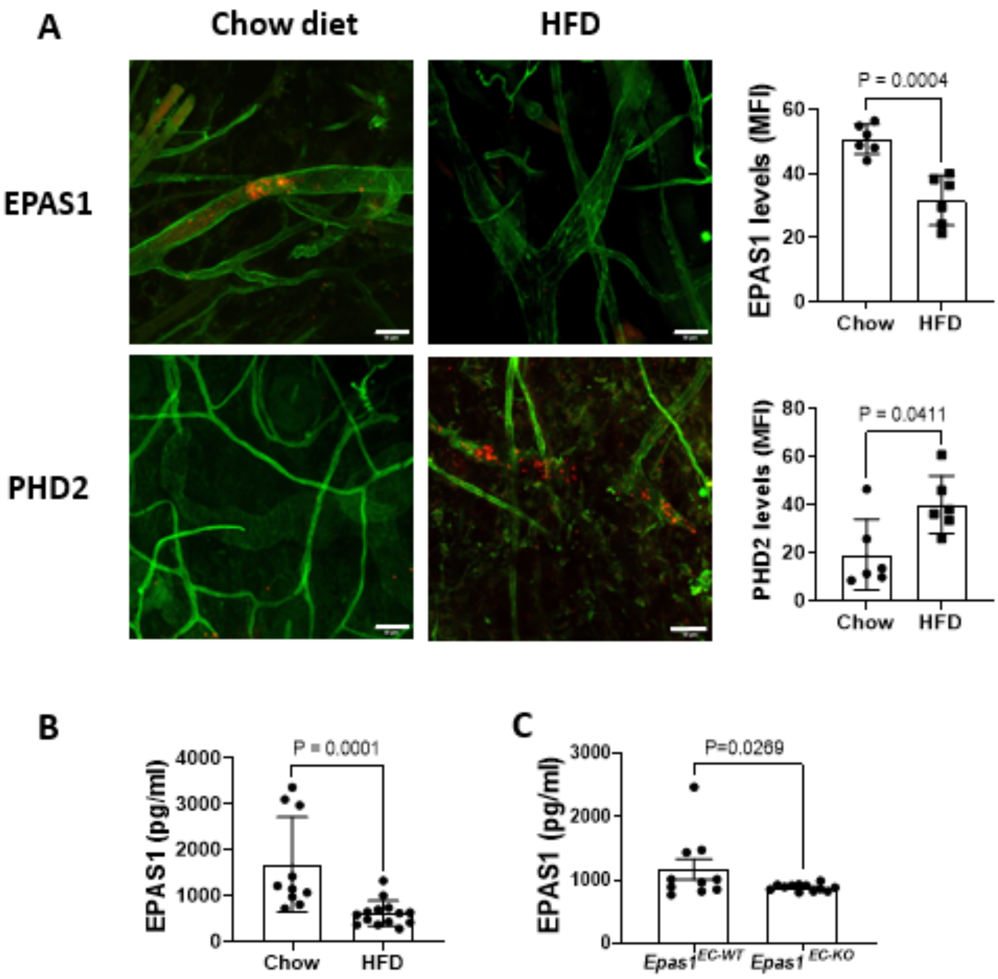

Figure S7. Microvascular EPAS1 is reduced in murine obesity.
C57BL/6N mice aged 10 weeks were exposed to HFD (N=6) or to standard chow (N=6) for 10 weeks. (A) Immunofluorescent staining was used to quantify levels of EPAS1 (red) or PHD2 (red) in dermal microvasculature. EC were identified by co-staining using anti-CD31 antibodies (green). Scale bar 50µm. Mean fluorescent levels +/- standard deviations are shownDifferences between means were analysed using a t-test. (B) Serum levels of EPAS1 were measured and mean values are shown with standard deviations. (C) Plasma levels of *Epas1*$^{EC\text{-}KO}$ mice (N=10) and littermate controls (*Epas1*$^{EC\text{-}WT}$, N=12) exposed to HFD for 8 weeks were measured and mean values are shown with standard deviations. Each data point represents a biological replicate. Differences between means were analysed using a t-test (A) or a Mann-Whitney U test (B, C).

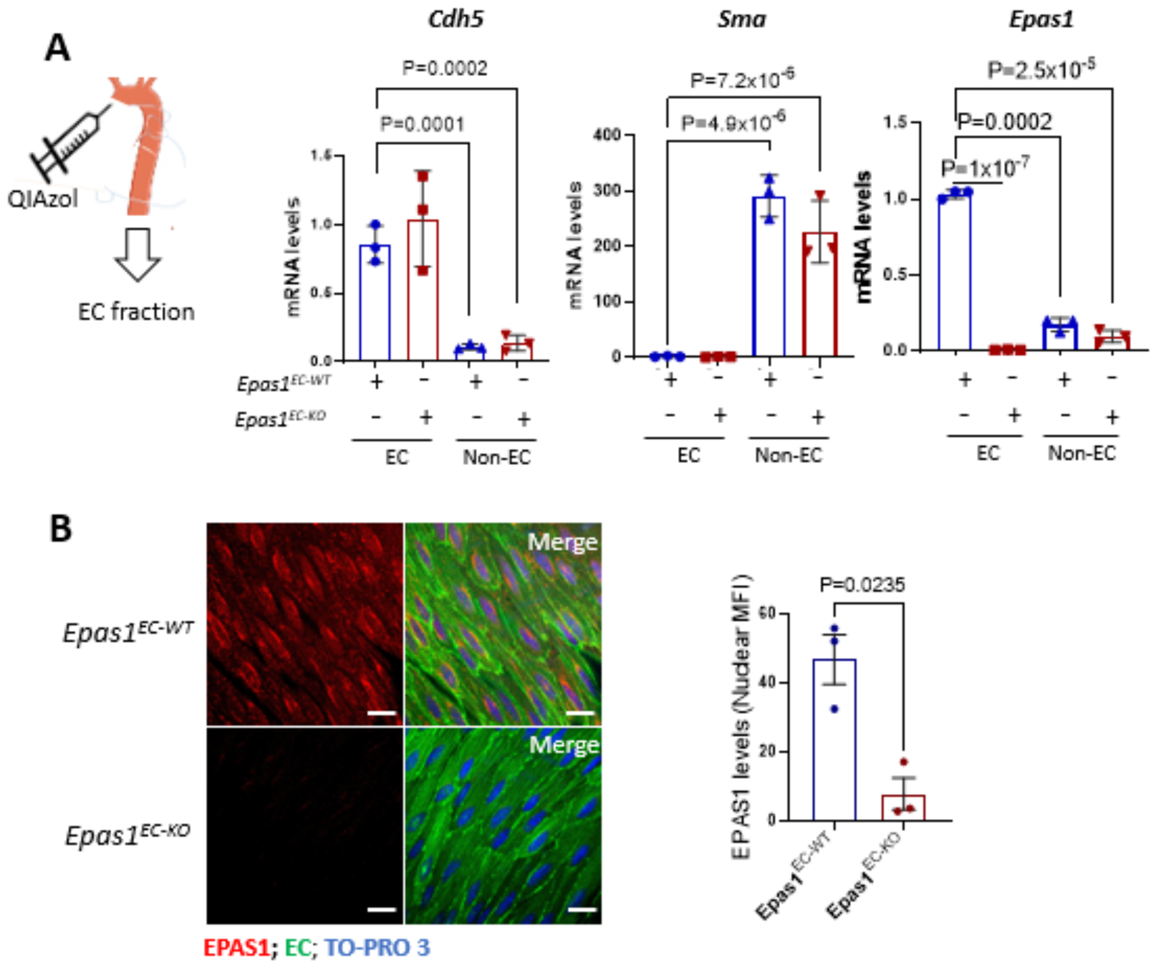

Figure S8. Validation of EPAS1 knock-down in mouse aorta.
Epas1$^{EC\text{-}KO}$ mice (N=3) and littermate controls lacking Cre (*Epas1*$^{EC\text{-}WT}$, N=3) were injected with tamoxifen aged 6 weeks and analysed 2 weeks later. (A) RNA was extracted from EC fractions (by Qiazol flushing) and residual tissue (Non-EC). Levels of *Cdh5* (EC marker), *Sma* (smooth muscle cell marker) and *Epas1* mRNA were quantified by qRT-PCR. *Cdh5* was enriched in EC fractions, whereas *SMA* was enriched in Non-EC fractions. *Epas1* was reduced in EC fractions from *Epas1*$^{EC\text{-}KO}$ mice. (B) The expression of EPAS1 protein (red) was visualized in the murine aorta by *en face* staining. Endothelium was co-stained (EC; green) and nuclei detected using TO-PRO-3 (DNA; blue), N=3. Scale bar 20µm. Each data point represents an animal. Differences between means were analysed using using an aligned ranked transform ANOVA test (A) or a Mann-Whitney U test (B).

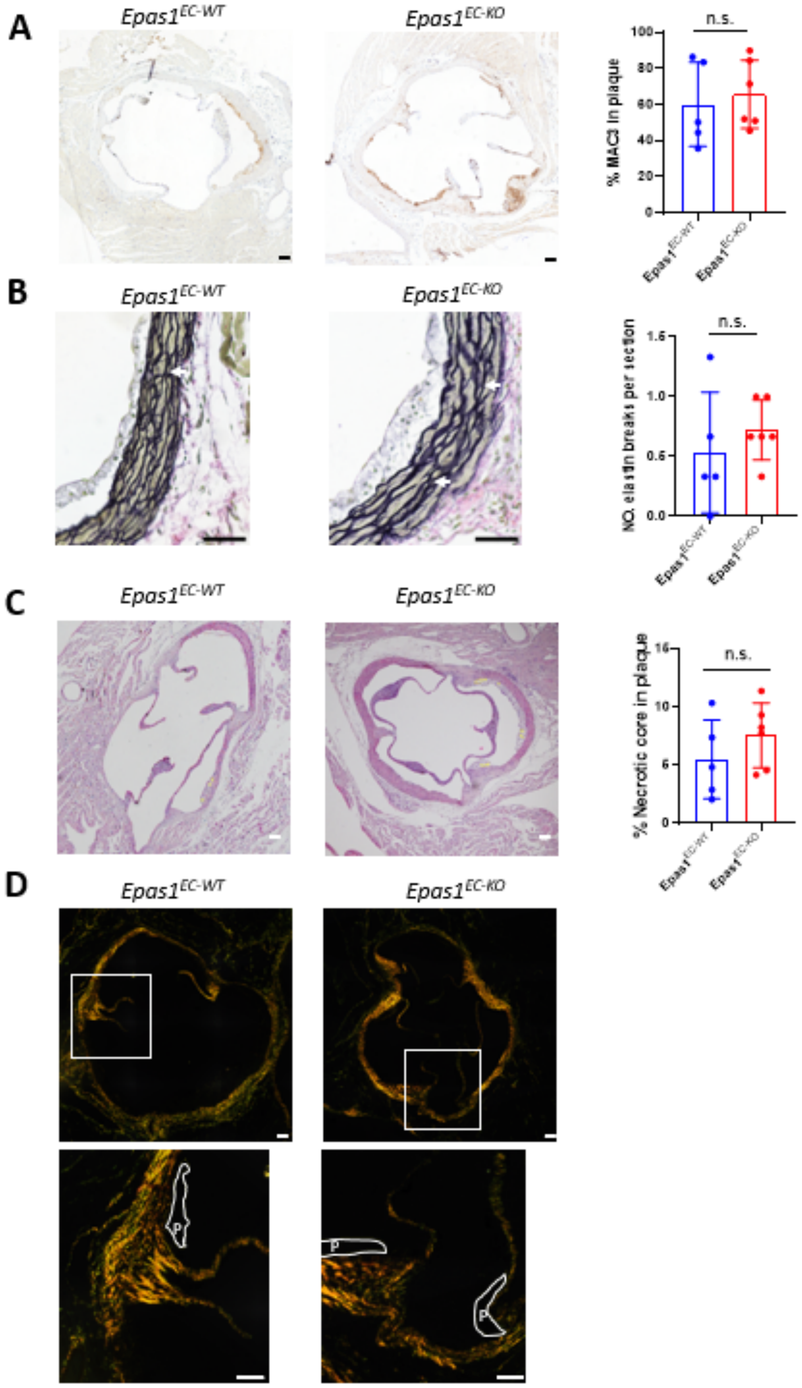

Figure S9. Endothelial Epas1 effects on plaque composition.
Epas1^{EC-KO} mice (N=6) aged 6 weeks and *Epas1^{EC-WT}* mice (N=5) received five intraperitoneal injections of tamoxifen and one injection of PCSK9-AAV virus at specified time points. After 8 weeks fed with high fat diet, the mice were culled and aortic roots were analysed. Representative images are shown, scale bar 200µm. (A) Plaques were stained using anti-MAC3 antibodies to detect macrophages and the proportion of plaque area occupied by macrophages was quantified. The average number of elastin breaks (B; arrows) and the proportion of plaque occupied by necrotic material (C; indicated by yellow line) was quantified. Each data points represents one mouse and mean ± SEM are shown. Differences between means were analysed using a Mann-Whitney U test. (D) Plaques were stained using Picrosirius Red to detect collagen and analysed using polarized light microscopy. Collagen was not detected within any of the plaques (identified using white line; P).

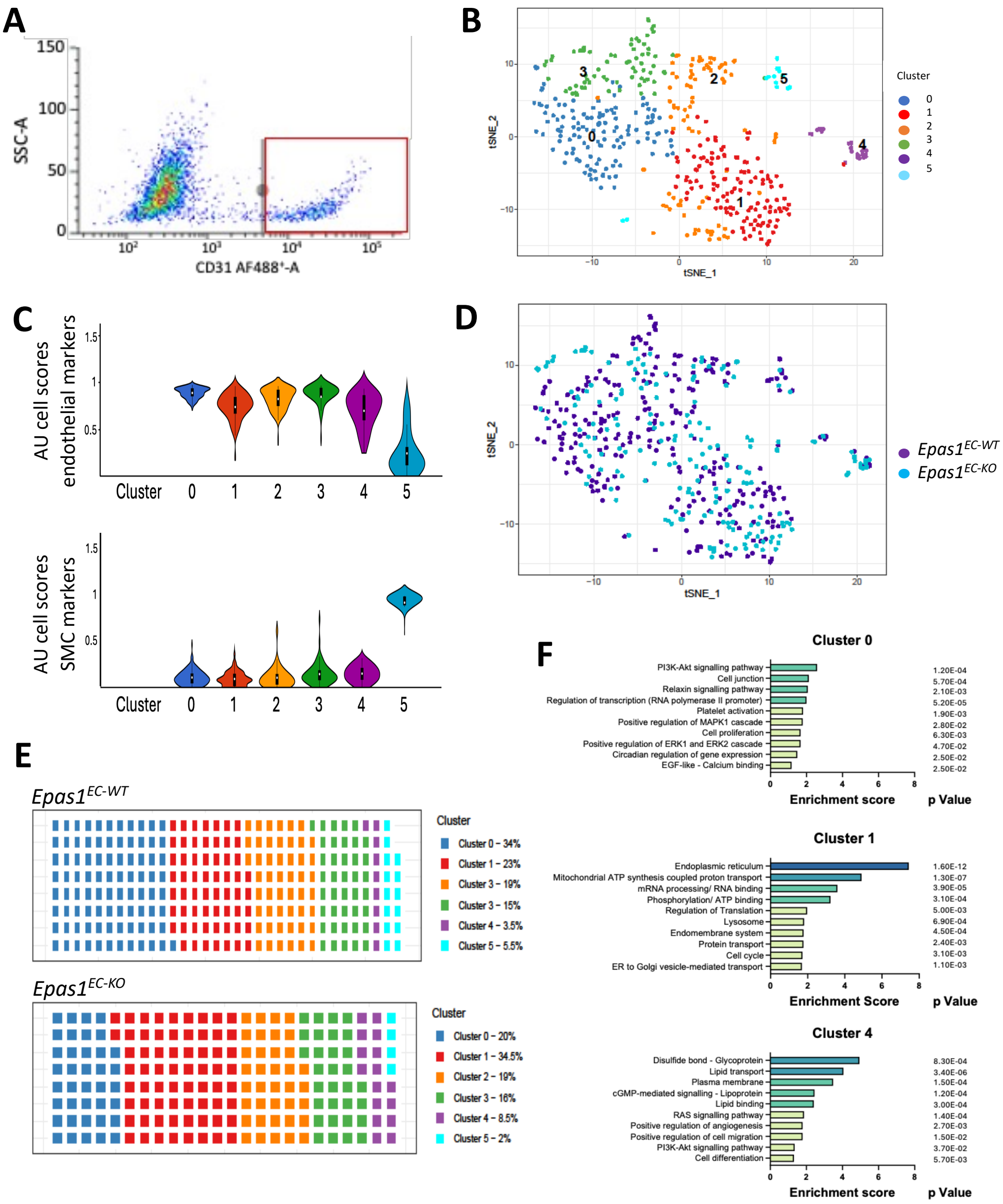

Figure S10. scRNA-seq analysis of murine aortic endothelial cells. *Epas1*$^{EC\text{-}KO}$ mice and littermate controls lacking Cre (*Epas1*$^{EC\text{-}WT}$) were injected with tamoxifen aged 6 weeks and analysed 2 weeks later. Aortas from *Epas1*$^{EC\text{-}KO}$ and *Epas1*$^{EC\text{-}WT}$ littermate control mice were processed to generate single cells and then analysed by FACS of CD31$^+$ CD45$^-$ cells coupled to scRNAseq. (A) Representative flow cytometry profile of CD31 staining after gating out CD45-positive cells. CD31-positive cells (red box) were sorted into a 384-well plate for scRNAseq. (B) t-SNE representation of single-cell transcriptomes from *Epas1*$^{EC\text{-}KO}$ and control mice coloured by cluster assignment. Clusters were identified using unbiased hierarchical clustering. (C) Five distinct clusters (0-4) exhibited enrichment of multiple endothelial markers and were therefore defined as endothelial cells. Cluster 5 was enriched for multiple VSMC markers including and was therefore defined as VSMCs. VSMC: vascular smooth muscle cells. (D) t-SNE showing the cell contribution of *Epas1*$^{EC\text{-}KO}$ and *Epas1*$^{EC\text{-}WT}$ mice to each subpopulation. (E) Cell distribution across cell clusters in *Epas1*$^{EC\text{-}KO}$ and *Epas1*$^{EC\text{-}WT}$ mice. Clusters 1 and 4 are largely composed of ECs derived from *Epas1*$^{EC\text{-}KO}$ mice whereas cluster 0 is mainly composed of ECs derived from control mice. (F) Enrichment scores of enriched GO pathways for clusters 0, 1 and 4. Differences in means were analysed using the Wilcoxon Rank Sum Test and p-values are annotated.

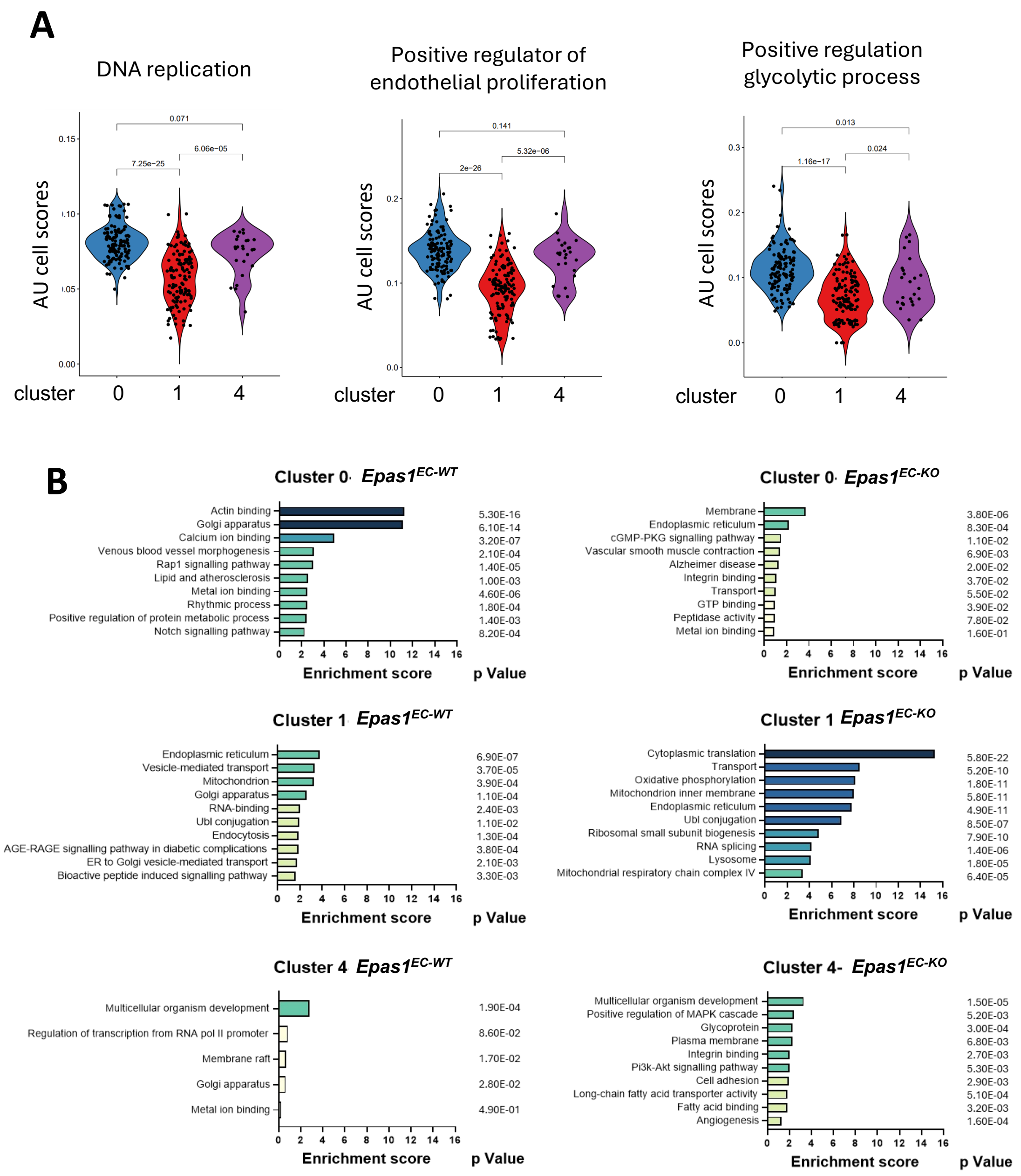

Figure S11. Functional annotation of scRNA-seq.
Epas1$^{EC\text{-}KO}$ mice and littermate controls lacking Cre (*Epas1*$^{EC\text{-}WT}$) were injected with tamoxifen aged 6 weeks and analysed 2 weeks later. Aortas from *Epas1*$^{EC\text{-}KO}$ and *Epas1*$^{EC\text{-}WT}$ littermate control mice were processed to generate single cells and then analysed by FACS of CD31^{+} CD45^{-} cells coupled to scRNAseq. Clusters were identified using unbiased hierarchical clustering. (A) Genes representing the GO terms 'DNA replication', 'Positive regulator of endothelial proliferation' and 'Positive regulation glycolytic process' were measured in each cell and are presented as violin plots as an average for clusters 0, 1 and 4. (B) Enrichment scores of enriched GO pathways for cells from *Epas1*$^{EC\text{-}KO}$ or *Epas1*$^{EC\text{-}WT}$ in clusters 0, 1 and 4. Differences in means were analysed using the Wilcoxon Rank Sum Test and p-values are annotated.

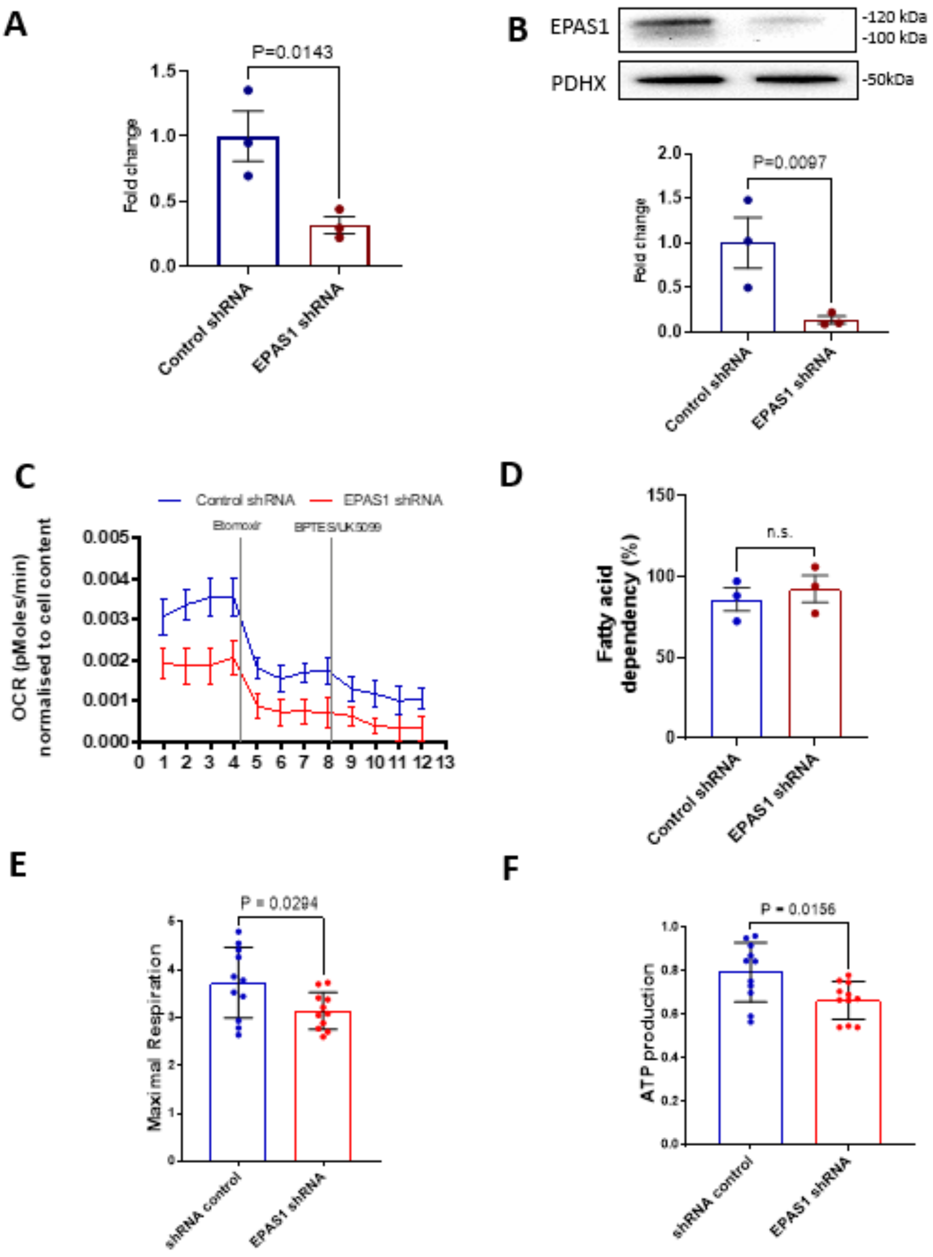

Figure S12. Endothelial EPAS1 regulates fatty acid metabolism.
(A, B) Validation of gene silencing. PAEC were treated with shRNA targeting *EPAS1* (N=3) or with scrambled control (N=3) and exposed to LOSS for 72 h using the orbital system. Expression levels of target genes were quantified by (A) qRT-PCR (n=3) or (B) immunoblotting (n=3). (C, D) Influence of *EPAS1* on bioenergetics profiles. PAEC were treated with shRNA targeting *EPAS1* (N=3) or with scrambled control (N=3) and then exposed to LOSS for 72 h using the orbital system. Basal oxygen consumption rates (OCR) were measured prior to addition of Etomoxir to assess contribution of fatty acid β-oxidation, and addition of UK5099 and BPTES to assess contribution of glycolysis and glutamate. (C) Kinetic profiles. Mean OCRs were calculated and are shown +/- standard errors. (D) Fatty acid dependency was calculated and mean values are shown +/- standard errors. Each data points represents a biological replicate. (E) Mitostress assay was performed to measure mitochondrial respiration capacity via inhibitions of complex V, I and III. Maximal respiration was calculated and mean values are shown +/- standard errors (N=11). (G) ATP production was calculated and mean values are shown +/- standard errors (N=11). Each datapoint represents a technical replicate in E and F. Differences between means were analysed using a Mann-Whitney U test (A,B,D) or at-test (E,F).

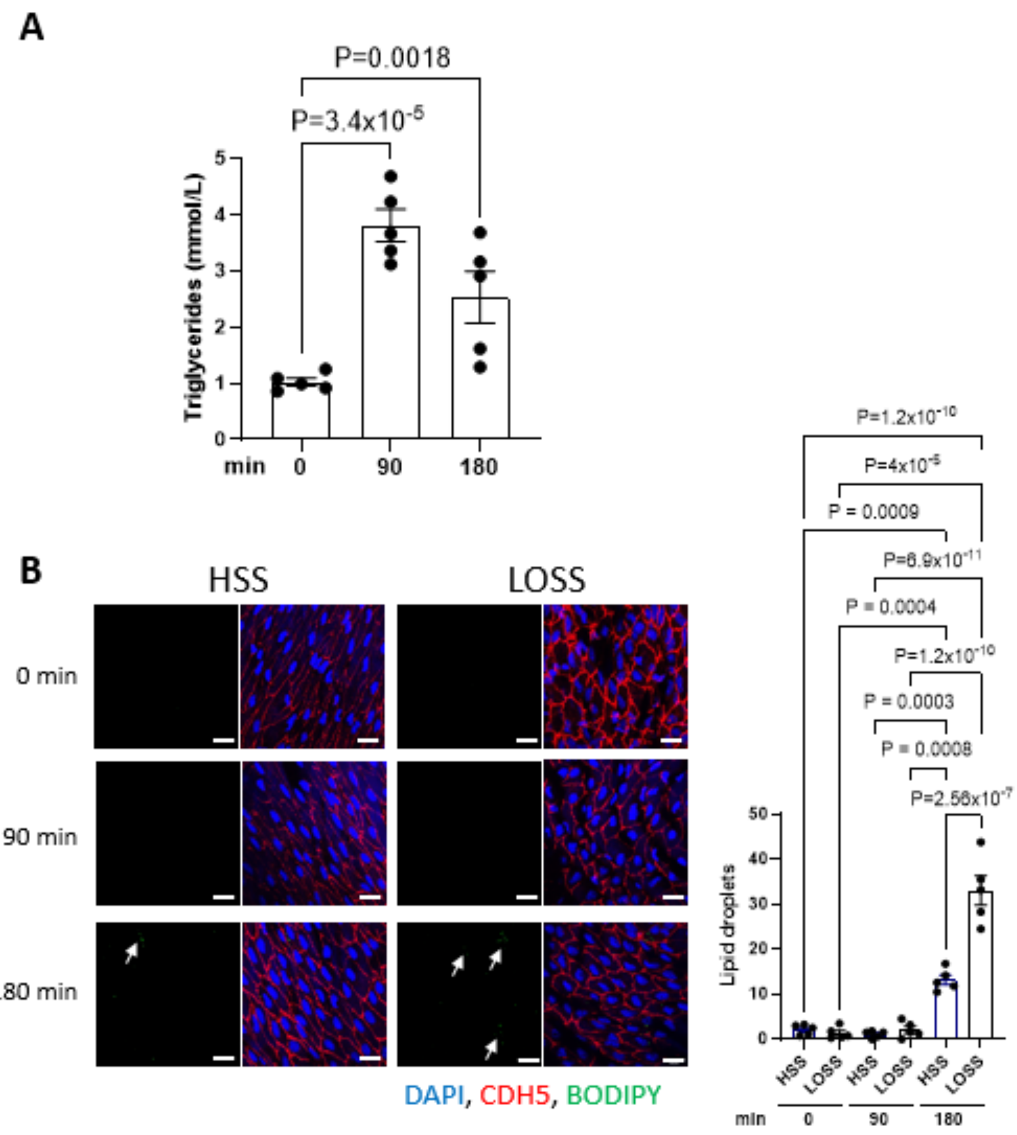

Figure S13. Lipid droplets accumulate in EC at an atheroprone region.
C57BL/6J mice aged 9 weeks were fasted for 8 h and then exposed to 10 ml/kg body weight olive oil by oral gavage. (A) Plasma triglyceride levels were quantified (N=5 per group). (B) Lipid droplets were quantified in EC of aortic arches by *en face* staining using BODIPY 493/503 (green). Endothelium was co-stained (CDH5; red) and nuclei detected using DAPI (blue). Scale bar 20µm.The number of lipid droplets identified in 10 fields of view was quantified for each animal and mean values +/- standard deviations are shown. Each data point represents an animal. Differences between means were analysed using a Kurskal-Wallis test (A) or an aligned ranked transform ANOVA (B).

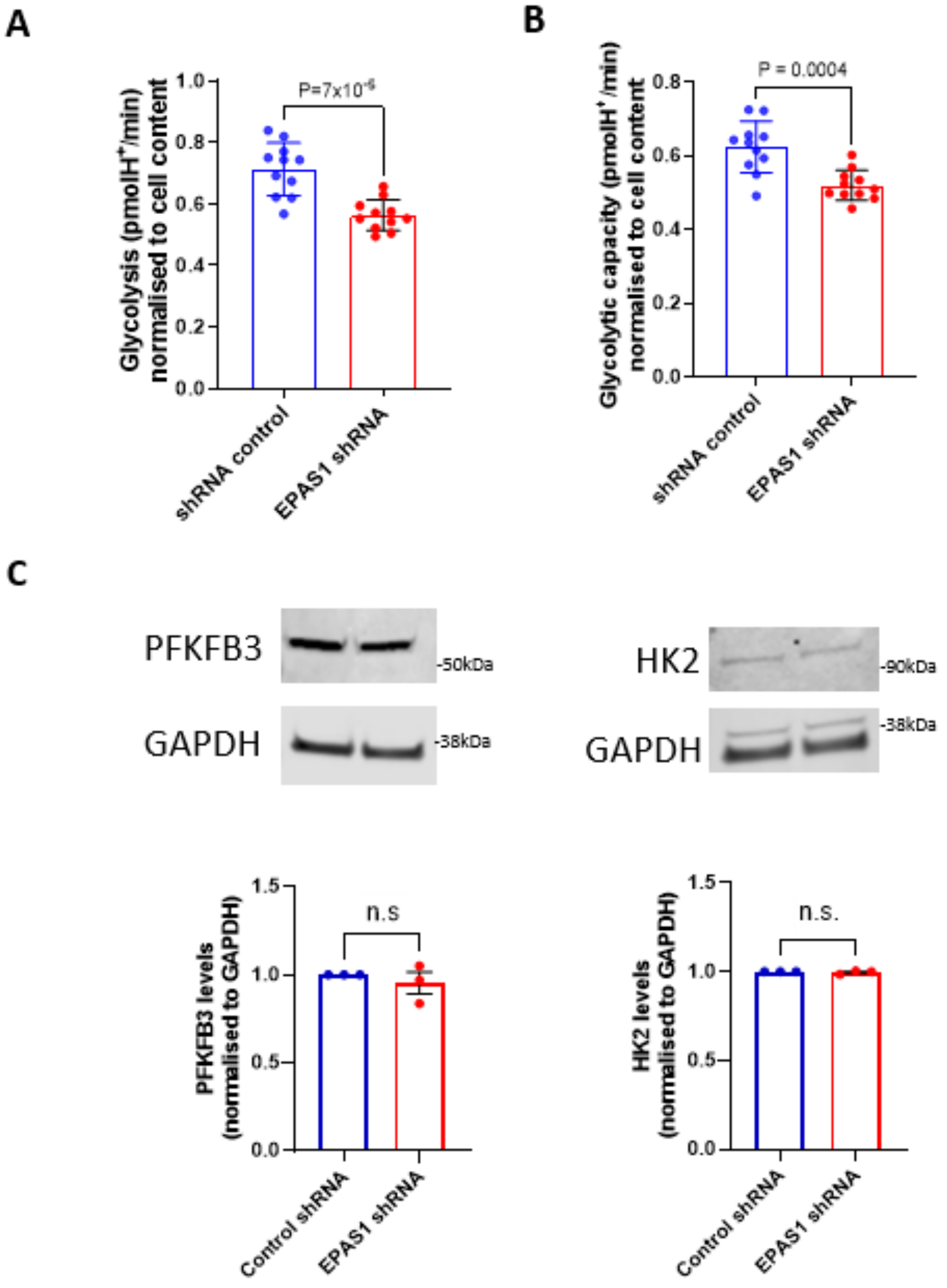

Figure S14. Endothelial EPAS1 does not regulate glycolysis.
PAEC were treated with shRNA targeting *EPAS1* or with scrambled control (N=3 per group) and then exposed to LOSS for 72 h using the orbital system. (A-C) Basal ECAR was assessed under glucose starvation to then investigate contribution of glycolysis after addition of glucose, followed by addition of oligomycin to assess glycolytic capacity. (A) Glycolysis and glycolytic capacity (B) were calculated and are shown +/- standard errors. (A, B) Each datapoint represents a technical replicate. (C) Protein levels of PFKFB3 and HK2 were quantified by immunoblotting. Representative images and mean values normalized to the level of GAPDH are shown. Each data point represents a biological replicate. Differences between means were analysed using a t-test (A,B) or a Mann-Whitney U test (C).

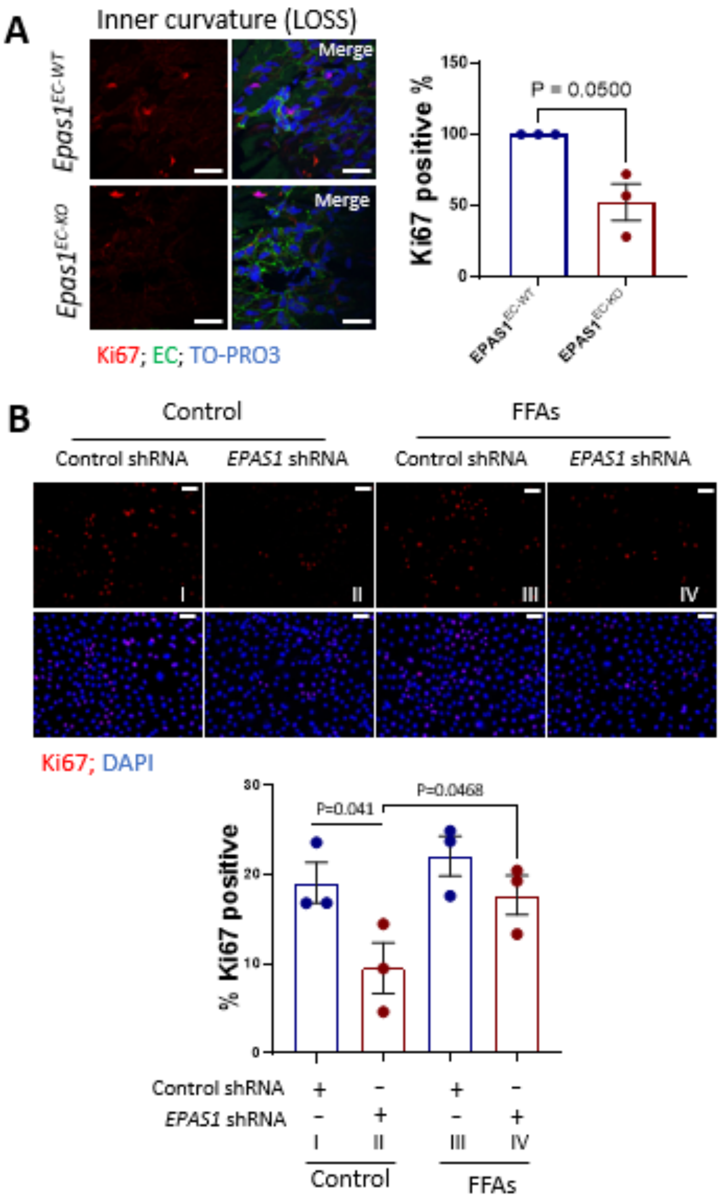

Figure S15. EPAS1 controls endothelial proliferation at atheroprone regions.
(A) *Epas1*$^{EC\text{-}KO}$ mice and littermate controls lacking Cre (*Epas1*$^{EC\text{-}WT}$) were injected with tamoxifen aged 6 weeks and analysed 2 weeks later (N=3 per group). Aortic EC were stained *en face* using anti-Ki67 antibodies (red) and fluorescence was quantified at a LOSS region. Endothelium was co-stained (EC; green) and nuclei detected using TO-PRO-3 (blue). Each data point represents an animal. Scale bar 50μm. (B) PAEC were treated with shRNA targeting *EPAS1* or with a scrambled control and then exposed to LOSS for 72 h using the orbital shaker system (N=3 per group). Some cultures were also exposed to exogenous OA. Protein levels of Ki67 were quantified by immunofluorescence staining with co-staining of nuclei using DAPI (n=3). The proportion of Ki67-positive cells was analysed and mean values +/- standard errors are shown. Each data point represents an animal. Differences between means were analyzed using a Mann-Whitney U test (A) or an aligned ranked transform ANOVA(B).

Table S1. qPCR primers

Species	Target	Forward Sequence	Reverse Sequence
Human	HPRT	TTGGTCAGGCAGTATAATCC	GGGCATATCCTACAACAAAC
Human	EPAS1	GCTTCCTGCGAACACACAAG	GGTCACCACGGCAATGAAAC
Human	PHD3	AACTGAATCTGCCCTCACTGAAG	ATAATTCAGGAACCGTTACTAAAATGA
Human	PHD2	TGAGCAGCATGGACGACCTGAT	CGTACATAACCCGTTCCATTGCC
Pig	B2M	GGTTCAGGTTTACTCACGCCAC	CTTAACTATCTTGGGCTTATCG
Pig	EPAS1	CAGGGGACGGTCATCTACAA	TCTCAATCTCACTCAGGACGT
Pig	LIPG	TGCAACAGCCAAAACCTTCT	TGTCCCACTTTCCTCGTGTT
Pig	CD36	CTGGTGCTGTCATTGGAGCAGT	CTGTCTGTAAACTTCCGTGCCTGTT
Mouse	HPRT	AGTCCCAGCGTCGTGATTAG	TCTCGAGCAAGTCTTTCAGTCC
Mouse	EPAS1	CAGAGCTGAGGAAGGAGAAATC	GCCAACTCATAGAAGACCTCC
Mouse	CDH5	TCTTGCCAGCAAACTCTCCT	TTGGAATCAAATGCACATCG
Mouse	ACTA2	CATCATGCGTCTGGACTTGG	AATCTCACGCTCGGCAGTAG

Table S2. Details of antibodies used for staining and western blotting.

Antibody target	Brand	Catalogue number	Concentration/dilution
Rabbit IgG	Millipore	12-370	6.6 µg/ml
Rat IgG	Biolegend	400401	10 µg/ml
Mouse IgG	Santa Cruz	Sc-2025	5µg/ml
Goat IgG	R&D	AB-108-C	10 µg/ml
EPAS1/HIF2-α	Abcam	ab199	6.6 µg/ml
PECAM-1 conjugated AF488	Biolegend	MEC13.3 (102501)	10 µg/ml
PHD2	Abcam	ab4561	5µg/ml
EPAS1/HIF2-α (microvasculature)	Novus Biological	NB100-122	5µg/ml
CDH5	BD Pharmingen	555289	5µg/ml
NRF2	Santa Cruz	sc13032	0.2 µg/ml
CD36	R&D	AF2519	10 µg/ml
LIPG	Abcam	ab24447	10 µg/ml
HIF1-α	Thermo Fisher Scientific	MA1-516	5µg/ml
Ki67	Thermo Fisher Scientific	14/5698/82	10 µg/ml
GAPDH	Millipore	CB1001	0.1 µg/ml
Alpha-tubulin	Cell Signaling	3873	0.02 µg/ml
PFKFB3	Cell Signaling Technology	13123S	1 µg/ml
HK2	Cell Signaling Technology	8337T	1 µg/ml
Picro Sirius Red Stain Kit	Abcam	ab150681	
Goat anti-rabbit conjugated AF568	Thermo Fisher Scientific	A-11036	6.6-10 µg/ml
Goat anti-rat conjugated AF568	Thermo Fisher Scientific	A-11077	10 µg/ml
Goat anti-rabbit AF488	Thermo Fisher Scientific	A-11034	10 µg/ml
Goat anti-Mouse AF647	Abcam	A-21235	3.3 µg/ml
Donkey anti-goat AF568	Thermo Fisher Scientific	A11057	10 µg/ml
Donkey anti-rat AF488	Thermo Fisher Scientific	A21208	10 µg/ml
Goat anti-mouse HRP	DAKO	P0447	1:3000
Swine anti-Rabbit HRP	DAKO	P0399	1:3000
Goat anti-Rabbit Dylight 680	Thermo Fisher Scientific	35568	1:10000
Goat anti-Mouse Dylight 800	Thermo Fisher Scientific	SA5-10176	1:10000

Table S3. Clinical data for obese cohort

OBESE COHORT											
study ID	SEX	BMI	AGE	TG	CHOL	HDL	LDL	CAD	Hypertension	Dyslipidemia	Diab_prediab
9	F	38.2	32	0.7	6.5	1.2	4.9	0	0	1	0
16	F	41.2	62	3.7	6.6	1.4	4.6	0	1	1	1
19	F	37.3	27	1.1	4.1	1.2	2.3	0	1	0	1
20	F	36.6	42	1.6	5.9	1.3	3.8	0	1	1	0
28	F	38.7	30	2.6	5.9	1.1	3.7	0	0	1	0
44	F	38.2	26	NT	NT	NT	NT	0	0	0	0
59	F	36.9	23	2.8	5.6	0.9	3.7	0	0	1	0
60	F	36	30	1.7	6.8	1	5.1	0	0	1	1
35	M	36.7	41	1.5	3.6	0.8	2.3	0	1	1	1
61	M	36.9	36	1.8	4.6	1.3	2.4	0	0	0	0
63	M	38.2	37	2.2	5.6	0.9	3.8	0	0	0	0
76	M	41.5	36	1.9	6.4	1.2	4.9	0	0	0	0
24	M	45	22	1.1	2.8	0.8	1.7	0	0	0	0
49	M	35.2	26	2.7	7	1	5.3	0	1	1	0
70	M	38.4	50	2.4	7	1.2	5.1	0	0	1	0
