# Supplementary material for: EPAS1 Attenuates Atherosclerosis Initiation at Disturbed Flow Sites Through Endothelial Fatty Acid Uptake
Source: Circ Res. 2024 Sep 5;135(8):822–37. doi: 10.1161/CIRCRESAHA.123.324054 (PMC11424061; doi:10.1161/CIRCRESAHA.123.324054)
Supplement: Supplementary file 4 [file res-135-822-s004.pdf]

Major Resources Table

In order to allow validation and replication of experiments, all essential research materials listed in the Methods should be included in the Major Resources Table below. Authors are encouraged to use public repositories for protocols, data, code, and other materials and provide persistent identifiers and/or links to repositories when available. Authors may add or delete rows as needed.

Animals (in vivo studies)

Species	Vendor or Source	Background Strain	Sex	Persistent ID / URL
Mice (Lep0b/ob)	Jax laboratory	Black/6J	Both	https://www.jax.org/strain/000632
Mice (C57BL/6J)	Jax laboratory	Black/6J	Both	https://www.jax.org/strain/000664
Mice (C57BL/6N)	Charles River	Black/6N	Both	https://www.criver.com/products-services/find-model/c57bl6-mouse?region=3671

Genetically Modified Animals

	Species	Vendor or Source	Background Strain	Other Information	Persistent ID / URL
**Parent - Male**	*Cdh5*-CreER$^{T+/-}$	Donation from Prof. Ralf Adams	Black/6J		
**Parent - Female**	*HIF2-α*$^{fl/fl}$	Donation from Prof. Randal Johnson	Black/6J		

Antibodies

Target antigen	Vendor or Source	Catalog #	Working concentration	Lot # (preferred but not required)	Persistent ID / URL
Rabbit IgG	Millipore	12-370	6.6 µg/ml		https://www.merckmillipore.com/GB/en/product/Normal-Rabbit-IgG,MM_NF-12-370?ReferrerURL=https%3A%2F%2Fwww.google.com%2F
Rat IgG	Biolegend	400401	10 µg/ml		https://www.biolegend.com/en-gb/products/purified-rat-igg1-kappa-isotype-ctrl-1832
Mouse IgG	Santa Cruz	Sc-2025	5µg/ml		https://datasheets.scbt.com/sc-2025.pdf
Goat IgG	R&D	AB-108-C	10 µg/ml		https://www.rndsystems.com/products/normal-goat-igg-control_ab-108-c?gad_source=1&gclid=CjwKCAjwhvi0BhA4EiwAX25uj0rWEY5dZuUzcVubrgBAXEupfqrrok17Eqws0N8RulwecmVS8XuHkhoCZd0QAvD_BwE&gclsrc=aw.ds
EPAS1/ HIF2-α	Abcam	ab199	6.6 µg/ml		https://www.abcam.com/en-gb/search?facets.categoryType=Primary%20Antibodies&sorting=relevance&keywords=epas1+antibody&gad_source=1&gclid=CjwKCAjwhvi0BhA4EiwAX25uj3uACslvoUXENqHcwNRMOpe_naOoF62Efjaw1l_pS5mT8ake6dFRqRoCICAQAvD_BwE&gclsrc=aw.ds
PECAM-1 conjugated AF488	Biolegend	MEC 13.3 (102501)	10 µg/ml		https://www.biolegend.com/fr-ch/products/purified-anti-mouse-cd31-antibody-380

DOI [to be added]

PHD2	Abcam	ab4561	5µg/ml		https://www.citeab.com/antibodies/746147-ab4561-anti-phd2-prolyl-hydroxylase-antibody
EPAS1/ HIF2-α (microvasculature)	Novus Biological	NB100-122	5µg/ml		https://www.novusbio.com/products/hif-2-alpha-epas1-antibody_nb100-122
CDH5	BD Pharmingen	555289	5µg/ml		https://www.bdbiosciences.com/en-au/products/reagents/functional-cell-based-reagents/purified-rat-anti-mouse-cd144.555289
NRF2	Santa Cruz	sc13032	0.2 µg/ml		https://www.scbt.com/p/nrf2-antibody-h-300
CD36	R&D	AF2519	10 µg/ml		https://www.rndsystems.com/products/mouse-cd36-sr-b3-antibody_af2519
LIPG	Abcam	ab24447	10 µg/ml		https://www.abcam.com/en-gb
HIF1-α	Thermo Fisher Scientific	MA1-516	5µg/ml		https://www.thermofisher.com/antibody/product/HIF1A-Antibody-clone-mgc3-Monoclonal/MA1-516
Ki67	Thermo Fisher Scientific	14/5698/82	10 µg/ml		https://www.thermofisher.com/antibody/product/Ki-67-Antibody-clone-SolA15-Monoclonal/14-5698-82
GAPDH	Millipore	CB1001	0.1 µg/ml		https://www.sigmaaldrich.com/GB/en/product/mm/cb1001
Alpha-tubulin	Cell Signaling	3873	0.02 µg/ml		https://www.cellsignal.com/products/primary-antibodies/a-tubulin-dm1a-mouse-mab/3873
PFKFB3	Cell Signaling Technology	13123S	1 µg/ml		https://www.cellsignal.com/products/primary-antibodies/pfkfb3-d7h4q-rabbit-mab/13123
HK2	Cell Signaling Technology	8337T	1 µg/ml		https://www.cellsignal.com/products/primary-antibodies/hexokinase-ii-c64g5-rabbit-mab/2867
Picro Sirius Red Stain Kit	Abcam	ab150681			https://www.abcam.com/ps/products/150/ab150681/documents/Picro-Sirius-Red-Staining-protocol-book-v3e-ab150681%20(website).pdf

DOI [to be added]

Goat anti-rabbit conjugated AF568	Thermo Fisher Scientific	A-11036	6.6-10 µg/ml		https://www.thermofisher.com/antibody/product/Goat-anti-Rabbit-IgG-H-L-Highly-Cross-Adsorbed-Secondary-Antibody-Polyclonal/A-11036
Goat anti-rat conjugated AF568	Thermo Fisher Scientific	A-11077	10 µg/ml		https://www.thermofisher.com/antibody/product/Goat-anti-Rat-IgG-H-L-Cross-Adsorbed-Secondary-Antibody-Polyclonal/A-11077
Goat anti-rabbit AF488	Thermo Fisher Scientific	A-11034	10 µg/ml		https://www.thermofisher.com/antibody/product/Goat-anti-Rabbit-IgG-H-L-Highly-Cross-Adsorbed-Secondary-Antibody-Polyclonal/A-11034
Goat anti-Mouse AF647	Abcam	A-21235	3.3 µg/ml		https://www.thermofisher.com/antibody/product/Goat-anti-Mouse-IgG-H-L-Cross-Adsorbed-Secondary-Antibody-Polyclonal/A-21235
Donkey anti-goat AF568	Thermo Fisher Scientific	A11057	10 µg/ml		https://www.thermofisher.com/antibody/product/Donkey-anti-Goat-IgG-H-L-Cross-Adsorbed-Secondary-Antibody-Polyclonal/A-11057
Donkey anti-rat AF488	Thermo Fisher Scientific	A21208	10 µg/ml		https://www.thermofisher.com/antibody/product/Donkey-anti-Rat-IgG-H-L-Highly-Cross-Adsorbed-Secondary-Antibody-Polyclonal/A-21208
Goat anti-mouse HRP	DAKO	P0447	1:3000		https://www.agilent.com/store/productDetail.jsp?catalogId=P044701-2
Swine anti-Rabbit HRP	DAKO	P0399	1:3000		https://www.agilent.com/en/product/specific-proteins/elisa-kits-accessories/swine-anti-rabbit-immunoglobulins-hrp-affinity-isolated-2717118
Goat anti-Rabbit Dylight 680	Thermo Fisher Scientific	35568	1:10000		https://www.thermofisher.com/antibody/product/Goat-anti-Rabbit-IgG-H-L-Secondary-Antibody-Polyclonal/35568
Goat anti-Mouse	Thermo Fisher	SA5-10176	1:10000		https://www.thermofisher.com/antibody/product/Goat-anti-Mouse-IgG-H-L-Cross-Adsorbed-Secondary-Antibody-Polyclonal/SA5-10176

DOI [to be added]

Dylight 800	Scientific				

DNA/cDNA Clones

Clone Name	Sequence	Source / Repository	Persistent ID / URL

Cultured Cells

Name	Vendor or Source	Sex (F, M, or unknown)	Persistent ID / URL
Porcine Aortic Endothelia Cells (PAEC)	Isolated from porcine aorta	unknown	

Data & Code Availability

Description	Source / Repository	Persistent ID / URL

Other

Description	Source / Repository	Persistent ID / URL
Sulforaphane	Cayman Chemical	https://www.caymanchem.com/product/10496/sulforaphane
Streptozotocin	Sigma Aldrich	https://www.sigmaaldrich.com/IT/it/product/sigma/s0130
Oleic Acid	Sigma Aldrich	https://www.sigmaaldrich.com/IT/it/product/sigma/o3008
Bovine Serum Albumin	Sigma Aldrich	https://www.sigmaaldrich.com/IT/it/product/sigma/a1595
Palmitate	Sigma Aldrich	https://www.sigmaaldrich.com/IT/it/product/sigma/p5585
Lentiviral pool targeting EPAS1 (V3SH7590)_Clone ID: V3SVHSHC_8578211,	Dharmacon	https://horizondiscovery.com/en/gene-modulation/knockdown/shrna/products/smartvector-lentiviral-shrna?nodeid=entrezgene-2034#supporting-data

DOI [to be added]

V3SVHSHC_5189144, V3SVHSHC_8945039		
Lentiviral Non-targeting control	Dharmacon	https://horizondiscovery.com/en/gene-modulation/knockdown/controls/products/smartvector-lentiviral-controls#resources
Tamoxifen	Sigma Aldrich	https://www.sigmaaldrich.com/IT/it/product/sigma/t5648
Corn Oil	Sigma Aldrich	https://www.sigmaaldrich.com/IT/it/product/sigma/c8267
Etomoxir	MedChemExpress	https://www.medchemexpress.com/Etomoxir.html
Rotenone	Tocris	https://www.tocris.com/products/rotenone_3616?gad_source=1&gbraid=0AAAAADRLDxQkf_TqJeObhoGEK9K30qI5N&gclid=EAIaIQobChMI44CCtOC7hwMVcYxQBh0XNyNyEAAYASAAEgLHqvD_BwE&gclsrc=aw.ds
Antimycin A	Santa Cruz	https://www.scbt.com/p/antimycin-a-1397-94-0
BPTES	Sigma Aldrich	https://www.sigmaaldrich.com/IT/it/product/sigma/sml0601
UK5099	Cayman Chemical	https://www.caymanchem.com/product/16980/uk-5099
Oligomycin	Santa Cruz	https://www.scbt.com/p/oligomycin-1404-19-9
FCCP	Tocris	https://www.tocris.com/products/fccp_0453

DOI [to be added]
