# Supplementary material for: EPAS1 Attenuates Atherosclerosis Initiation at Disturbed Flow Sites Through Endothelial Fatty Acid Uptake
Source: Circ Res. 2024 Sep 5;135(8):822–37. doi: 10.1161/CIRCRESAHA.123.324054 (PMC11424061; doi:10.1161/CIRCRESAHA.123.324054)
Supplement: Supplementary file 5 [file res-135-822-s005.pdf]

Full unedited gel for Figure_1A

EPAS1 PDHX

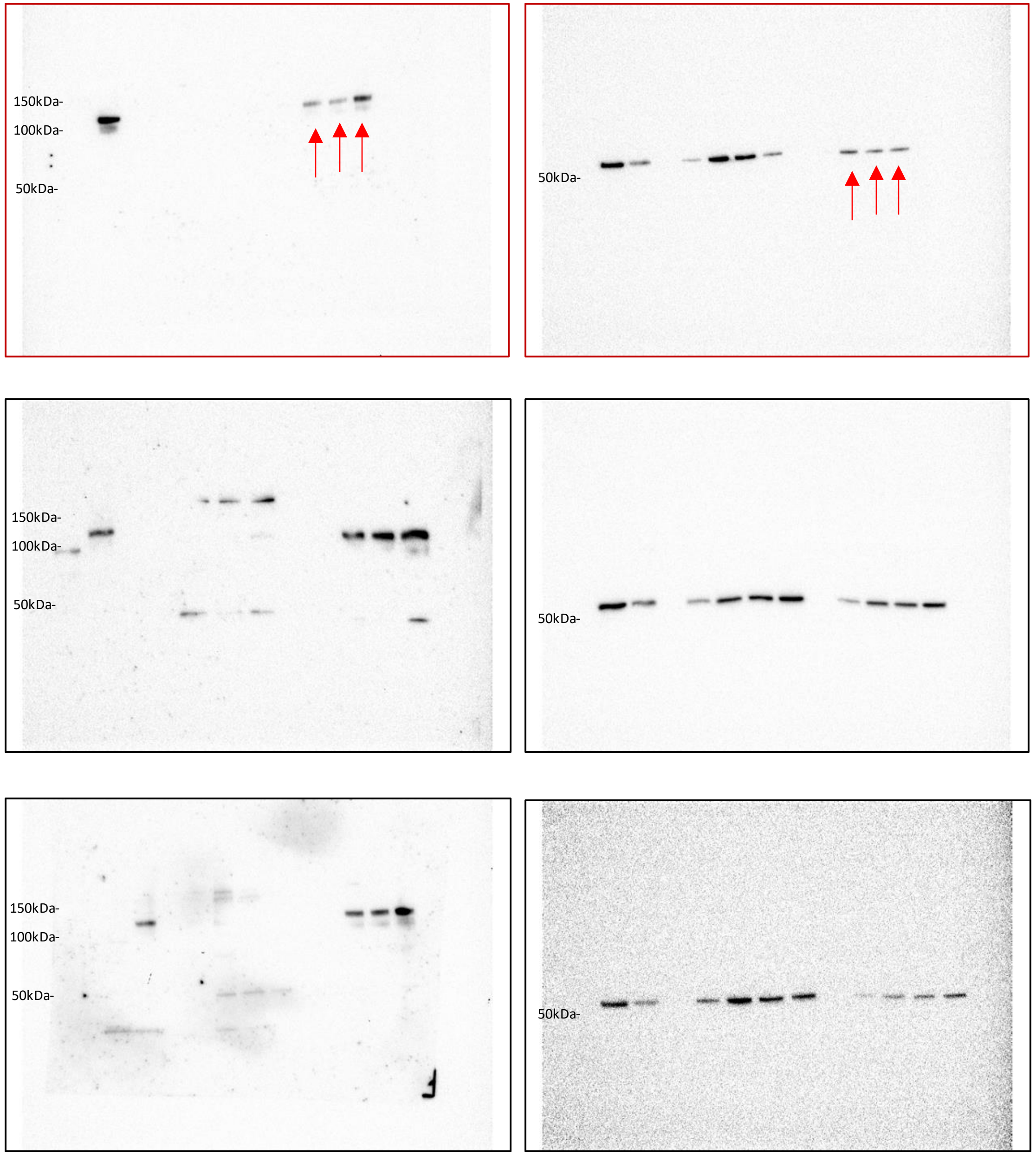

File name: WB24

File name: WB22

File name: WB21

PAEC exposed to High, Low or Low oscillatory shear stress for 72h on an ibidi flow system. Immunoblots were imaged using Biorad Chemiluminescence.

LADDER
Well2 = normoxia control (static)
Well 3= hypoxia control (static)
Well 4 = empty
Well 5= PAEC static
Well 6 = PAEC HSS
Well 7= PAEC LSS
Well 8= PAEC LOSS
Well 9 = empty
Well 10= PAEC static
Well 11= PAEC (HSS) + DMOG for collection
Well 12= PAEC (LSS) + DMOG for collection
Well 13= PAEC (LOSS) + DMOG for collection
Well 14-15=empty

Arrows indicating which lanes of the unedited gel correspond to those shown in the cropped blots within the manuscript.

Full unedited gel for Figure_1A

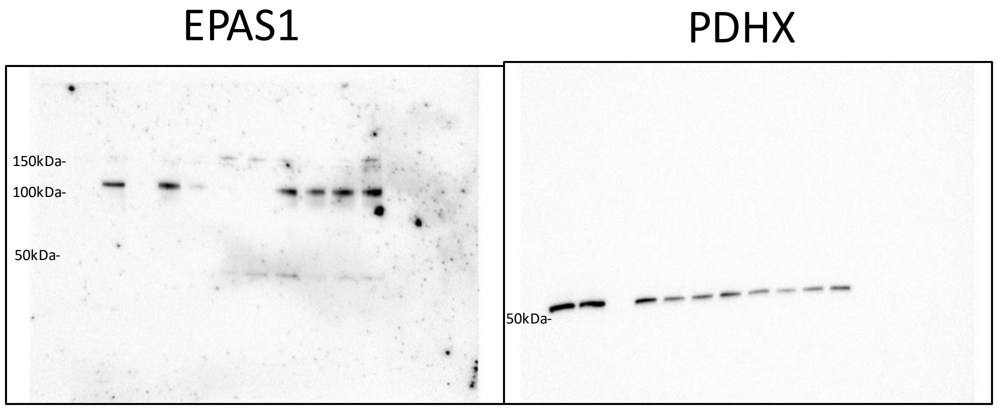

File name: WB27

PAEC exposed to High, Low or Low oscillatory shear stress for 72h on an ibidi flow system. Immunoblots were imaged using Biorad Chemiluminescence.

LADDER
Well2 = normoxia control (static)
Well 3= hypoxia control (static)
Well 4 = empty
Well 5= PAEC static
Well 6 = PAEC HSS
Well 7= PAEC LSS
Well 8= PAEC LOSS
Well 9 = empty
Well 10= PAEC static
Well 11= PAEC (HSS) + DMOG for collection
Well 12= PAEC (LSS) + DMOG for collection
Well 13= PAEC (LOSS) + DMOG for collection
Well 14-15=empty

Full unedited gel for Figure_ 5A

EPAS1

Alpha tubulin

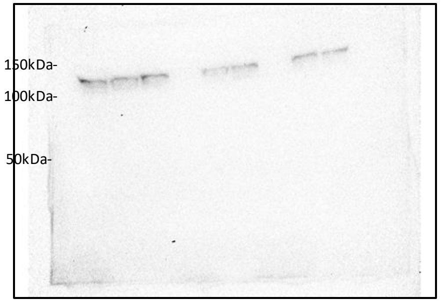

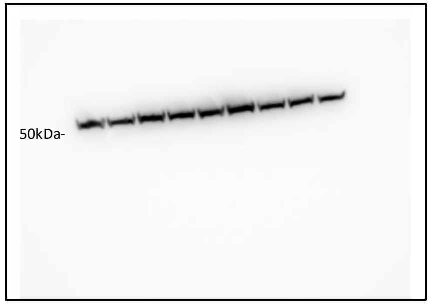

File name: WB21ICL

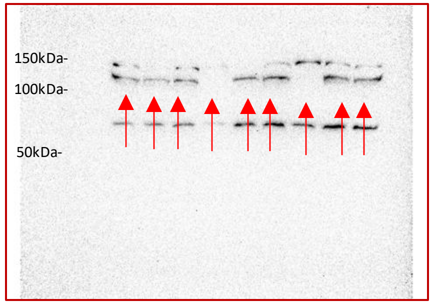

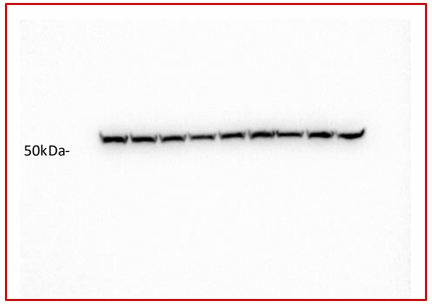

File name: WB22ICL

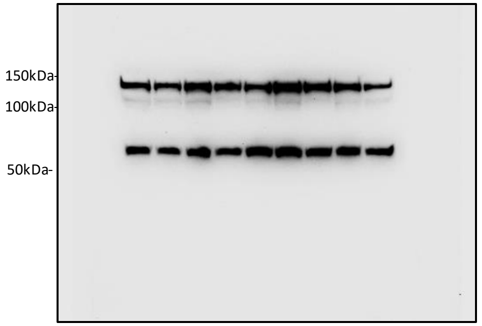

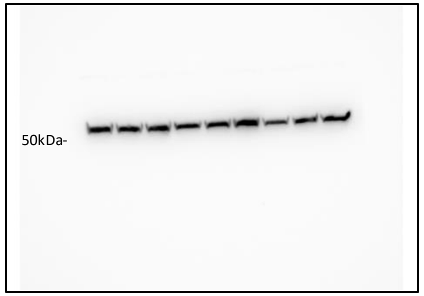

File name: WB24ICL

PAEC exposed to Low oscillatory shear stress for 72h and treated with Palmitate or oleic acid conjugated in BSA, Vehicle is BSA. With or without Sulforaphane or N-acetylcysteine treatment. Immunoblots were imaged using Biorad Chemiluminescence.

Well1 =Ladder
Well2 = Vehicle
Well 3= Sulforaphane
Well 4 = N-acetyl cysteine
Well 5= Oleic acid
Well 6 = Oleic acid+ Sulforaphane
Well 7= OA+N-acetyl cysteine
Well 8= PA
Well 9 = PA+Sulforaphane
Well 10= PA+N-acetyl cysteine

Full unedited gel for Figure_ 5A

Alpha tubulin

PAEC exposed to Low oscillatory shear stress for 72h and treated with Palmitate or oleic acid conjugated in BSA, Vehicle is BSA. With or without Sulforaphane or N-acetylcysteine treatment. Immunoblots were imaged using Odyssey DLx (Li-Cor).

Well1 =Ladder Dualcolour chameleon
Well2 = Vehicle
Well 3= Sulforaphane
Well 4 = N-acetyl cysteine
Well 5= Oleic acid
Well 6 = Oleic acid+ Sulforaphane
Well 7= OA+N-acetyl cysteine
Well 8= PA
Well 9 = PA+Sulforaphane
Well 10= PA+N-acetyl cysteine

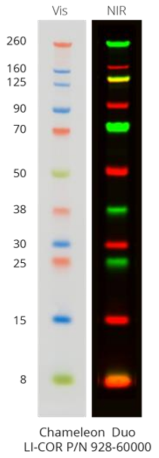

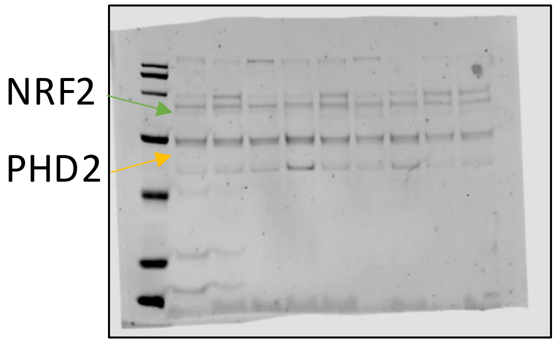

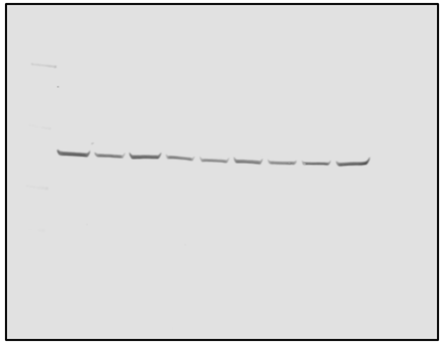

File name: WB10ICL

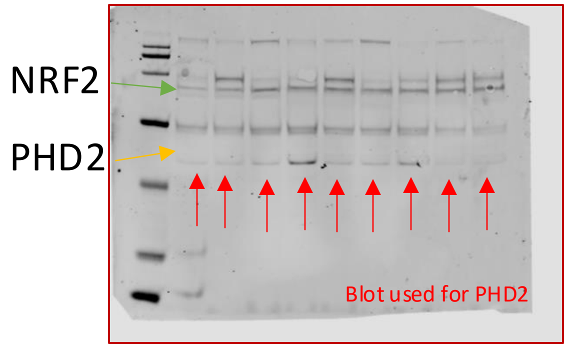

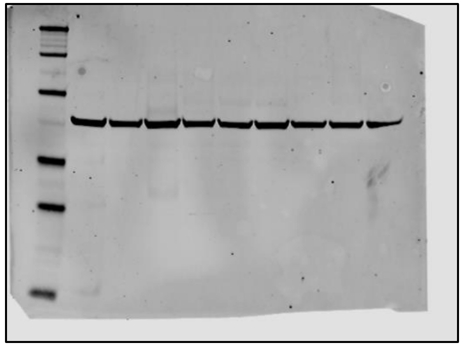

File name: WB13ICL

File name: WB7ICL for NRF2

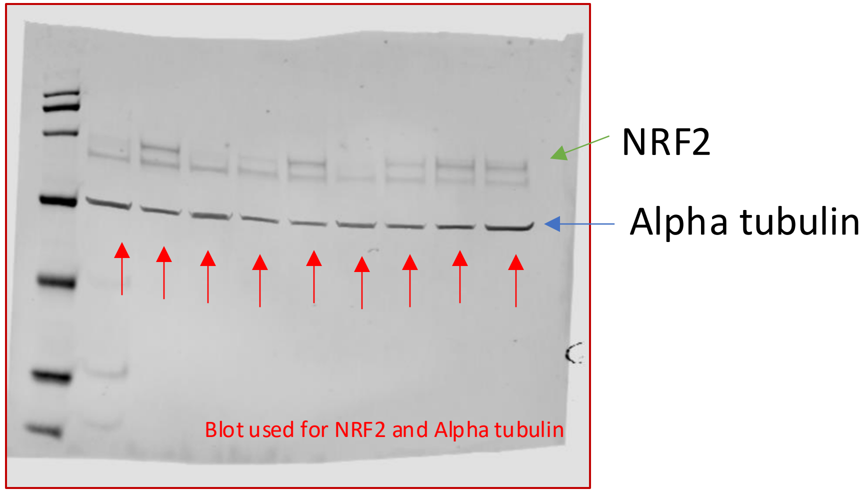

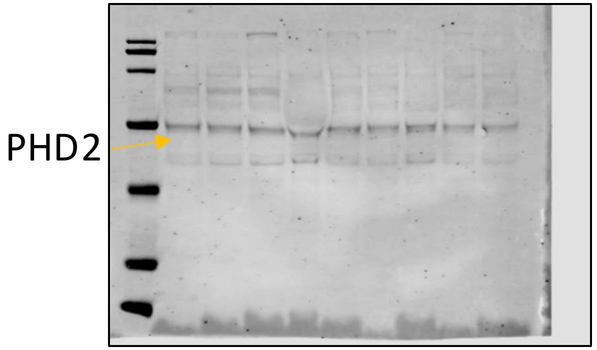

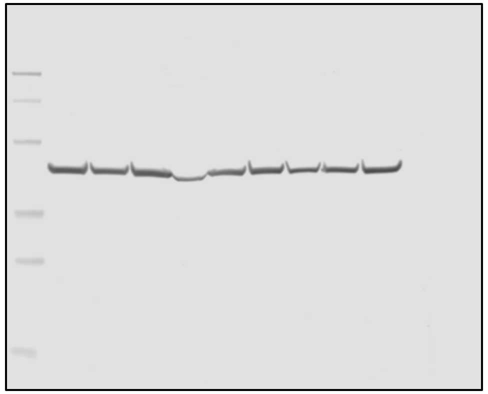

File name: WB14ICL for PHD2

Full unedited gel for Figure_ S12

EPAS1

PDHX

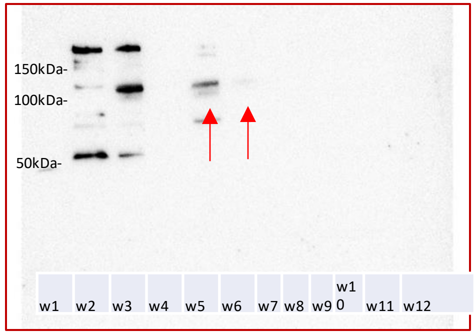

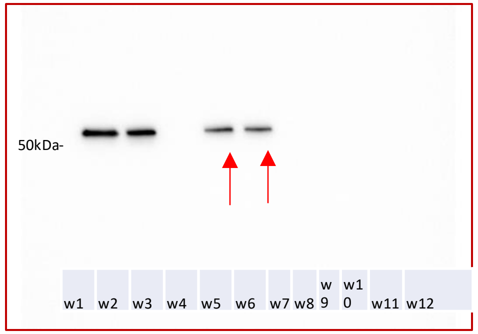

File name: WB41

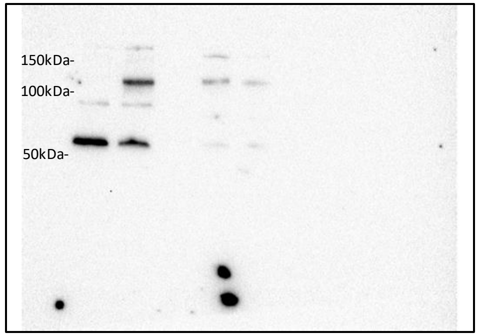

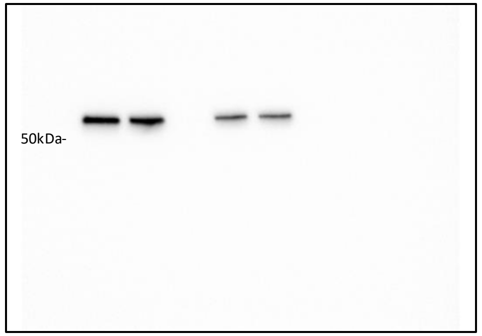

File name: WB42

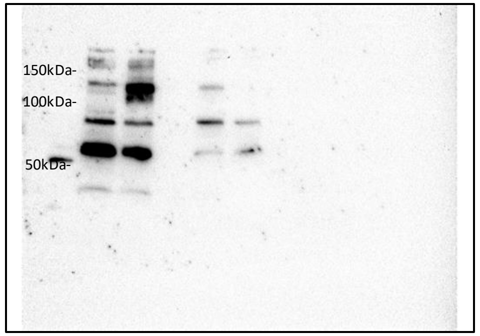

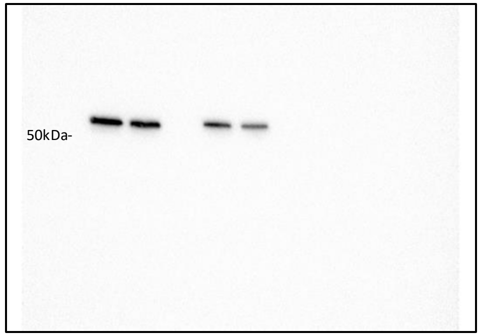

File name: WB43

PAEC treated with EPAS1 shRNA or control, and exposed to Low oscillatory shear stress for 72h. Immunoblots were imaged using Biorad Chemiluminescence.

Well1= ladder
Well 2= normoxia control (static)
Well 3= hypoxia control (static)
Well 4 = empty
Well 5= PAEC control treated cells (LOSS)
Well 6= PAEC shRNA treated cells (LOSS)
Well 7-12=empty

Full unedited gel for Figure_ S14

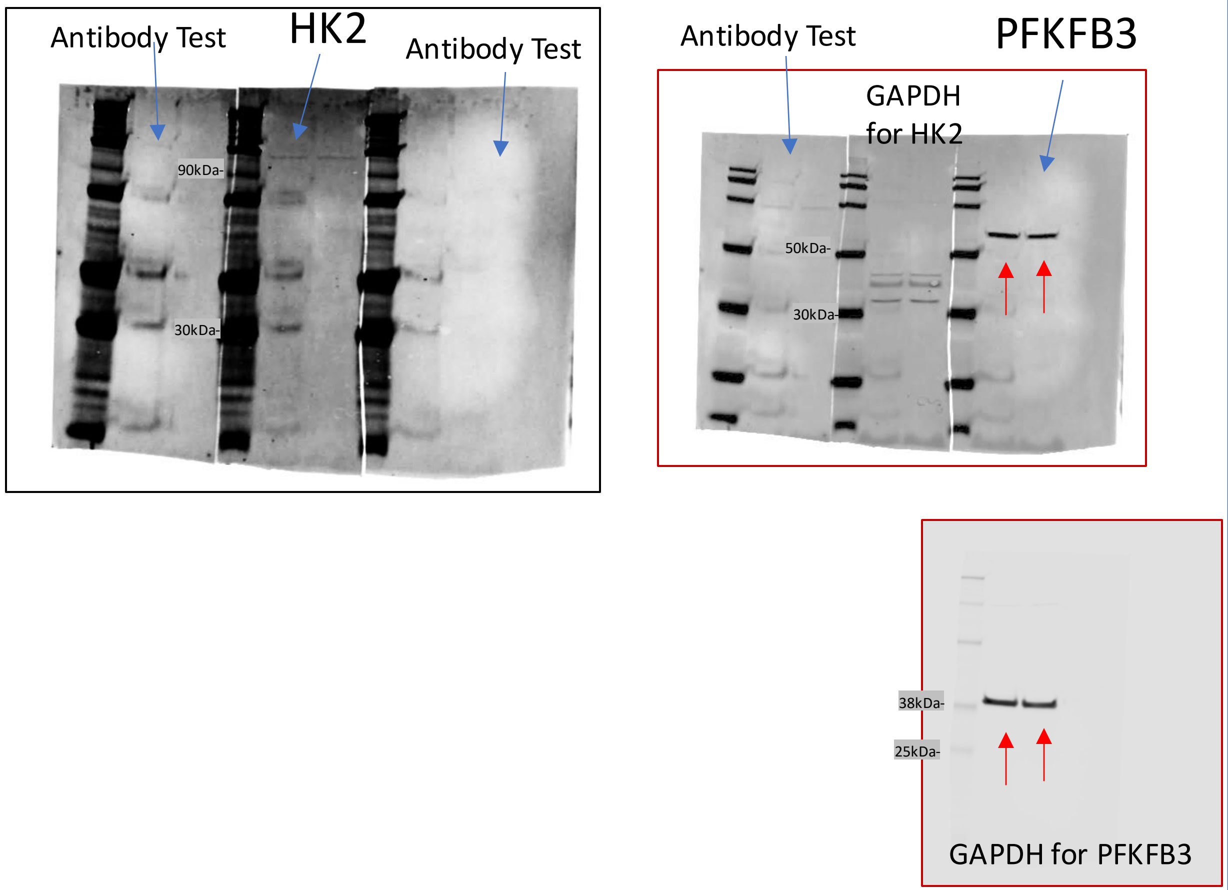

File name: #PAEC_WB1_ICL

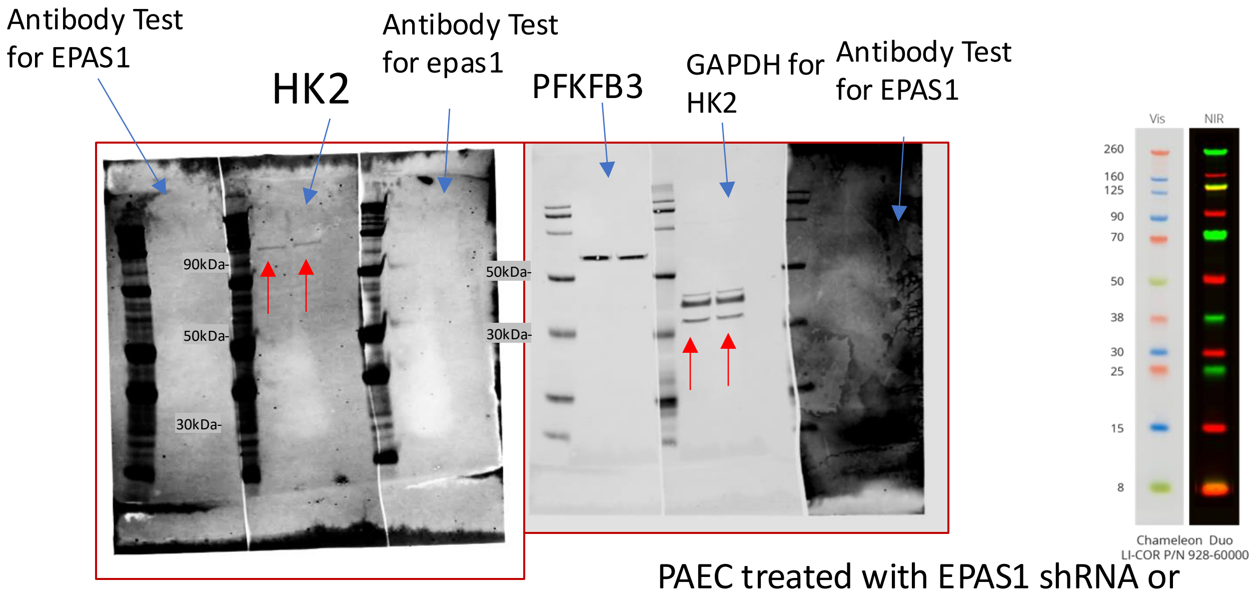

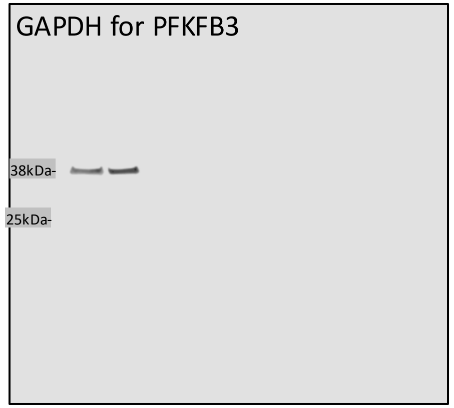

File name: #PAEC_WB3_ICL

PAEC treated with EPAS1 shRNA or control and exposed to Low oscillatory shear stress for 72h . Immunoblots were imaged using Odyssey DLx (Li-Cor).

Loading sequence for WB1 and 3

Well1 =Ladder

Well2 = Control shRNA

Well 3= EPAS1 shRNA

Well 4 = ladder

Well 5= Control shRNA

Well 6 = EPAS1 shRNA

Well 7= Ladder

Well 8= Control shRNA

Well 9 = EPAS1 shRNA

Well 10= empty

Full unedited gel for Figure_ S14

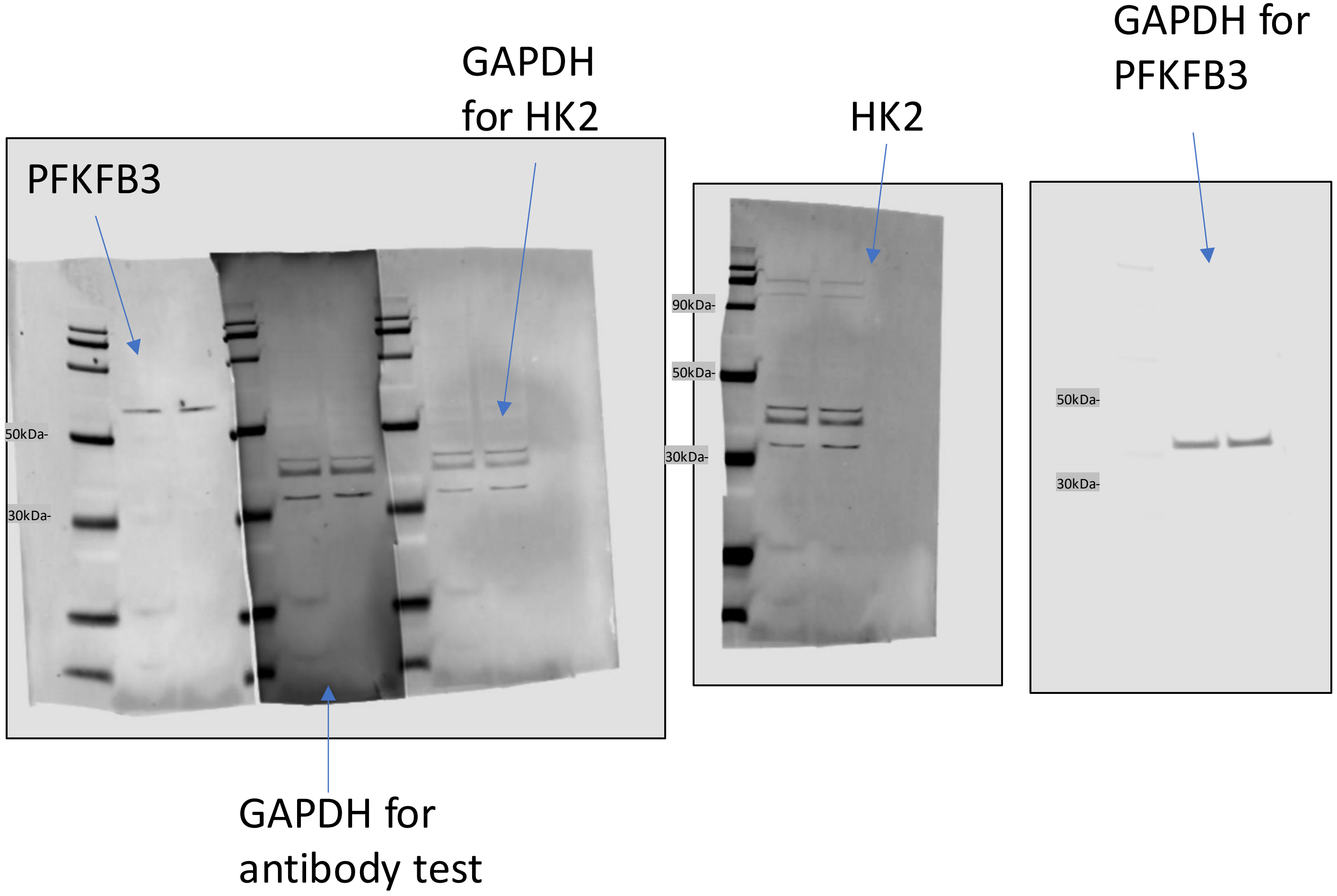

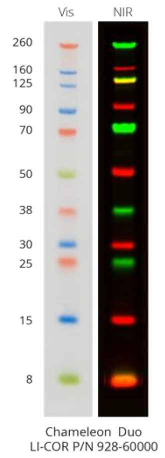

PAEC treated with EPAS1 shRNA or control and exposed to Low oscillatory shear stress for 72h . Immunoblots were imaged using Odyssey DLx (Li-Cor).

Loading sequence for WB5

Well1 =Ladder dual-colour chameleon

Well2 = Control shRNA

Well 3= EPAS1 shRNA

Well 4 = ladder

Well 5= Control shRNA

Well 6 = EPAS1 shRNA

Well 7= Ladder

Well 8= Control shRNA

Well 9 = EPAS1 shRNA

Well 10= empty

File name: #PAEC_WB5_ICL
