# Supplementary material for: EPAS1 Attenuates Atherosclerosis Initiation at Disturbed Flow Sites Through Endothelial Fatty Acid Uptake
Source: Circ Res. 2024 Sep 5;135(8):822–37. doi: 10.1161/CIRCRESAHA.123.324054 (PMC11424061; doi:10.1161/CIRCRESAHA.123.324054)
Supplement: Supplementary file 1 [file res-135-822-s001.pdf]

**Title: EPAS1 Attenuates Atherosclerosis Initiation at Disturbed Flow Sites Through Endothelial Fatty Acid Uptake**

In an effort to promote greater transparency in peer review, the authors and reviewers of this *Circulation Research* article have opted to post the original decision letter with reviewer comments to the authors and the authors' response to reviewers for each significant revision.

---

December 28, 2023

Dr. Paul C Evans
Queen Mary University of London
United Kingdom

RE: CIRCRES/2023/324054: EPAS1 Attenuates Atherosclerosis Initiation at Disturbed Flow Sites through Endothelial Fatty Acid Uptake

Dear Dr. Evans:

Your manuscript has been carefully evaluated by two external reviewers and the editors as an Original Research manuscript. Although of potential interest, the paper is not acceptable for publication in Circulation Research in its present form.

As you will gather from the reviews, the referees identified a number of substantive conceptual and methodological problems. The editors concur. Major issues include the numbers of animals/subjects/cells used in experiments to achieve robust and statistically significant conclusions, and the need for deeper mechanistic studies.

Given the extensive new data that would be required for a responsive revision, we would understand if you were to decide to submit the paper elsewhere. Nevertheless, the editors see this manuscript as potentially important and would be willing to evaluate a revised version if you feel that you can effectively address the reviewers' concerns and are willing to perform the new experiments required. The paper would be reviewed again, with no assurance of acceptance.

As detailed in the reviewers' critiques, a responsive revision would require a substantial amount of new data. The Editors strongly encourage you to adhere to the journal's Statistical Reporting Recommendations in your revision, which can be found here: https://www.ahajournals.org/statistical-recommendations.

To read the comments to authors from the reviewers, please see below.

Please note that revised and resubmitted manuscripts are not assured of publication, and that fewer than 15% of all papers submitted to Circulation Research are eventually published.

Our policy is to allow only one major revision. Our current guidelines allow authors 90 days to complete the revision. If you need more than 90 days to submit a revised paper, please notify the editorial office.

You are encouraged to log in and start the revision submission process as soon as possible, because all authors must complete disclosure and publication agreement forms BEFORE the revision can be submitted.

As corresponding author, you may start the revision process at any time and go to the Copyright & Licensing tab to contact coauthor(s) and send reminders. In addition, please review all revision submission tabs to ensure that you are prepared to provide the appropriate files, checklists, and other items when it is time to submit your revision.

We are piloting an integration with SciScore (https://www.sciscore.com) to provide authors automatically generated reports during revision submission containing a reproducibility score and tables on rigor adherence and key resources such as antibodies, experimental models, recombinant DNA, and software. You are also welcome to start the revision submission process at any time to receive your report. We strongly encourage you to use the provided report while revising your manuscript to improve the study's reproducibility and reporting quality.

If you choose to revise, please include a detailed response to each of the referees' and editors' comments, providing each comment verbatim in bold followed by your response and giving the exact page number(s), paragraph(s), and line number(s) where each revision was made. If you make substantive changes to the manuscript, please provide a clear description of what you did and where. If you insert important sentences, paragraphs, or sections in response to the comments, please also include them in your response. Please indicate clearly any deletions. Additionally, a marked up version of the revision with the changes highlighted or tracked should be uploaded as a supplemental file. Each page of the revised manuscript should be numbered in the top right corner, using your manuscript number followed by /R1 to denote a first revision.

Please ascertain that your revised manuscript adheres to the Instructions to Authors as they appear online at https://www.ahajournals.org/res/author-instructions. Please refer to the Instructions to Authors for further details regarding our policy on page limits, articles with extended print versions, and related costs. No such limits apply to the online supplementary information, which can include supporting data and/or expanded text to offset the limits on the print version. Such online supplementary information can be cited in the print version as appropriate.

We wish to thank you for having submitted this manuscript to Circulation Research.

Sincerely,

Jane E. Freedman, MD
Editor-in-Chief
Circulation Research
An American Heart Association Journal

****************************************************************************************

**REVIEWER COMMENTS TO AUTHORS:**

**Reviewer #1:**
The investigators demonstrate that endothelial cell (EC) EPAS1 contributes to the prevention of atherosclerosis in the arterial regions exposed to low/oscillatory shear stress. Using both in vitro and in vivo models, Priri et al. showed that EPAS1 attenuates atherosclerosis initiation by maintaining EC proliferation via fatty acid uptake and lipid metabolism, whereas the reduction of EPAS1 in an PHD3-dependent manner

leads to atherosclerosis in the hyperlipidemia milieu/obesity. While this study is novel and significant, there are several major and minor concerns prior to publication.

Major:
1. The previous literature has established EPAS1 activation in chronic hypoxia; however, please discuss the relationship between LOSS and hypoxia in endothelial cells.
2. The authors conclude that activation of fatty acid metabolism by EPAS1 in aortic inner curvature prevents atherosclerosis. Please explain how EC fatty acid metabolism contributes to endothelial cell repair. Please provide evidence to demonstrate differential EC metabolism between inner vs. outer curvature. This difference would strengthen the the hyperlipidemia responses.
3. The authors show that oscillatory hemodynamics is necessary to regulate EPAS1 in vitro and in vivo. However, microvascular ECs in adipose tissue are not exposed to oscillatory hemodynamics. Please explain this difference.
4. The authors measured plasma EPAS1 expression levels in human subjects. However, it is unclear whether plasma EPAS1 levels can be used as a surrogate for EPAS1that is released from ECs?
5. If EPAS1 affects endothelial cells in the microvasculature and resistance arteries, it would be interesting to show the blood pressure in human subjects. From this perspective, examining the EPAS1 levels in the resistance artery or microvascular ECs and blood pressure in Lepob/ob mice would be insightful.
6. The link between hyperlipidemia and EPAS1 requires further explanation
7. The study should explore the different mechanisms in HSS and LOSS regions that both exhibit atherosclerosis protective phenotypes.
8. Streptozotocin (STZ) is also known to cause eNOS uncoupling and ROS production, leading to endothelial cell (EC) dysfunction. The specific effects of STZ on EC dysfunction and atherosclerosis need to be articulated.
9. The scRNAseq results include only a limited number of ECs, and these cells are mixed between groups. Please provide statistical significance with a small sample size.
10. Oscillatory shear stress promotes glycolysis in ECs via PFKFB3. In contrast, the authors propose that in LOSS conditions, EPAS1 stimulates fatty acid metabolism, leading to EC proliferation and, subsequently, protection against atherosclerosis. The authors may need address how EC glycolysis is affected under LOSS and explain the protective role of proliferative ECs in this context.
11. Please elaborate as to why proliferative ECs have protective effects.
12. The authors used oleic acid as a free fatty acid model for hyperlipidemia. Palmitic acid may better represent hyperlipidemic conditions.

Minor:
1. In the abstract, where it mentions "Obese mice... correlating...", how did the authors determine this correlation?
2. In the introduction, is there evidence to show that HIF-2A does not regulate angiogenesis, unlike HIF-1A?
3. Please provide more details on how blood pressure was measured using the CODA system.
4. In the Seahorse experiment, the authors seemed to measure beta-oxidation to OCR levels using Etomoxir and BPETS+UK5099. However, for a comprehensive assessment of the respiratory phenotype in EPAS1 shRNA ECs, establishing a standard OCR curve initially, perhaps with agents such as Oligomycin, FCCP, Rotenone/Antimycin A, would be beneficial. Also, it would be advisable to modify the x-axis in Figure S8C to reflect time instead of measurement.
5. There appears to be no expression of EPAS1 in the outer curvature (HSS). It would be appropriate to replace the representative images with those that align with the bar graph and findings in Figure 1A.

6. The effects of HFD on body weight, glycaemia, and TG are well-known. Figure 2A-C would be better placed in the supplementary.
7. The expression level of EPAS1 in Figure 2E is significantly higher than in Figure 1C, even for the chow diet group. Could this difference be attributed to age or body weight variations?
8. Figures 2G-H and 3A-B would be better suited for the supplementary.
9. There seems to be some confusion regarding the definition of LOSS in the discussion; it was referred as laminar oscillatory shear stress.
10. For better readership, consistent use of nomenclature throughout the paper.
11. The immunofluorescence staining in Figure S4A is unclear. Please provide Western Blot (WB) data.
12. Providing a table detailing the characteristics of human subjects would be necessary.
13. Please discuss more LIPG, CD36, and PHD3 when the authors first introduce them on page 18, even though the authors have explained them in the discussion section.
14. A schematic figure or graphical abstract would be helpful to understand the study

**Reviewer #2:**

Pirri et al study the role of endothelial EPAS1 expression in obesity and atherosclerosis using several mouse models, in vitro systems and human data. EPAS expression is triggered by low oscillatory shear stress, and limited by obesity and PHD3 expression in mice and humans. EC-specific Ko of EPAS1 aggravates atherogenesis, and its loss is associated with reduced fatty acid oxidation. The authors conclude that EPAS1 must be essential for FFA metabolism and EC homeostasis in atherosclerosis. Yet the authors overlook several other cell processes that may be involved in both EPAS1 stabilization and its effect on atherosclerosis.

Major

Sample size throughout is on the low side for experiments with HCD and atherosclerosis acc. to guidelines a minimum of 10 animals is advised (https://doi.org/10.1161/ATV.0000000000000006)

Why are only 3 animals per groups presented in figure 6, while in figure 5 there are 6-8 animals? In figure S2, the absence of an effect of hyperglycaemia may actually be a power problem

Why did the authors only investigate PHD3, and not the PHD1 and 2 isoforms? PHD3 is indeed an important regulator of EPAS1/HIf2 but mostly induced by hypoxia.

Sulforaphane is an anti-oxidant via NRf2, and these have been found to stabilize HIF proteins. This seems also a likely mechanism rather than an effect mediated by TG loss. The authors show that FFA enhance EPAS1, but this does not exclude an effect mediated by oxidative stress by sulforaphane as FFA also trigger ROS. Can the authors provide evidence in-/excluding this mechanism measuring ROS, EPAS1 and PHD2 w/wo anti-oxidants, w/wo sulforphane and FFAs?

The authors show that lipid receptors are upregulated upon EPAS1 loss, yet functional proof of increased lipid uptake is needed to substantiate functional changes.

The authors conclude that EPAS1 must be essential for EC homeostasis in atherosclerosis, yet do not show functional parameters of the endothelium. In addition to FFA metabolism, EPAS1 could also affect vasa vasorum density or maturation, and hence plaque inflammation via the outside-in theory. At least HIF1 is involved in collagen metabolism. All these processes (fibrosis, inflammation, angiogenesis) also affect lesion development, therefore please characterize the plaque and adventitia in more detail. Hence their conclusion on FFA metabolism and Ec homeostasis is not supported by data.

To further strengthen the interesting and valuable human link: do EPAS/PHD3 also correlate with plasma TGs, FFAs or antioxidant status?

Minor;

Statistics: was data distribution tested to ensure that parametric tests used are suitable? Sample size is not always high enough to assume normality (i.e. fig 1)
Figure 1C/2E the chose image for HSS does not seems representative for the data as there is No staining visible, while ~15% of the nuclei are quantified to be positive.
Figure 2IL label under graph is LSS not LOSS?
The antibodies for PHD3 are known not to be very reliable in the HIG field, and PHD's are very homologous: can the authors provide a specificity using PHD3 silencing.

Dear Professor Freedman,

**RE: CIRCRES/2023/324054/R1: EPAS1 Attenuates Atherosclerosis Initiation at Disturbed Flow Sites through Endothelial Fatty Acid Uptake**

We would like to thank you and the reviewers for your particularly thorough and helpful review of this manuscript. We were delighted to note that you see this paper as potentially important and have requested a revised version.

We have addressed each of the comments from the reviewers by performing a substantial amount of new experiments and analyses. This has resulted in significant and extensive new data leading to **6 new figures** and an **additional 7 new data panels** in existing figures. This new work prompted by excellent reviews has greatly enhanced the robustness of our findings and provided deeper mechanistic insights for the pathway of EPAS1 modulation by obesity and for the effects of EPAS1 on endothelial physiology. Our manuscript is now much stronger and hopefully acceptable for publication in *Circulation Research.*

These new findings include:

1. Demonstrating that lipid metabolism is enhanced at the inner curvature of the aorta compared to the outer curvature, by detected lipid droplets in mice exposed to acute hypertriglycaemia (Fig. S12).
2. Showing that plasma EPAS1 is reduced in obese mice compared to controls, thereby bridging the preclinical and clinical data (Fig. S7B)
3. Demonstrating that obesity reduces EPAS1 and enhances PHD2 in murine microvascular endothelium, again serving to bridge the pre-clinical and clinical observations (Fig. S7A)
4. Showing that palmitic acid and oleic acid can both reduce EPAS1 expression by enhancing the expression of the negative regulator PHD2 (Fig. 5A). Consistently, we observed that PHD2 expression at sites of low oscillatory shear stress was increased in obese mice, thereby confirming the role of PHD2 in obesity-related endothelial change (Fig. 5B).
5. We also looked more closely at the mechanism of sulforaphane rescue of EPAS1 in FFA-treated endothelium and observed that it acts, in part, via an antioxidant mechanism (Fig. 5A; Fig. S6)
6. Additional metabolic analyses and immunoblotting to show that EPAS1 has relatively modest effects on mitochondrial respiration per se and glycolysis (Fig. S11 and Fig. S13). This emphasises the primary function of EPAS1 that we have identified in regulating fatty acid b-oxidation.
7. Analysis of additional plaque features (macrophages, collagen, elastin; Fig. S9)

We submit a marked version (red font) and unmarked versions.

Our detailed response is given below (reviewer comments in bold):

**REVIEWER COMMENTS TO AUTHORS:**

**Reviewer #1:**
**The investigators demonstrate that endothelial cell (EC) EPAS1 contributes to the prevention of atherosclerosis in the arterial regions exposed to low/oscillatory shear stress. Using both in vitro and in vivo models, Priri et al. showed that EPAS1 attenuates atherosclerosis initiation by maintaining EC proliferation via fatty acid uptake and lipid metabolism, whereas the reduction of EPAS1 in an PHD3-dependent manner leads to atherosclerosis in the hyperlipidemia milieu/obesity. While this study is novel and significant, there are several major and minor concerns prior to publication.**

**Major:**
**1. The previous literature has established EPAS1 activation in chronic hypoxia; however, please discuss the relationship between LOSS and hypoxia in endothelial cells.**
We have discussed the relationship between LOSS and hypoxia as follows:
*"Here we demonstrate that LOSS can induce enhanced EPAS1 expression in cultured arterial EC. Under these conditions, oxygen levels are physiological or exceed physiological levels and therefore we conclude that LOSS can induce EPAS1 independently from hypoxia in arterial EC. Interestingly, empirical measurement of oxygen tensions in instrumented dogs indicate that a disturbed flow regions in carotid arteries experience reduced oxygen levels[38]. Thus, enhanced EPAS1 expression at atheroprone disturbed flow regions may be regulated by LOSS in conjunction with sub-physiological oxygen levels". (page 26, paragraph 1)*

**2. The authors conclude that activation of fatty acid metabolism by EPAS1 in aortic inner curvature prevents atherosclerosis. Please explain how EC fatty acid metabolism contributes to endothelial cell repair.**
We have revised the manuscript to explain the link between EC fatty acid metabolism and endothelial repair as follows:

*"Fatty acid b oxidation makes only a minor contribution to ATP production in endothelial cells with glycolysis being the primary driver of ATP production in this context[45]. Nevertheless, there is growing appreciation of the importance of fatty acid b oxidation as a critical regulator of EC physiology and metabolic homeostasis[46 47]. Fatty acid b oxidation is required for redox homeostasis via regeneration of NADPH in quiescent endothelium [48]. It is also needed for dNTP production needed for DNA replication in proliferating endothelium[37]. Here we demonstrated using Epas1$^{EC\text{-}KO}$ mice that endothelial expression of Epas1 positively regulates the expression of CD36 and LIPG which co-ordinate FFA entry to the endothelium. Crucially, proliferation was rescued in EPAS1-silenced cells by supplementation with exogenous FFAs (Fig. S14) suggesting that EPAS1-dependent fatty acid handling is a positive regulator of EC repair under disturbed flow." (page 28, paragraph 2).*

**3. Please provide evidence to demonstrate differential EC metabolism between inner vs. outer curvature. This difference would strengthen the the hyperlipidemia responses.**

We have addressed this experimentally by exposing fasted mice to a bolus of triacylglycerols (oral gavage of olive oil) and measuring lipid droplets in endothelium at inner and outer curvatures. These experiments confirmed differential lipid metabolism at these sites and was presented in Fig. S12 and described as follows:

*"To assess whether localized enrichment of CD36 and LIPG at the LOSS region is associated with elevated lipid metabolism we exposed mice to acute hyperlipidaemia using oral gavage of olive oil. Plasma levels of TG were significantly increased at 90 min post-gavage and declined in some animals at 180 min post gavage (Fig. S12A). Notably, en face staining with a lipophilic dye (BODIPY493/503) revealed enhanced accumulation of lipid droplets at the LOSS region of the aortic arch compared to the HSS region at 180 min (Fig. S12B), suggesting localised enhancement of lipid uptake". Page 24, paragraph 1.*

**4. The authors show that oscillatory hemodynamics is necessary to regulate EPAS1 in vitro and in vivo. However, microvascular ECs in adipose tissue are not exposed to oscillatory hemodynamics. Please explain this difference.**

This is a good point. We have revised the manuscript to provide the following explanation:

"EPAS1 expression was enriched at LOSS regions of the aorta and was also detected in microvascular EC which experience unidirectional laminar flow. Since microvascular EC experience intermittent blood flow it is plausible that EPAS1 expression in the microvasculature is regulated by oxygen tension, however this needs to be tested experimentally. Since microvascular EPAS1 is reduced in obesity, it will be interesting in future studies to discern whether this is linked to increased prevalence of hypertension in obese individuals". Page 27, paragraph 2.

**5. The authors measured plasma EPAS1 expression levels in human subjects. However, it is unclear whether plasma EPAS1 levels can be used as a surrogate for EPAS1that is released from ECs?**

To address this point we have performed additional analyses to demonstrate that EPAS1 in microvasculature and in serum is reduced in a murine model of obesity (Fig. S7), thus bridging our preclinical and clinical findings. We propose that microvascular endothelium contributes to the pool of serum EPAS1 and that reduction of serum EPAS1 in obesity may reflect endothelial change.

We have modified the revised manuscript as follows:

***"Microvascular EPAS1 and circulating serum EPAS1 are reduced in obese mice.*** *We assessed whether EPAS1 suppression in obesity could be observed in blood serum and peripheral microvasculature. Immunoflurescence staining revealed that EPAS1 was expressed in dermal microvasculature of non-obese mice (Fig. S7A) and ELISA revealed the presence of EPAS1 in blood serum of non-obese mice (Fig. S7B). Notably, both microvascular EPAS1 (Fig. S7A) and serum EPAS1 (Fig. S7B) were reduced in obesity induced by HFD. PHD2 exhibited the opposite pattern of expression since it was enhanced in microvascular EC of obese mice compared to*

*controls (Fig. S7A). Thus obesity in mice led to reduced EPAS1 in microvascular endothelium coupled to reduced serum EPAS1".* Page 19, paragraph 2.

**6. If EPAS1 affects endothelial cells in the microvasculature and resistance arteries, it would be interesting to show the blood pressure in human subjects. This is an interesting point. We revised the manuscript to include information on hypertension as follows:**

RESULTS

*"The obese cohort was associated with increased rates of hypertension (33.3 %) and diabetes/pre-diabetes (26.7 %) compared to previously analysed age-matched non-obese populations[35] (Table S3)" page 20, paragraph 1.*

DISCUSSION

*"Since microvascular EPAS1 is reduced in obesity, it will be interesting in future studies to discern whether this is linked to increased prevalence of hypertension in obese individuals". Page 27, paragraph 2.*

**7. The link between hyperlipidemia and EPAS1 requires further explanation**

We performed a series of additional experiments to address the mechanism linking hyperlipidemia and EPAS1. These new studies showed that palmitic acid and oleic acid can both reduce EPAS1 expression by enhancing the expression of the negative regulator PHD2 (Fig. 5A). Consistently, we observed that PHD2 expression at sites of low oscillatory shear stress was increased in obese mice, thereby confirming the role of PHD2 in obesity-related endothelial change (Fig. 5B). We also looked more closely at the mechanism of sulforaphane rescue of EPAS1 in FFA-treated endothelium and observed that it acts, in part, via an antioxidant mechanism (Fig. 5A; Fig. S6)

These studies are described as follows:

*"The observation that EPAS1 rescue correlated with reduced plasma TG in SFN-treated obese mice led us to hypothesize that TGs may be involved in EPAS1 suppression in obesity. This was tested by exposing PAEC cultured under LOSS to FFAs which are produced from local hydrolysis of TG. Immunoblotting revealed that EPAS1 levels were significantly reduced by exposure to PA or OA (Fig. 5A; compare lanes 1 and 4 & lanes 1 and 7). To investigate the underlying mechanism we performed immunoblotting of PHD2 and PHD3 proteins that target EPAS1 for proteasomal degradation[20]. PHD2 levels were significantly increased by PA or OA (Fig. 5A), suggesting that FFAs may reduce EPAS1 via PHD2 induction. The observation was validated by en face staining of aortas which demonstrated that PHD2 expression at the LOSS region was enhanced in obese mice fed a HFD compared to non-obese mice (Fig. 5B). Immunoblotting for PHD3 was inconclusive since commercial antibodies did not generate a specific signal (data not shown). Sulforaphane can exert direct effects on EC via activation of NRF2 and suppression of reactive oxygen species (ROS; [33 34]). The potential involvement of this pathway in EPAS1 rescue was investigated by treating PAEC with PA or OA in the presence or absence of sulforaphane or the antioxidant NAC. To validate the effects of these compounds on PAECs, it was demonstrated that sulforaphane and NAC can suppress ROS (Fig. S6) whereas SFN alone had the capacity to enhance NRF2*

*(Fig. 5A). Sulforaphane and NAC increased EPAS1 levels in PAEC co-treated with PA or OA (Fig 5A, compare lanes 4, 5, 6 & lanes 7, 8, 9), suggesting that sulforaphane can rescue FFA-mediated EPAS1 suppression by reducing ROS. Moreover, the mechanism may involve PHD2 which was reduced by SFN and NAC albeit not reaching statistical significance (Fig 5A). Thus EPAS1 rescue in sulforaphane-treated obese mice may involve direct effects of SFN on endothelium. Collectively, these observations suggest that EPAS1 reduction in obesity may result from hypertriglyceridemia-FFA metabolism coupled to localized alterations in cellular oxidative stress leading to the induction of endothelial PHD2". (page 18, paragraph 2).*

**8. The study should explore the different mechanisms in HSS and LOSS regions that both exhibit atherosclerosis protective phenotypes.**
We have revised the manuscript to compare the protective mechanisms that exist at HSS and LOSS regions as follows:

*"Arterial regions of uniform flow are characterized by physiological HSS which activates multiple protective proteins including eNOS, transcription regulators KLF2, NRF2 KLK10 [39 40]. Collectively, these molecules co-ordinate the upregulation of a suite of anti-apoptotic and anti-inflammatory molecules to reduce vascular injury and inflammation at high shear stress sites. In contrast, regions of disturbed flow are characterised by LOSS-dependent activation of multiple proatherogenic pathways[41], including BMP4-HOXB9[42], GATA4-TWIST1-SNAIL[43], and NOTCH4-JAG1[27] which collectively increase inflammation and impede vascular repair. Surprisingly, our murine model demonstrates that endothelial EPAS1 exhibits a protective effect against atherosclerosis at an atheroprone region exposed to LOSS. This is analogous to a previous observation that LOSS induces stress response genes which serve to maintain homeostasis at regions of disturbed flow [44]. Thus, EPAS1 and the stress response pathway are examples of homeostatic mechanisms that reduce the atherogenic process at predilection sites for atherosclerosis". (page 27, paragraph 3).*

**9. Streptozotocin (STZ) is also known to cause eNOS uncoupling and ROS production, leading to endothelial cell (EC) dysfunction. The specific effects of STZ on EC dysfunction and atherosclerosis need to be articulated.**
We have revised our manuscript to capture this as follows:

*"The potential contribution of hyperglycaemia was analysed by treating mice with STZ which induces diabetes associated with endothelial dysfunction and uncoupling of eNOS[31]". Page 17, paragraph 2*

*"Our subsequent experiments delineated the mechanism for EPAS1 regulation in obesity focussing on the potential effects of hyperlipidaemic and hyperglycaemic metabolic disturbances. Although STZ can cause EC dysfunction[31], we did not observe a reduced expression of EPAS1 in STV-treated mice that subsequently developed hyperglycaemia. This observation suggested that suppression of EPAS1 in obesity was not due to hyperglycaemia". Page 26, paragraph 2*

**10. The scRNAseq results include only a limited number of ECs, and these cells are mixed between groups. Please provide statistical significance with a small sample size.**
We have revised the manuscript to provide statistical analyses of the scRNAseq data which is presented in Fig S10C and Fig S10F and described as follows:

*"*Our sort-seq analysis identified 6 distinct clusters (Fig. S10B). Of these, clusters 0-4 were significantly enriched for multiple EC markers whereas cluster 5 was significantly enriched for VSMC-like features and was subsequently removed from analysis of EC heterogeneity (Fig. S10C). Considering the influence of *Epas1* on EC heterogeneity, the majority of cells in cluster 0 were from control mice, whereas clusters 1 and 4 contained cells mainly from *Epas1*$^{EC\text{-}KO}$ mice (Fig. S10D and Fig. S10E), indicating that *Epas1* influences EC heterogeneity. Functional annotation revealed that *Epas1*-regulated clusters were significantly associated with several GO terms associated with metabolic processes. Notably, the GO terms long-chain fatty acid transport, cGMP-mediated signalling – Lipoprotein and response to hypoxia were significantly enriched in cluster 4 (Fig. S10F). These data suggest that *Epas1* may be involved in fatty acid/lipoprotein metabolism in arterial endothelium.*" Page 22, paragraph 1*

**11. Oscillatory shear stress promotes glycolysis in ECs via PFKFB3. In contrast, the authors propose that in LOSS conditions, EPAS1 stimulates fatty acid metabolism, leading to EC proliferation and, subsequently, protection against atherosclerosis. The authors may need address how EC glycolysis is affected under LOSS and explain the protective role of proliferative ECs in this context.**
This is an excellent suggestion. We addressed it experimentally by interrogating the potential influence of EPAS1 on glycolysis. It was observed that EPAS1 silencing had a relatively modest effect (15% reduction) on glycolysis and had no effect on the expression on PFKFB3 or HK2 glycolysis enzymes. We present these new data in Fig S13 and describe them as follows:

*"Since HIF1A regulates glycolysis at regions of LOSS[9], we assessed the potential contribution of EPAS1 to this pathway using a Glycostress assay. In this assay, PAEC exposed to LOSS were first starved for glucose to establish a basal ECAR in a nutrient deprived setting followed by the injection of glucose, oligomycin and 2-DG. Silencing of EPAS1 reduced both glycolysis (Fig. S13A) and glycolytic capacity (Fig. S13B; a read out of metabolic plasticity) by approximately 15%. We therefore investigated the levels of HK2 or PFKFB3 which are key glycolytic modulators that are regulated by HIF1A[9]. Immunoblotting revealed that silencing of EPAS1 did not influence the expression of HK2 or PFKFB3 (Fig. S13C). Thus EPAS1 has relatively modest effects on glycolysis through a mechanism that is distinct to HIF1A".* page 24, paragraph 2

We also discuss the interplay between HIF1A, EPAS1 and proliferation in the context of atherosclerosis as follows:

*"Disturbed flow induces multiple injurious stimuli in arterial endothelium including heightened apoptosis and EndMT[49], and therefore we hypothesize that EPAS1-dependent EC proliferation is required for endothelial repair at these regions. This concept is consistent with our observation that endothelial Epas1 is atheroprotective. However, the relationship between endothelial cell turnover and atherosclerosis is intricate, as proliferation is essential for vascular repair but excessive proliferation may contribute to increased permeability to proatherogenic lipoproteins and endothelial activation. Intriguingly, this balance between the protective and proatherogenic effects of EC proliferation may involve a balance between EPAS1 and HIF1A. Previous research found that endothelial HIF1A promotes atherosclerosis[50] by driving inflammation and proliferation at atheroprone regions via upregulation of glycolysis[9 10 11]. By contrast, we observe that EPAS1 driven proliferation via fatty acid metabolism is coupled to atheroprotection. Given our observation of heterogenous expression of EPAS1 and HIF1A in atheroprone endothelium, we hypothesize that EPAS1+ HIF1A+ cells are "supercharged" for excessive proliferation because they are primed for HIF1A-glycolytic energy production and EPAS1-fatty acid b oxidation-driven dNTP production, leading to a proatherogenic phenotype. By contrast, we hypothesize that EPAS1+ HIF1A- cells are programmed for homeostatic proliferation that slows the initiation of atherosclerosis. However, further experimentation is needed to explore these concepts. Identifying mechanisms that shift the balance of HIF transcription factors to suppress HIF1 and enhance EPAS1 may offer therapeutic opportunities to promote vascular repair and reduce atherosclerosis." Page 29, paragraph 2.*

**11. Please elaborate as to why proliferative ECs have protective effects.**
We have addressed this as part of our revision for point 10 as follows:

*"Disturbed flow induces multiple injurious stimuli in arterial endothelium including heightened apoptosis and EndMT[49], and therefore we hypothesize that EPAS1-dependent EC proliferation is required for endothelial repair at these regions. This concept is consistent with our observation that endothelial Epas1 is atheroprotective. However, the relationship between endothelial cell turnover and atherosclerosis is intricate, as proliferation is essential for vascular repair but excessive proliferation may contribute to increased permeability to proatherogenic lipoproteins and endothelial activation". Page 29, paragraph 2.*

**12. The authors used oleic acid as a free fatty acid model for hyperlipidemia. Palmitic acid may better represent hyperlipidemic conditions.**
We agree and have now extended our analysis to include the influence of palmitic acid on endothelial function (Fig. 5).

**Minor:**
**1. In the abstract, where it mentions "Obese mice... correlating...", how did the authors determine this correlation?**
We have modified this sentence for clarity as follows:

*“Obese mice exhibited substantial reduction in endothelial EPAS1 expression. Sulforaphane, a compound with known atheroprotective effects, restored EPAS1 expression and concurrently reduced plasma triglyceride levels in obese mice.” Page 2, paragraph 3*

**2. In the introduction, is there evidence to show that HIF-2A does not regulate angiogenesis, unlike HIF-1A?**
EPAS1 plays an important role in angiogenesis and the manuscript has been revised to reflect this:

*“The function of EPAS1 in vascular biology is less explored. However, EPAS1 is known to promote vascularisation by stabilizing newly formed microvascular networks and by driving arteriogenesis[12 13 14 15].” Page 4, paragraph 2*

**3. Please provide more details on how blood pressure was measured using the CODA system.**
We have revised the methods section to provide these details, beginning page 6, paragraph 3.

**4. In the Seahorse experiment, the authors seemed to measure beta-oxidation to OCR levels using Etomoxir and BPETS+UK5099. However, for a comprehensive assessment of the respiratory phenotype in EPAS1 shRNA ECs, establishing a standard OCR curve initially, perhaps with agents such as Oligomycin, FCCP, Rotenone/Antimycin A, would be beneficial. Also, it would be advisable to modify the x-axis in Figure S8C to reflect time instead of measurement.**
We agree and have performed a series of additional experiments to establish a standard OCR curve using oligomycin, FCCP, Rotenone/Antimycin A (Fig. S11E and Fig. S11F). These data are described as follows:

“To further investigate the role of EPAS1 in respiration, we performed a Mitostress assay. This assay revealed that EPAS1 shRNA silencing had a modest but significant impact in reducing maximal respiratory rate (Fig. S11E) and ATP production (Fig. S11F). Thus, EPAS1 regulates respiration by controlling fatty acid β-oxidation and has relatively modest effects on mitochondrial activity *per se”. page 23, paragraph 1*

**5. There appears to be no expression of EPAS1 in the outer curvature (HSS). It would be appropriate to replace the representative images with those that align with the bar graph and findings in Figure 1A.**
Thank you. We have replaced this image with a more representative one.

**6. The effects of HFD on body weight, glycaemia, and TG are well-known. Figure 2A-C would be better placed in the supplementary.**
We agree. These data have been moved to the Supplementary Material section.

**7. The expression level of EPAS1 in Figure 2E is significantly higher than in Figure 1C, even for the chow diet group. Could this difference be attributed to age or body weight variations?**
Mean fluorescence intensity is an arbitrary unit depending on the parameters of excitation and detection. Figure 1C and Figure 2A (was Fig. 2E) were generated on different microscopes using different settings. So although each we can compare MFIs between experimental conditions within a particular experiment, we cannot meaningfully compare MFIs between different setups.

**8. Figures 2G-H and 3A-B would be better suited for the supplementary.**
These data have been moved to the Supplement.

**9. There seems to be some confusion regarding the definition of LOSS in the discussion; it was referred as laminar oscillatory shear stress.**
This has been corrected to low oscillatory shear stress.

**10. For better readership, consistent use of nomenclature throughout the paper.**
This has been corrected throughout.

**11. The immunofluorescence staining in Figure S4A is unclear. Please provide Western Blot (WB) data.**
We have performed Western blotting to show robustly that free fatty acids (oleic acid and palmitic acid) consistently reduce the expression of EPAS1 in cultured endothelium (Fig 5).

**12. Providing a table detailing the characteristics of human subjects would be necessary.**
This has been included as Table S3.

**13. Please discuss more LIPG, CD36, and PHD3 when the authors first introduce them on page 18, even though the authors have explained them in the discussion section.**
The manuscript has been revised to provide an introduction at first mention:

*"This was tested by quantifying levels of two molecules that co-ordinate endothelial handling of FFAs; LIPG which hydrolyses TGs into FFAs at the endothelial surface and CD36 which transport extracellular FFAs into EC[36]." Page 23, paragraph 2*

*"Under normal oxygen conditions, these proteins undergo prolyl hydroxylation co-ordinated by PHD proteins which triggers ubiquitination and proteasomal degradation[6]".* Page 4, paragraph 2

*“To investigate the underlying mechanism we performed immunoblotting of PHD2 and PHD3 proteins that target EPAS1 for proteasomal degradation[20].” Page 18, paragraph 2*

**14. A schematic figure or graphical abstract would be helpful to understand the study.**
This has been included in the revised version in the form of a graphical abstract.

**Reviewer #2:**
**Pirri et al study the role of endothelial EPAS1 expression in obesity and atherosclerosis using several mouse models, in vitro systems and human data. EPAS expression is triggered by low oscillatory shear stress, and limited by obesity and PHD3 expression in mice and humans. EC-specific Ko of EPAS1 aggravates atherogenesis, and its loss is associated with reduced fatty acid oxidation. The authors conclude that EPAS1 must be essential for FFA metabolism and EC homeostasis in atherosclerosis. Yet the authors overlook several other cell processes that may be involved in both EPAS1 stabilization and its effect on atherosclerosis.**

**Major**
**Sample size throughout is on the low side for experiments with HCD and atherosclerosis acc. to guidelines a minimum of 10 animals is advised (https://doi.org/10.1161/ATV.0000000000000006)**
This additional work would have required >9 months to re-establish breeders, generate experimental mice and perform the study. The editors agreed that this can be waived in order to retain publication priority.

**Why are only 3 animals per groups presented in figure 6, while in figure 5 there are 6-8 animals?**
We have generated additional conditional knockout mice and analysed CD36 and LIPG expression so that we consistently present 6 mice per group in the revised manuscript (Fig. 8D, Fig. 8E).

**In figure S2, the absence of an effect of hyperglycaemia may actually be a power problem.**
Thank you. We have reinterpreted these data which now appear in Fig. S4 and present them as follows:

“To corroborate this finding, we investigated the levels of EPAS1 in the aorta of $Lep^{ob/ob}$ mice during their hyperglycaemic phase at 14 weeks. Hyperglycaemic $Lep^{ob/ob}$ mice exhibited a relatively modest reduction in EPAS1 that did not reach statistical significance (Fig. S4), confirming that EPAS1 suppression in obesity can be uncoupled from hyperglycaemia.” Page 17, paragraph 2

**Why did the authors only investigate PHD3, and not the PHD1 and 2 isoforms? PHD3 is indeed an important regulator of EPAS1/HIf2 but mostly induced by hypoxia.**
This is an excellent suggestion. EPAS1 has been shown to be regulated by PHD2 and PHD3. We performed additional experiments to assess the effects of FFAs on PhD2 and PHD3 in cultured EC exposed to LOSS. They showed that FFAs induce PHD2, suggesting that PHD2 may be involved in EPAS1 suppression in hyperlipidemia (Fig. 5A). Consistent with these in vitro data, PHD2 expression in the mouse aortic arch was enhanced in obese mice exposed to a high fat diet compared to controls (Fig. 5B).

These new data were described as follows:
*"This was tested by exposing PAEC cultured under LOSS to FFAs which are produced from local hydrolysis of TG. Immunoblotting revealed that EPAS1 levels were significantly reduced by exposure to PA or OA (Fig. 5A; compare lanes 1 and 4 & lanes 1 and 7). To investigate the underlying mechanism we performed immunoblotting of PHD2 and PHD3 proteins that target EPAS1 for proteasomal degradation[20]. PHD2 levels were significantly increased by PA or OA (Fig. 5A), suggesting that FFAs may reduce EPAS1 via PHD2 induction. The observation was validated by en face staining of aortas which demonstrated that PHD2 expression at the LOSS region was enhanced in obese mice fed a HFD compared to non-obese mice (Fig. 5B). Immunoblotting for PHD3 was inconclusive since commercial antibodies did not generate a specific signal (data not shown)."* Page 18, paragraph 2

FFAs also induced a band that was recognised by anti-PHD3 antibodies, however this band was not reduced by PHD3 siRNA suggesting that it may be due to non-specific binding (potentially with PHD2). We have therefore revised the manuscript to focus on PHD2. Please see below 'Minor Comments" for further discussion of these blots.

**Sulforaphane is an anti-oxidant via NRf2, and these have been found to stabilize HIF proteins. This seems also a likely mechanism rather than an effect mediated by TG loss. The authors show that FFA enhance EPAS1, but this does not exclude an effect mediated by oxidative stress by sulforaphane as FFA also trigger ROS. Can the authors provide evidence in-/excluding this mechanism measuring ROS, EPAS1 and PHD2 w/wo anti-oxidants, w/wo sulforphane and FFAs?**
We thank the reviewer for this suggestion. We addressed this experimentally by treating endothelial cells under LOSS with palmitic acid or oleic acid in the presence/absence of SFN or NAC. As controls, we showed that SFN and NAC reduced ROS (Fig. S6), and that SFN activates NRF2 (Fig. 5A). Interestingly, SFN and NAC were able to partially rescue EPAS1 expression in cells exposed to FFAs, suggesting that SFN may have direct effects on EPAS1.

*This is described in the revised manuscript as follows:*
*"The potential involvement of this pathway in EPAS1 rescue was investigated by treating PAEC with PA or OA in the presence or absence of sulforaphane or the*

*antioxidant NAC. To validate the effects of these compounds on PAECs, it was demonstrated that sulforaphane and NAC can suppress ROS (Fig. S6) whereas SFN alone had the capacity to enhance NRF2 (Fig. 5A). Sulforaphane and NAC increased EPAS1 levels in PAEC co-treated with PA or OA (Fig 5A, compare lanes 4, 5, 6 & lanes 7, 8, 9), suggesting that sulforaphane can rescue FFA-mediated EPAS1 suppression by reducing ROS. Moreover, the mechanism may involve PHD2 which was reduced by SFN and NAC albeit not reaching statistical significance (Fig 5A). Thus EPAS1 rescue in sulforaphane-treated obese mice may involve direct effects of SFN on endothelium. Collectively, these observations suggest that EPAS1 reduction in obesity may result from hypertriglyceridemia-FFA metabolism coupled to localized alterations in cellular oxidative stress leading to the induction of endothelial PHD2." Beginning page 18, paragraph 2.*

**The authors show that lipid receptors are upregulated upon EPAS1 loss, yet functional proof of increased lipid uptake is needed to substantiate functional changes.**
We have addressed this by demonstrating that lipid droplets in endothelial cells are generated at higher levels at the LOSS region of the aortic arch (in mice exposed to a bolus of triacylglycerol-rich olive oil). This observation is consistent with enrichment of CD36 and LIPG at sites of LOSS. These data are presented in Fig. S12 and described in the revised manuscript as follows:

*"To assess whether localized enrichment of CD36 and LIPG at the LOSS region is associated with elevated lipid metabolism we exposed mice to acute hyperlipidaemia using oral gavage of olive oil. Plasma levels of TG were significantly increased at 90 min post-gavage and declined in some animals at 180 min post gavage (Fig. S12A). Notably, en face staining with a lipophilic dye (BODIPY493/503) revealed enhanced accumulation of lipid droplets at the LOSS region of the aortic arch compared to the HSS region at 180 min (Fig. S12B), suggesting localised enhancement of lipid uptake".* Page 24, paragraph 1

**The authors conclude that EPAS1 must be essential for EC homeostasis in atherosclerosis, yet do not show functional parameters of the endothelium. In addition to FFA metabolism, EPAS1 could also affect vasa vasorum density or maturation, and hence plaque inflammation via the outside-in theory. At least HIF1 is involved in collagen metabolism. All these processes (fibrosis, inflammation, angiogenesis) also affect lesion development, therefore please characterize the plaque and adventitia in more detail. Hence their conclusion on FFA metabolism and Ec homeostasis is not supported by data.**

We addressed this comment in Epas1$^{EC\text{-}KO}$ versus control mice by analysing the following features of plaque instability: macrophage content (CD68 staining), collagen content (Picrosirius Red), elastin breaks and presence of necrotic material. These data are presented in Fig S9 and described as follows:

*"The potential influence of Epas1 on plaque composition was next assessed. Endothelial deletion did not influence macrophage accumulation (Fig. S9A), elastin breaks (Fig. S9B), intraplaque necrosis (Fig. S9C). Collagen was not detected in*

*plaques of either group (Fig. S9D). These data show that endothelial EPAS1 protects against atherosclerosis but had negligible effects on plaque composition in this model". Page 21, paragraph 2*

It should be noted that the model that we used (AAV-PCSK9/HFD for 8 weeks) provides a representation of early atherosclerosis and that features of plaque instability such as necrotic core and adventitial angiogenesis are rare. We point this out as a limitation in the revised manuscript as follows:

"*Epas1* deletion did not influence plaque composition in the AAV-PCSK9 model. However, it should be noted that this model mimics features of early atherosclerosis and that further research using other models (e.g. *ApoE-/-*) is required to fully appreciate the role of *Epas1* in disease progression." *Page 28, paragraph 2.*

**To further strengthen the interesting and valuable human link: do EPAS/PHD3 also correlate with plasma TGs, FFAs or antioxidant status?**
This is an interesting point. In the revised manuscript we include clinical data including levels of plasma TGs (Table S3) which were raised compared to non-obese controls. We also performed ELISA to measure plasma FFAs and ROS which were similar between groups (Fig. 6B and Fig. 6C). We presented and discussed these data as follows:

*"We investigated whether reduced serum EPAS1 in clinical obesity was associated with altered circulating lipids and ROS. Levels of FFAs were modestly increased in obese compared to non-obese individuals however this difference did not reach statistical significance (Fig. 6B). Total antioxidant capacity (TAC) of serum was similar between obese and non-obese groups (Fig. 6C)".* page 20, paragraph 2

**Minor;**
**Statistics: was data distribution tested to ensure that parametric tests used are suitable?**
**Sample size is not always high enough to assume normality (i.e. fig 1)**
We have analysed all data for normality and adjusted the tests used where necessary.

**Figure 1C/2E the chose image for HSS does not seems representative for the data as there is No staining visible, while ~15% of the nuclei are quantified to be positive.**
We have replaced with more representative images.

**Figure 2IL label under graph is LSS not LOSS?**
This has been corrected.

**The antibodies for PHD3 are known not to be very reliable in the HIG field, and PHD's are very homologous: can the authors provide a specificity using PHD3 silencing.**
We are very grateful to the reviewer for pointing this out. To address this concern we used siRNA for PHD3 to test the specificity of anti-PHD3 in Western blotting.

In several experiments, we found that treatment with siRNA targeting PHD3 did **not** reduce a 40 kD band generated using anti-PHD3 that we had assumed to be PHD3. This was the case even when silencing was applied for 72 h or 96 h – blot shown below for reference. We know that the silencing was effective by qPCR measurements. This suggested that anti-PHD3 antibodies were non-specific. We suspect that the anti-PHD3 was cross-reacting with PHD2 and have therefore focussed the manuscript on PHD2 using previously-validated antibodies.

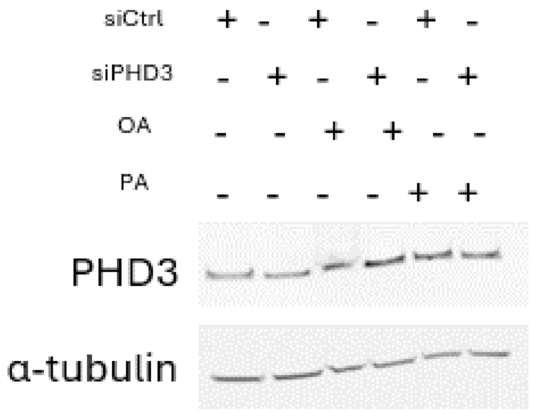

May 20, 2024

Prof. Paul C. Evans
Queen Mary University of London
William Harvey Research Institute
Charterhouse Square
London EC1M 6BQ
United Kingdom

RE: CIRCRES/2023/324054R1: EPAS1 Attenuates Atherosclerosis Initiation at Disturbed Flow Sites through Endothelial Fatty Acid Uptake

Dear Dr. Evans:

Your manuscript has been carefully evaluated by 4 external reviewers and the editors as an Original Research manuscript. We regret to inform you that the paper is not acceptable for publication in its present form.

As you will gather from the reviews, the referees identified a number of issues that must be clarified, particularly with regard to the statistical analyses. The editors concur.

Despite these concerns, the editors see this paper as potentially important and wish to encourage revision. If you would like to revise the manuscript in accordance with the suggestions of the reviewers and editors, we would be willing to evaluate a new version. The manuscript would be reviewed again, with no assurance of acceptance.

The Editors strongly encourage you to adhere to the journal's Statistical Reporting Recommendations in your revision, which can be found here: https://www.ahajournals.org/statistical-recommendations.

Upon revision, authors of manuscripts that contain cropped gels/blots will be required to submit a separate PDF file that contains the entire unedited gel for all representative cropped gels in the manuscript. Authors should label each gel as "Full unedited gel for Figure _" and highlight which lanes of the unedited gel correspond to those shown in the cropped images within the manuscript. For more information, please go to https://www.ahajournals.org/res/manuscript-preparation.

All research materials listed in the Methods should be included in the Major Resources Table file, which will be posted online as PDF with the article Supplemental Materials if the manuscript is accepted. A template Major Resources Table file (.docx) is available for download here: AHAJournals_MajorResourcesTable_2019.docx. Authors are required to upload the Table at the revision stage. Authors should reference the PDF in their Methods as follows: "Please see the Major Resources Table in the Supplemental Materials."

To read the comments to authors from the reviewers, please see below.

Please note that revised and resubmitted manuscripts are not assured of publication, and that fewer than 15% of all papers submitted to Circulation Research are eventually published.

Our current guidelines allow authors 90 days to complete the revision. If the manuscript is resubmitted within 90 days, one or more of the original reviewers will be re-consulted; the editors may also choose to obtain additional opinions from new reviewers. If you need more than 90 days to submit a revised paper, please notify the editorial office. In general, extensions over the revision time limit will not be granted except under special circumstances at the editors' discretion.

You are encouraged to log in and start the revision submission process as soon as possible, because all authors must complete disclosure and publication agreement forms BEFORE the revision can be submitted. As corresponding author, you may start the revision process at any time and go to the Copyright & Licensing tab to contact coauthor(s) and send reminders. In addition, please review all revision submission tabs to ensure that you are prepared to provide the appropriate files, checklists, and other items when it is time to submit your revision.

If you choose to revise, please include a detailed response to each of the referees' and editors' comments, providing each comment verbatim in bold followed by your response and giving the exact page number(s), paragraph(s), and line number(s) where each revision was made. If you make substantive changes to the manuscript, please provide a clear description of what you did and where. If you insert important sentences, paragraphs, or sections in response to the comments, please also include them in your response. Please indicate clearly any deletions. Additionally, a marked up version of the revision with the changes highlighted or tracked should be uploaded as a supplemental file. Number each page in the top right corner, using your manuscript number followed by /R2 to denote a second revision.

We are piloting an integration with SciScore (https://www.sciscore.com) to provide authors automatically generated reports during revision submission containing a reproducibility score and tables on rigor adherence and key resources such as antibodies, experimental models, recombinant DNA, and software. You are also welcome to start the revision submission process at any time to receive your report. We strongly encourage you to use the provided report while revising your manuscript to improve the study's reproducibility and reporting quality.

Please ascertain that your revised manuscript adheres to the Instructions to Authors as they appear online at https://www.ahajournals.org/res/author-instructions. Revisions that do not conform to the current limits on numbers of words (8000 total) may be returned to the authors for abbreviation. If you cannot reduce the overall word count, the editors may deem an extended print version appropriate; the authors should provide written assurance that they will cover the costs of the pages that are in excess of these limits. Note that paying for excess display items is not an option. Please refer to the Instructions to Authors for further details regarding our policy on page limits, articles with extended print versions, and related costs. No such limits apply to the online supplementary information, which can include supporting data and/or expanded text to offset the limits on the print version. Such online supplementary information can be cited in the print version as appropriate.

All corresponding authors of articles accepted to AHA Journals are required to link an ORCID iD to their profile in the AHA Journal submission system. To avoid potential processing delays in future, we recommend that you link an ORCID iD to your profile when you submit your revision. To register with ORCID or link your profile, please go to “Modify Profile/Password” on the submission site homepage, and click the link in the “ORCID” section.

We wish to thank you for having submitted this manuscript to Circulation Research.

Join us in Chicago this summer for #BCVS24! Register here: https://professional.heart.org/en/meetings/basic-cardiovascular-sciences

Sincerely,

Jane E. Freedman, MD
Editor-in-Chief
Circulation Research
An American Heart Association Journal

******************************************************************************************

**Reviewer comments to the Authors:**

**Reviewer #1:**

The authors have addressed all the reviewer's comments with an extensively revised manuscript and new experiments. Two major concerns and minor comments remain to strengthen this manuscript.
Major comments
1. The authors provided additional evidence to show that plasma EPAS1 levels in HFD vs. chow diet. However, to determine whether the plasma EPAS1 derived from ECs, the authors may consider plasma EPAS1 levels in Epas1EC-KO mice vs. WD with HFD.
2. In the scRNA-seq analysis result section, the authors described "cluster 1 and 4 contained cells mainly from Epas1EC-KO mice…". However, it is hard to discern whether these clusters primarily consist of ECs from Epas1EC-KO mice. Also, there was no clear separation between groups. To clarify, the authors may analyze the GO terms between WT vs. KO mice in each subcluster. Also, providing other GO terms associated with the manuscript such as proliferation, glycolysis, DNA replication, etc., would strengthen the manuscript.
Minor comments
1. In the scRNAseq methods, please provide how many cells were analyzed following QC.
2. In Fig. S10 B and D, please annotate the UMAP1 and UMAP 2 axes. Also, please elucidate the criteria used for the representative GO terms. Are the representative GO terms from the top 10 or specific targeted GO terms?

**Reviewer #2:**
No further comments.

**Statistical Reviewer:**
1. If the statistical test assumes a normal distribution, please state the test of normality used. If the data are not normally distributed or if the sample size is too small to assess normality (<10), please use a non-parametric alternative. If relying on statistical properties for normality (e.g. the central limit theorem), explicitly state the property and what data it applies to. Please note data points across treatment conditions should not be pooled for testing normality.
2. Please provide precise p-values (rather than P<0.0x). This can be obtained in GraphPad by increasing significant digits on the "Options" tab. Scientific notation with 2 significant figures (e.g. 1.2x10^-3) is strongly encouraged.
3. For each presented p-value, explicitly state the corresponding test it is derived from. If it is adjusted, specify what (and how many tests) it is adjusted for.
4. Please provide the exact N per group rather than ranges.
5. How were representative images selected for inclusion?
6. Consider multiple testing correction. The more tests done the higher the chance of observing a false association. If you chose not to correct for multiple testing, state this as a weakness of the study.
7. Please specify the software used for statistical analysis.
8. You cannot make claims of no change or no effect. You can say no statistically significant difference was observed. Eg the title of figure 3.
If you need to make claims of similarity consider specialized testing such as the two one-sided tests (TOST) for equivalence
9. If multiple samples were taken from the same animal, please account for the violation of the assumption of independent sampling in statistical modeling.
https://www.ncbi.nlm.nih.gov/pmc/articles/PMC5852514/pdf/cvx151.pdf
https://www.nature.com/articles/nmeth.3137
conditional hierarchical linear mixed effect model used (lme4 R package)
10. Please note that RNA-seq analyses with very small sample sizes can have inflated false positive rates, and sample size in such analyses should be justified via power analyses. Baccarella, A., Williams, C.R., Parrish, J.Z. et al. Empirical assessment of the impact of sample number and read depth on RNA-Seq analysis workflow performance. BMC Bioinformatics 19, 423 (2018). https://doi.org/10.1186/s12859-018-2445-2 Given the small sample size of your

RNA-seq experiments, please avoid drawing unjustifiably strong conclusions and making claims about statistical significance.
11. P-values should not be represented as bar-charts. This information would be more clearly conveyed in a table. Rather than using bar charts to represent results from pathway enrichment analyses, consider whether dot plots that also illustrate gene ratios might be more informative. https://guangchuangyu.github.io/2015/06/dotplot-for-enrichment-result/
12. A one-way ANOVA is not appropriate in figure 5a where you are looking across treatment conditions and presence of OA/PA.

**Technical Reviewer:**
The current study was carefully evaluated for inclusion of guideline items present in the Circulation Research checklists for rigor, transparency, and reproducibility. The reviewer has identified a number of items that were either omitted or not adequately addressed in the text. Please see below for details:

In vitro checklist items:
• Please clarify whether data points are technical or biological. For example, in the legend for Figure 1 the authors have nicely stated that each data point is an animal. Please clarify for the other figures as appropriate.
• Additional details regarding cell culture experiments should be provided, including passage number, number of cells, etc)
• Please provide uncropped immunoblots for technical review including the original ladder and indicate which lanes were used for the creation of the final figure. Please provide all blots that were used for quantification in both the main and supplemental figures, not just the representative images.
• Molecular weight sizes are missing from Figure S13C.
• Scale bars should be included on all micrographs and images in both the main and supplemental figures.
• Where immunostaining procedures are detailed, briefly describe the controls used to validate antibody specificity and distinguish genuine target staining from background (i.e. isotype vs secondary antibody only controls)
• In the atherosclerotic plaque analysis, how many sections per mouse were analyzed and at what distance from each other?

In vivo checklist items:
• Since the authors are using multiple sets of animals that are on both C57BL6/J and C57BL6/N backgrounds, please clarify the background of the Lep ob/ob and Epas flox/flox mice. I assume these are N and J respectively, but please state clearly which were used as controls for which genetically modified animal.
• Were only male mice used for the study? If so, please provide a statement of rationale for the exclusion of females.

Other:
• How were representative images/figures chosen? Are they representative of the mean/average or the best/illustrative images? Please note this in the text somewhere.
• Per the journal's requirements, please complete and submit a "Major Resources Table". Some of this information is included in the supplemental figures but should be converted to the Major Resources Table. Please refer to the website for formatting instructions.
• Where will the authors make their sequencing data public? Please provide database name and accession number.
• On my copy of the figures, there is a weird glitch in Supplemental Figure S2C where a bar is missing from the figure.

Dear Professor Freedman,

**RE: CIRCRES/2023/324054/R2: EPAS1 Attenuates Atherosclerosis Initiation at Disturbed Flow Sites through Endothelial Fatty Acid Uptake**

We would like to thank you and the reviewers for your particularly thorough and helpful review of this manuscript. We were delighted to note that you see this paper as potentially important and that we have been encouraged us to submit a revised version.

We have addressed the two remaining major comments and two minor comments from reviewer 1 by performing an additional experiment and additional data analysis. This has resulted in new data and analyses leading to an **additional 4 new data panels**. We have significantly improved the presentation and description of the scRNAseq data and thank the reviewer for prompting this. We have addressed each of the comments from the Statistical Reviewer and the Technical Reviewer and the manuscript is now more robust.

Our manuscript is now much stronger and hopefully acceptable for publication in *Circulation Research.*

We submit a marked version (red font) and unmarked versions.

Our detailed response is given below:

**Reviewer #1:**

**The authors have addressed all the reviewer's comments with an extensively revised manuscript and new experiments. Two major concerns and minor comments remain to strengthen this manuscript.**

**Major comments**

**1. The authors provided additional evidence to show that plasma EPAS1 levels in HFD vs. chow diet. However, to determine whether the plasma EPAS1 derived from ECs, the authors may consider plasma EPAS1 levels in Epas1EC-KO mice vs. WD with HFD.**

This is an excellent suggestion. We addressed it experimentally by analysing the levels of EPAS1 in plasma samples from *Epas1* EC-KO (N=12) vs. *Epas1* WT (N=10) mice exposed to 8 weeks HFD. *Epas1* deletion in endothelium lead to a reduction in circulating EPAS1 indicating that endothelium contributes to plasma levels of EPAS1. These data are presented in the revised manuscript in Fig. S7C and described as follows:

"To analyse whether endothelium contributes to circulating EPAS1, we deleted endothelial *Epas1* by crossing *Cdh5*$^{CreERT2/+}$ and *Epas1*$^{fl/fl}$ mice to generate *Epas1*$^{EC-KO}$ and control *Epas1*$^{EC-WT}$ mice. Tamoxifen treatment of *Epas1*$^{EC-KO}$ and control *Epas1*$^{EC-WT}$ mice induced deletion of *Epas1* which was validated by qRT-PCR of endothelial RNA (Fig. S8A) and by *en face* staining (Fig. S8B). *Epas1*$^{EC-KO}$ and *Epas1*$^{EC-WT}$ mice were exposed to a HFD for 8 weeks. ELISA revealed that serum

EPAS1 levels were significantly reduced in *Epas1*$^{EC-KO}$ compared to *Epas1*$^{EC-WT}$ mice (Fig. S7C), indicating that endothelium contributes to the pool of circulating EPAS1." Page 20, paragraph 2

**2. In the scRNA-seq analysis result section, the authors described "cluster 1 and 4 contained cells mainly from Epas1EC-KO mice…". However, it is hard to discern whether these clusters primarily consist of ECs from Epas1EC-KO mice. Also, there was no clear separation between groups. To clarify, the authors may analyze the GO terms between WT vs. KO mice in each subcluster. Also, providing other GO terms associated with the manuscript such as proliferation, glycolysis, DNA replication, etc., would strengthen the manuscript.**

We agree with the reviewer that further clarity was required for the scRNAseq data and have therefore revised the manuscript as follows:

We have substituted the pie chart for a 'waffle chart' which shows more clearly the contribution of Epas1EC-KO vs WT mice to each cluster (Fig. S10E). We have also modified the narrative to state the proportion of cells from Epas1EC-KO and WT mice contributing to each cluster as follows:

"Considering the potential influence of *Epas1* on EC heterogeneity, cluster 0 was enriched with cells from *Epas1*$^{EC-WT}$ mice (34% of the *Epas1*$^{EC-WT}$ cell population vs 19% of the *Epas1*$^{EC-KO}$ population) (Fig. S10D and Fig. S10E). By contrast, cluster 1 was enriched with cells from *Epas1*$^{EC-KO}$ mice (23% of the *Epas1*$^{EC-WT}$ cell population vs 34% of the *Epas1*$^{EC-KO}$ population), and cluster 4 was enriched with cells from *Epas1*$^{EC-KO}$ mice (3% of the *Epas1*$^{EC-WT}$ cell population vs 8% of the *Epas1*$^{EC-KO}$ population) (Fig. S10E)". page 23, paragraph 2.

As suggested by the reviewer, we have performed functional annotation of the clusters that were altered by *Epas1* endothelial deletion, including analysis of the GO terms enriched in Epas1EC-KO vs WT mice, and these data are presented in Fig. S11. In describing these data, we have also acknowledged the comments from the Statistical Reviewer that the scRNAseq was based on a relatively small number of cells and that we should "avoid drawing unjustifiably strong conclusions." Thus, we have described these data succinctly and highlighted their value in generating hypotheses for the function of endothelial *Epas1*, which we tested in subsequent experiments. This is described in the revised manuscript as follows:

"Functional annotation revealed that *Epas1*-regulated clusters were enriched for multiple diverse GO terms including intracellular signaling pathways, transcription/RNA processing, cell proliferation and metabolism (e.g. mitochondrial function, lipid transport) (Fig. S10F). Focusing on proliferation and metabolism, we observed that enrichment of the terms DNA replication', 'Positive regulator of endothelial proliferation' and 'Positive regulation glycolytic process' varied between clusters 0, 1 and 4 (Fig. S11A). Further functional annotation of cells from *Epas1*$^{EC-KO}$ vs *Epas1*$^{EC-WT}$ mice revealed multiple enriched GO terms including those linked to actin, Golgi apparatus and membrane (Cluster 0), translation, transport, oxidative phosphorylation, mitochondrion (Cluster 1) and development, MAP kinase signalling and glycoprotein (Cluster 4) (Fig. S11B). Overall, these data suggest lead us to

hypothesize that EPAS1 may be involved in metabolic regulation amongst other functions in arterial endothelium". page 23, paragraph 2.

**Minor comments**

**1. In the scRNAseq methods, please provide how many cells were analyzed following QC.**

To clarify this point, we have revised the Methods section as follows:

"454 cells were analysed after quality control; 262 cells (58%) from *Epas1*$^{EC\text{-}WT}$ mice and 192 cells (42%) from *Epas1*$^{EC\text{-}KO}$ mice." Page 10, paragraph 2.

**2. In Fig. S10 B and D, please annotate the UMAP1 and UMAP 2 axes.**

We have revised the manuscript to include axes for tSNE plots in Fig. S10B and D.

**Also, please elucidate the criteria used for the representative GO terms. Are the representative GO terms from the top 10 or specific targeted GO terms?**

We have modified the manuscript to clarify this point as follows:

"Differential expression analysis was performed between clusters. Genes were defined as enriched when their expression in a particular cluster was significantly different from the rest of the clusters using the Wilcoxon rank-sum test with p adjusted value < 0.001. These genes were selected for subsequent gene ontology (GO) analysis using the DAVID Functional Annotation Bioinformatics Analysis software." Page 11, paragraph 1.

**Reviewer #2:**

**No further comments.**

We thank the reviewer for their thorough review, which has significantly helped us enhance the quality of this manuscript.

**Statistical Reviewer:**

**1. If the statistical test assumes a normal distribution, please state the test of normality used. If the data are not normally distributed or if the sample size is too small to assess normality (<10), please use a non-parametric alternative. If relying on statistical properties for normality (e.g. the central limit theorem), explicitly state the property and what data it applies to. Please note data points across treatment conditions should not be pooled for testing normality.**

We have tested each dataset for normality and amended the statistical test used when appropriate. This is stated in the revised manuscript as follows:

"Data were analyzed for normality using the Shapiro-Wilk normality test. Data following a parametric distribution were analysed using ANOVA or t-tests. Non-parametric data were analysed using Kruskal-Wallis or Kologorov-Smirnov tests. GraphPad Prism software was used." page 14, paragraph 3.

**2. Please provide precise p-values (rather than P<0.0x). This can be obtained in GraphPad by increasing significant digits on the "Options" tab. Scientific notation with 2 significant figures (e.g. 1.2x10^-3) is strongly encouraged.**
Following this instruction, we have now revised our manuscript to show precise p-values in each of the data sets presented.

**3. For each presented p-value, explicitly state the corresponding test it is derived from. If it is adjusted, specify what (and how many tests) it is adjusted for.**
We have revised the figure legends of the manuscript to provide a description of the statistical test used in each experiment.

**4. Please provide the exact N per group rather than ranges.**
We have revised the figure legends of the manuscript to provide an exact N per group for each experiment.

**5. How were representative images selected for inclusion?**
Representative images of staining and western blots are presented throughout the manuscript and represent the average result obtained across multiple experiments. We have revised the manuscript to explain this as follows:
"Representative images were chosen to represent the average results achieved within the experiment". Page 14, paragraph 3.

**6. Consider multiple testing correction. The more tests done the higher the chance of observing a false association. If you chose not to correct for multiple testing, state this as a weakness of the study.**
Multiple comparison in ANOVA were corrected using the Sìdak multiple comparison test and this is now stated in the revised manuscript (page 15, paragraph 1).

**7. Please specify the software used for statistical analysis.**
All statistical analysis was performed using GraphPad Prism 10 as stated in the methods section (page 15, paragraph 1).

**8. You cannot make claims of no change or no effect. You can say no statistically significant difference was observed. Eg the title of figure 3. If you need to make claims of similarity consider specialized testing such as the two one-sided tests (TOST) for equivalence**
We have revised our manuscript and amended the title in Figure 3 by replacing with "Figure 3. Hyperglycaemia had no statistically significant effect on EPAS1".

**9. If multiple samples were taken from the same animal, please account for the violation of the assumption of independent sampling in statistical modeling. https://www.ncbi.nlm.nih.gov/pmc/articles/PMC5852514/pdf/cvx151.pdf**

https://www.nature.com/articles/nmeth.3137

**conditional hierarchical linear mixed effect model used (lme4 R package)**

Throughout our manuscript, we present data generated from testing of multiple animals. Within the graphs each data point represents an animal and the assumption of independent sampling was not violated.

**10. Please note that RNA-seq analyses with very small sample sizes can have inflated false positive rates, and sample size in such analyses should be justified via power analyses. Baccarella, A., Williams, C.R., Parrish, J.Z. et al. Empirical assessment of the impact of sample number and read depth on RNA-Seq analysis workflow performance. BMC Bioinformatics 19, 423 (2018). https://doi.org/10.1186/s12859-018-2445-2 Given the small sample size of your RNA-seq experiments, please avoid drawing unjustifiably strong conclusions and making claims about statistical significance.**

We agree with the reviewer and have revised the manuscript to avoid making strong conclusions. For example, instead of stating that the data "indicate" we now use the word "suggest". In addition, we present the scRNAseq data as a means to generate hypotheses that were subsequently validated with further focussed experimentation, as follows:

"To gain initial insight into the function of endothelial EPAS1 we performed single cell RNAseq from aortas of *Epas1$^{EC\text{-}KO}$* and control (*Epas1$^{EC\text{-}WT}$*) mice." Page 23, paragraph 1

"Overall, these data lead us to hypothesize that EPAS1 may be involved in metabolic regulation amongst other functions in arterial endothelium." Page 24, paragraph 1

**11. P-values should not be represented as bar-charts. This information would be more clearly conveyed in a table. Rather than using bar charts to represent results from pathway enrichment analyses, consider whether dot plots that also illustrate gene ratios might be more informative. https://guangchuangyu.github.io/2015/06/dotplot-for-enrichment-result/**

In the revised manuscript we present enrichment scores in Fig S10F and Fig. S11B. The bar charts represent enrichment scores with p-values for each GO term annotated on the right side. This may not have been sufficiantly clear in the original version and therefore we have modified the figure legends to provide clarity as follows:

Fig. S10F "Representation of enriched GO pathways for clusters 0, 1 and 4 and annotated p-value"

Fig. S11B "(B) Enrichment scores of enriched GO pathways for cells from *Epas1$^{EC\text{-}KO}$* or *Epas1$^{EC\text{-}WT}$* in clusters 0, 1 and 4 and annotated p-values"

**12. A one-way ANOVA is not appropriate in figure 5a where you are looking across treatment conditions and presence of OA/PA.**

We agree and have re-analysed the the data presented in Figure 5A using a two-way ANOVA. The analysis has been carried out using a Sidak multiple comparison test

and the figure and figure legend modified accordingly. This reanalysis did not alter the conclusions from this experiment.

**Technical Reviewer:**

**The current study was carefully evaluated for inclusion of guideline items present in the Circulation Research checklists for rigor, transparency, and reproducibility. The reviewer has identified a number of items that were either omitted or not adequately addressed in the text. Please see below for details:**

**In vitro checklist items:**

**• Please clarify whether data points are technical or biological. For example, in the legend for Figure 1 the authors have nicely stated that each data point is an animal. Please clarify for the other figures as appropriate.**

This has now been amended in our resubmitted version and all figure legends state whether technical or biological replicates are represented in each figure.

**• Additional details regarding cell culture experiments should be provided, including passage number, number of cells, etc)**

We have now added this information within the methods section.

**• Please provide uncropped immunoblots for technical review including the original ladder and indicate which lanes were used for the creation of the final figure. Please provide all blots that were used for quantification in both the main and supplemental figures, not just the representative images.**

Original blots have been submitted in a supplementary file named: "Full unedited gel for Figure_PirriD Circ Res 2024". All uncropped blots used for quantification have been attached within this file. Blots used within the manuscript are highlighted with a red frame.

**• Molecular weight sizes are missing from Figure S13C.**

This has now been amended in our updated manuscript.

**• Scale bars should be included on all micrographs and images in both the main and supplemental figures**

This has now been amended for all micrographs and images in our revised manuscript.

**• Where immunostaining procedures are detailed, briefly describe the controls used to validate antibody specificity and distinguish genuine target staining from background (i.e. isotype vs secondary antibody only controls)**

Antibodies used in immunofluorescent staining of mice tissue were validated using IgG isotype controls for target proteins. A list of antibodies is provided in the Table S2 including control IgG antibodies. We integrated this information in the text within the

Methods section by stating the use of appropriate IgG controls to validate antibody specificity.

**• In the atherosclerotic plaque analysis, how many sections per mouse were analyzed and at what distance from each other?**
Plaque sections were obtained at 7um distance from each other and all plaques analysis were performed on three sections per animal. Details for this analysis is now amended in the methods section (page 9, paragraph 1).

**In vivo checklist items:**
**• Since the authors are using multiple sets of animals that are on both C57BL6/J and C57BL6/N backgrounds, please clarify the background of the Lep ob/ob and Epas flox/flox mice. I assume these are N and J respectively, but please state clearly which were used as controls for which genetically modified animal.**
We apologise for not clarifying this point correctly in the methods section. Oral gavage experiments, Lep ob/ob, EPAS1 flox/flox and HFD mice on a 45% kcal western diet were on a C57BL6/J background, whereas mice on a HFD 60% kcal used in Figure 2 and affiliated supplementary figures were on a N background. This has now been clarified in the Methods section (pages 6 and 7) and in Figure legends. All animal experiments were performed with littermate controls (EPAS1 flox/flox and Lep ob/ob mice) or with age, gender and background match controls (HFD and olive oil oral gavage experiments) and this has been stated in the Figure legends of the revised manuscript.

**• Were only male mice used for the study? If so, please provide a statement of rationale for the exclusion of females.**
In this study, we made use of both female and male mice. No specified gender was excluded from the analysis.

**Other:**
**• How were representative images/figures chosen? Are they representative of the mean/average or the best/illustrative images? Please note this in the text somewhere.**
Representative images of staining and western blots are presented throughout the manuscript and represent the average result obtained across experiments. We have revised the manuscript to explain this as follows:

"Representative images were chosen to represent the average results achieved within the experiment". Page 14, paragraph 3.

**• Per the journal's requirements, please complete and submit a "Major Resources Table". Some of this information is included in the supplemental figures but should be converted to the Major Resources Table. Please refer to the website for formatting instructions.**
We have now completed a Major Resource Table and submitted it to accompany our revised manuscript.

**• Where will the authors make their sequencing data public? Please provide database name and accession number.**

We have prepared our scRNAseq data for submission to NCBI Gene Expression Omnibus including raw and processed data files and associated metadata. We will submit and release these data immediately upon acceptance of our manuscript.

**• On my copy of the figures, there is a weird glitch in Supplemental Figure S2C where a bar is missing from the figure.**

At our end, we did not find any issue with either the PDF and PPT version of this figure. We are happy to provide you with additional files (prism or excel) if this issue persists in the current re-submitted version. A copy of Fig S2C is shown here:

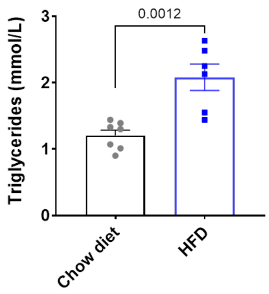

August 7, 2024

Dr. Paul C. Evans
Queen Mary University of London
William Harvey Research Institute
Charterhouse Square
London EC1M 6BQ
United Kingdom

RE: CIRCRES/2023/324054R2: EPAS1 Attenuates Atherosclerosis Initiation at Disturbed Flow Sites through Endothelial Fatty Acid Uptake

Dear Dr. Evans:

Your revised manuscript has been carefully evaluated by one external reviewer, a statistical reviewer, a technical reviewer, and the editors as an Original Research manuscript. While we are interested in your paper, further revision is required before we can accept the manuscript for publication in Circulation Research.

Please read the entire content of this letter and carefully address all of the comments in the reviewers' critiques and all of the formatting concerns described below. Please note that the paper cannot be accepted until you have addressed both the reviewers' critiques and all of the formatting issues. Please submit your revision by AUGUST 20.

Please ensure that your manuscript adheres to the AHA Journals' implementation of the Transparency and Openness Promotion (TOP) Guidelines (available online at https://www.ahajournals.org/content/TOP-guidelines).

For examples, please see
https://www.ahajournals.org/content/TOP-guidelines/ExampleDisclosures.

The Editors strongly encourage you to adhere to the journal's Statistical Reporting Recommendations in your revision, which can be found here: https://www.ahajournals.org/statistical-recommendations.

1. Reviewers' Critiques:

To read the comments to authors from the reviewers, please see below.
The editors concur with the statistical and technical reviewers and request that the requested changes be made.

Please include a detailed response to each of the referees' and editors' comments, providing each comment verbatim in bold followed by your response and giving the exact page number(s), paragraph(s), and line number(s) where each revision was made. If you make substantive changes to the manuscript, please provide a clear description of what you did and where. Additionally, a marked-up version of the revision with the changes highlighted or tracked should be uploaded as a supplemental file.

2. Formatting Issues:

Please ascertain that your revised manuscript adheres to the Instructions to Authors as they appear online at https://www.ahajournals.org/res/author-instructions. Accepted manuscripts are published online ahead of print. Therefore, when submitting the final files of the manuscript and figures, please ensure you have made any essential changes or corrections to content, grammar, and formatting. Please also ensure that author information provided in the online submission system is correct, including author order, proper names, and institutions. Once published ahead of print, you will be unable to make any revisions to the manuscript until you receive your author proofs from the publisher and any changes made to proofs will be reflected in the final print and online journal version of your article.

As your article may be published online upon acceptance, neither the Editorial Office nor the AHA will be responsible for any consequences with regard to intellectual property rights. To safeguard their intellectual property, authors should ensure that appropriate reports of invention and patent applications have been filed before the manuscript is accepted. If you should need to delay publication of your article for any reason, please let the Editorial Office know as soon as possible.

Please provide/address the following areas:

Manuscript Text:
- Please be sure to provide your revised manuscript text in an editable Word Doc file containing all sections of the manuscript, including tables and figure legends.
-Please include a Novelty and Significance section at the end of your Word file. Instructions for the Novelty and Significance section can be found at
https://www.ahajournals.org/res/revised-accepted-manuscripts.
We request that manuscripts published open access still adhere to the 8,000 word limit as much as possible. However, no excess charges will be rendered above the flat fee for open access publication. You may wish to move supplemental material to an online supplement, which can include supporting data and/or expanded text to offset the limits on the print version. Such online supplementary information can be cited in the print version as appropriate.
- Provide a list of nonstandard abbreviations and non-standard acronyms used in the manuscript text. The list should be included in the manuscript after the abstract and should be entitled "Non-standard Abbreviations and Acronyms." Circulation Research follows AMA style guidelines for standard and non-standard abbreviations.
- Authors are encouraged to provide a detailed, expanded Methods section as an online data supplement, especially if word limit constraints do not allow you to provide a detailed Methods section in the main manuscript. Methods sections should be detailed enough to enable readers to replicate the experiments without consulting previous articles.

Figures:
- Provide one full set of publication-quality figures as TIFF, PNG or JPG files. Please ensure that electronic figure files are in TIFF, PNG or JPG format and RGB color scale. Color and half-tone figures must have at least 600 dpi resolution; line drawings must have a 1200 dpi resolution or their original file format.
Online figures should be provided only in PDF format as part of the online supplement file.
-Authors of manuscripts that contain cropped gels/blots will be required to submit a separate PDF file that contains the entire unedited gel for all representative cropped gels in the manuscript. Authors should label each gel as "Full unedited gel for Figure _" and highlight which lanes of the unedited gel correspond to those shown in the cropped images within the manuscript. If the manuscript is accepted, the PDF of the unedited gels will be included in the online supplemental materials. For more information, please go to https://www.ahajournals.org/res/manuscript-preparation.
- There are no color figure charges for open access publication beyond the flat fee.

Online Supplement:
- Upload the online data supplement as one complete PDF (*not* a Word file) labeled "Supplemental Material" at the top of the first page.
-Supplementary Materials should not include a separate reference list and all citations in the Supplemental Materials should be included in the main reference list. In the manuscript text, following the Acknowledgments, Sources of Funding, & Disclosures section, please include a list of the supplemental materials with a callout to any references that are in the Supplemental Material only.
For example:
Supplemental Materials
Expanded Materials & Methods
Online Figures S1-S5
Online Video 1
Data Set
References 34-39

Other Items:
- A supplement containing a short tweet that can be used to promote the article and a 1-2 line 'lay sentence' similar to those provided for NIH grants.

The Editors strongly encourage you to submit potential cover images. Appropriate figures should be both aesthetically beautiful and scientifically exciting. Potential cover images should be associated with the general topic of the paper, or may be altered/enhanced versions of an original figure within the manuscript. Potential cover figures should have a single panel, with no labels or text of any kind. The figure file should be supplied at exactly 8 1/8" width by 10 7/8" height. Please submit figure initially as a low-resolution PDF. Include a figure legend with the figure. If your figure is chosen, we will request a high-resolution version (minimum of 600 DPI, RGB color format, and TIF or EPS file format).

We look forward to receiving the final revised version of your manuscript as soon as possible. Thank you for contributing to Circulation Research.

Check out the latest episode of Discover CircRes podcast for highlights from the latest issue and expert insights into new research.
New episode every month! https://www.ahajournals.org/res/podcast

Join us in Chicago on November 16-18 for #AHA24! Register here: https://ahascientificsessions.org/aha-24-registration.html

Sincerely,

Jane E. Freedman, MD
Editor-in-Chief
Circulation Research
An American Heart Association Journal

*******************************************************************************************
REVIEWER COMMENTS FOR THE AUTHORS:

**Reviewer #1:**
No further comments. The authors have addressed all my previous concerns, and the revisions have significantly improved the quality and clarity of the manuscript.

**Statistical Reviewer:**
1. Normality cannot be tested for when N is very small e.g. Less than 6. Please see reference below. Please explicitly state when relying on statistical properties for normality (e.g. the central limit theorem), explicitly state the property and what data it applies to.
Razali, N. M., & Wah, Y. B. (2011). Power Comparisons of Shapiro-Wilk, Kolmogorov-Smirnov, Lilliefors and Anderson-Darling Tests. Journal of Statistical Modeling and Analytics, 2, 21-33.
2. Please provide precise p-values (rather than P<0.0x). This can be obtained in GraphPad by increasing significant digits on the "Options" tab. Scientific notation with 2 significant figures (e.g. 1.2x10^-3) is strongly encouraged. This has not been addressed adequately.
3. You cannot make claims of no change or no effect. You can say no statistically significant difference was observed. You may not selectively present only some p-values that are not significant. Using n.s. would be preferable.
4. In figure 5, your conclusions comparing “lanes 4, 5, 6 & lanes 7, 8, 9” are not supported by the displayed statistical tests. The only p-valves presented are comparing to the control with no co-treatment. This should be a two-way anova with significant pairwise comparisons shown.

5. You may not set all controls arbitrarily equal to 1. Data should be normalized in a test standard way (e.g. total protein, B-actin, etc.). The AVERAGE of or a single datapoint may be scaled to 1, but variation must remain within the control groups.

**Technical Reviewer:**
The current study was carefully evaluated for inclusion of guideline items present in the Circulation Research checklists for rigor, transparency, and reproducibility. The reviewer has identified a number of items that were either omitted or not adequately addressed in the text. Please see below for details:

In vitro checklist items:
• Please update the figure numbers in the uncropped immunoblots to match the supplementary figure numbering.
• Please make sure that scale bars on all micrographs are of sufficient size to be seen when figures are reduced in size during production (both main and supplemental figures).
• Where immunostaining procedures are detailed, briefly describe the controls used to validate antibody specificity and distinguish genuine target staining from background (i.e. isotype vs secondary antibody only controls)

In vivo checklist items:
• Please provide additional information regarding the age, sex, and sources of mice. Were only male mice used for the study? If so, please provide a statement of rationale for the exclusion of females.
• Please provide more detail regarding dose of PCSK9 virus used.
• Is there a reason that C57BL/6N mice were used as controls for ob/ob mice since these are on the C57BL/6J background?

Other:
• How were representative images/figures chosen? Are they representative of the mean/average or the best/illustrative images? Please note this in the text somewhere.
• Where will the authors make their sequencing data public? Please provide database name and accession number.

Dear Professor Freedman,

**RE: CIRCRES/2023/324054/R3: EPAS1 Attenuates Atherosclerosis Initiation at Disturbed Flow Sites through Endothelial Fatty Acid Uptake**

We would like to thank you and the reviewers for your particularly thorough and helpful review of this manuscript. We were delighted to note that you see this paper as potentially acceptable and that we have been encouraged us to submit a revised version.

We have addressed the remaining minor comments from the Statistical Reviewer and the Technical Reviewer through additional statistical analysis and revision of the narrative.

Our manuscript is now much stronger and hopefully acceptable for publication in *Circulation Research.* Given the high novelty and significance of our manuscript we request that it is considered for an accompanying Editorial.

We submit a marked version (red font) and unmarked versions.

We have included a Novelty and Significance section and provided a short tweet that can be used to promote our article.

We have updated the Full Unedited Gel Figure file with additional arrows that identify which lanes have been used for the cropped gels in the manuscript.

We have submitted a potential cover image as a supplementary file. Image is supplied as a PDF file for initial low resolution at 8 1/8" width by 10 7/8" height as per requirements. A figure legend of the cover image is also provided as a supplementary file.

Our detailed response is given below:

**Reviewer #1:**

**No further comments. The authors have addressed all my previous concerns, and the revisions have significantly improved the quality and clarity of the manuscript.**

We would like to thank the reviewer for taking the time to revised both the original and revised versions of this manuscript and for having contributed to improving this work with their comments.

**Statistical Reviewer:**

**1. Normality cannot be tested for when N is very small e.g. Less than 6. Please see reference below. Please explicitly state when relying on statistical properties for normality (e.g. the central limit theorem), explicitly state the property and what data it applies to.**

**Razali, N. M., & Wah, Y. B. (2011). Power Comparisons of Shapiro-Wilk,**

**Kolmogorov-Smirnov, Lilliefors and Anderson-Darling Tests. Journal of Statistical Modeling and Analytics, 2, 21-33.**
We have thoroughly reviewed the statistical tests of each data set in our manuscript. We have also provided more clarification in the Methods under the statical analysis section (page 9, line 233-242). We performed the Shapiro-Wilk test for normality in all samples with N greater or equal to 6. If normality was not violated, we performed a parametric test. In case of violation of normality or N less than 6, we performed a nonparametric test, including Mann Whitney U test, Kruskal-Wallis or aligned ranked transformed ANOVA as appropriate. Updated p-values are presented in the revised version of the manuscript as exact p-values and the statistical test used is detailed in the figure legends.

**2. Please provide precise p-values (rather than P<0.0x). This can be obtained in GraphPad by increasing significant digits on the "Options" tab. Scientific notation with 2 significant figures (e.g. 1.2x10^-3) is strongly encouraged. This has not been addressed adequately.**
We have now updated our figures to show exact P values throughout our manuscript. In addition, scientific notation has been adopted for all p-values less than 0.00001.

**3. You cannot make claims of no change or no effect. You can say no statistically significant difference was observed. You may not selectively present only some p-values that are not significant. Using n.s. would be preferable.**
We have revised to narrative to ensure that we do not make claims of no change or effect. We have revised the figures to show p-values of described comparisons and used the notation n.s. when those described comparisons were not significant. To ensure that figures remain uncluttered and attractive, we have omitted n.s. values when the comparison was not described in the paper because it was unnecessary for the understanding of the data.

**4. In figure 5, your conclusions comparing "lanes 4, 5, 6 & lanes 7, 8, 9" are not supported by the displayed statistical tests. The only p-valves presented are comparing to the control with no co-treatment. This should be a two-way anova with significant pairwise comparisons shown.**
We used aligned ranked transformed ANOVA to analyse the data because we could not test for normality in this dataset (see above). The description of Figure 5A has been revised for accuracy (page 14).

**5. You may not set all controls arbitrarily equal to 1. Data should be normalized in a test standard way (e.g. total protein, B-actin, etc.). The AVERAGE of or a single datapoint may be scaled to 1, but variation must remain within the control groups.**
Our WB results are presented as normalised data to alpha tubulin or GAPDH and using a vehicle or selected control for relative quantification (after normalisation). We have corrected Fig. 5A by displaying the variability within the control values. These

values were achieved by plotting the relative value of each WB control against the average value of all the controls as alluded to by the reviewer.

**Technical Reviewer:**
**The current study was carefully evaluated for inclusion of guideline items present in the Circulation Research checklists for rigor, transparency, and reproducibility. The reviewer has identified a number of items that were either omitted or not adequately addressed in the text. Please see below for details:**

In vitro checklist items:

- **Please update the figure numbers in the uncropped immunoblots to match the supplementary figure numbering.**

We have revised the Supplemental file for uncropped immunoblots so that Figure numbering matches that of the Online Figures within the Supplemental Material.

- **Please make sure that scale bars on all micrographs are of sufficient size to be seen when figures are reduced in size during production (both main and supplemental figures).**

We have amended the size of the scale bars and font size to a sufficient size for reduction during production.

- **Where immunostaining procedures are detailed, briefly describe the controls used to validate antibody specificity and distinguish genuine target staining from background (i.e. isotype vs secondary antibody only controls)**

Throughout the research we made use of species-matched polyclonal IgG controls. In our previous revision of this manuscript, we listed the IgG controls in Supplementary Table 2 and in the Major Resources table. To be more explicit we have further revised the manuscript by explaining the use of IgG controls in the main body of the manuscript (page 7, lines 183-185) and in the Expanded Methods (Supplemental Material page 6, line 22 and page 10, line 7).

**In vivo checklist items:**

- **Please provide additional information regarding the age, sex, and sources of mice. Were only male mice used for the study? If so, please provide a statement of rationale for the exclusion of females.**

We used both male and female mice in this research. We have made a statement of this together with the age and sources in the Materials and Methods section of the main body of the manuscript (page 6, line 150). We have provided further details of the age and sex and sources of mice in the Expanded Methods section (pages 2-3) and in the supplementary Major Resource Table.

- **Please provide more detail regarding dose of PCSK9 virus used.**

This information is now provided in the Expanded Materials and Methods section Supplemental Material (page 2, lines 11-13) as follows:

"Virus particles were injected at a titre of $6*10^{11}$ in single dose to each mouse followed by a high fat diet (HFD; SDS UK, 829100) for 8 weeks as previously described[22]"

**• Is there a reason that C57BL/6N mice were used as controls for ob/ob mice since these are on the C57BL/6J background?**
We addressed this point in the previous response to reviewer comments. Ob/ob mice were compared to their littermate controls that were also on a BL6J background (Wild type mice in Figure 2). They were not compared to C57BL/6N background mice. Mouse genetic backgrounds are stated in the figure legends. To make this more explicit we have revised the Expanded Methods section (Supplemental Material page 2, paragraph 3, lines 21-22) as follows:

"$Lep^{ob/ob}$ mice (C57BL/6J) were obtained from The Jackson laboratory (strain: #00632) and were bred as heterozygotes. Wild-type littermates ($Lep^{WT}$) were used as controls."

**Other:**
**• How were representative images/figures chosen? Are they representative of the mean/average or the best/illustrative images? Please note this in the text somewhere.**
We addressed this point in the previous response to reviewer comments. We revised the manuscript to clarify this point as follows:

"Representative images were chosen to represent the average results achieved within the experiment" (main body of manuscript page 9, line 234).

**• Where will the authors make their sequencing data public? Please provide database name and accession number.**
Annotated data have been deposited in the Gene Expression Omnibus database (accession GSE274418) (manuscript main body, page 8, lines 189-191).
